# Computational Investigation of the Size Evolution of (La_2_ *B*_2_O_7_)_*n*_ Nanoclusters (*B* = Ce, Ti, Zr)

**DOI:** 10.1021/acsomega.5c06927

**Published:** 2025-10-10

**Authors:** Carina S. T. Peraça, Mauricio Mocelim, Mylena N. Santos, Juarez L. F. Da Silva

**Affiliations:** São Carlos Institute of Chemistry, University of São Paulo, Av. Trabalhador São-Carlense 400, 13560-970 São Carlos, SP, Brazil

## Abstract

Mixed-oxide particles are commonly used to promote chemical reactions in catalysis. However, our atomistic understanding of how particle size and oxygen vacancies influence their physicochemical characteristics remains limited. To address this issue, we use density functional theory calculations to investigate (La_2_ *B*_2_O_7_)_*n*_ nanoclusters, where *B* = Ti, Zr, Ce, and *n* = 2, 4, 6, 8, 10. Our findings and analysis reveal the following: (i) particle size plays a critical role in determining structural motifs, with all atoms in small particles (*n* = 2, 4) being entirely surface-exposed and exhibiting structural diversity, whereas larger clusters (*n* ≥ 6) develop bulk-like features in the core region with *B* cations located in the core and La segregating to the surface region; (ii) binding energy per atom increases with size, indicating enhanced stability resultant from diminished surface effects and compact structural motifs, with Zr-based nanoclusters demonstrating the strongest bonding; (iii) electronic band gaps decrease with increasing size, consistent with quantum confinement, although Ti- and Zr-based nanoclusters exhibit anomalies at intermediate sizes due to structural rearrangements; (iv) electrostatic potential analysis highlights highly positive cores in larger nanoclusters, elucidating their increased stability, while regions of low potential on the surface emerge as preferential sites for defect formation; (v) the formation of oxygen vacancy energetics follow to the hierarchy La_2_Ce_2_O_7_ < La_2_Ti_2_O_7_ < La_2_Zr_2_O_7_, with surface vacancies generally more stable than core ones, particularly in Ce-based nanoclusters; and (vi) vacancy-induced electronic and magnetic responses are significantly influenced by the *B* cation: Ce-based nanoclusters exhibit localized *f*-electron reduction and stable magnetic moments, Ti-based systems exhibit a mix of itinerant and polaronic behavior, and Zr-based clusters remain nonreducible and nonmagnetic.

## Graphical abstract

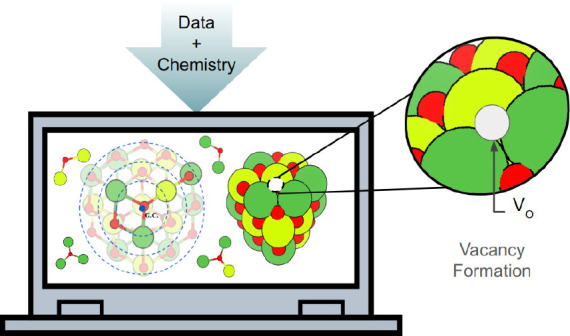

## 1 Introduction

Oxide compounds (*MO*_*x*_) comprise a highly versatile class of materials, which are characterized by interactions between electropositive metal ions (M) and highly electronegative oxygen ions (O^2–^). Their distinctive structural diversity and physicochemical properties form the foundation for applications in energy conversion, electronics, and catalysis.^1^ Specifically, oxides act not only as catalysts, but also as a support for transition metal particles, where they play a crucial role in stabilizing active centers and hence facilitate charge transfer and maintain high reactivity under extreme temperature and pressure conditions.^1,2^

A fundamental characteristic that drives the reactivity of oxides is the existence of oxygen vacancies, which impose substantial influences on electronic distribution, bond strengths, and local coordination environments, thus shaping the processes of chemisorption and catalytic outcomes.^3^ In the case of nonreducible oxides such as ZrO_2_, electrons remaining post oxygen removal tend to localize at the vacancy sites due to the significant Madelung potential, thereby increasing the local charge density and prompting structural rearrangements around the vacancy site.^4^ Conversely, in reducible oxides such as CeO_2_ ^5^ and TiO_2_,^6^ cation reduction occurs as excess electrons occupy *f*- or *d*-states, respectively, resulting in notable structural and electronic alterations. These phenomena may contribute to enhanced catalytic performance by stabilizing reactive intermediates, facilitating redox cycling, and enabling selective pathways for complex transformations.

Mixed oxides present particularly compelling opportunities whereby distinct cations interact to synergistically stabilize vacancies while modulating reactivity, stability, and electronic properties, among other characteristics. The family of La_2_ *B*_2_O_7_ pyrochlores (*B* = Ti, Zr, Ce) operates as a prime example of this concept,^7,8^ where the combination of the La_2_O_3_ (La^3+^) compound with various BO_2_ (*B* ^4+^) oxides has attracted significant scholarly attention.^9^ For instance, La_2_ *B*_2_O_7_ integrates the structural stability provided by La species with the variable redox properties of tetravalent cations, resulting in compounds that exhibit enhanced oxygen ion conductivity and tunable catalytic properties.^10^ Notably, La_2_ *B*_2_O_7_ oxides have been recognized as highly promising catalysts for the oxidative coupling of methane, wherein oxygen vacancies stabilize reactive oxygen species such as O^2–^, which are critical intermediates for methane activation and the formation of C2 hydrocarbons.^9,11^ Their defect chemistry and thermal stability position them at the vanguard of oxide-based catalysts for contemporary energy applications.

Despite significant advances in elucidating the nature of oxygen vacancies in bulk and surface oxides,^12,13^ our understanding of their influence in finite-sized systems such as nanoclusters remains far from satisfactory. Nanoclusters present distinctive opportunities owing to their reduced dimensions, which enhance surface-to-core ratios, thus permitting diverse vacancy configurations and structural rearrangements that are not feasible in very large particles.^14^ For instance, nanoparticles based on CeO_2_ and doped with trivalent cations exhibit higher vacancy concentrations and reduced defect formation energies when compared to their bulk counterparts.^15^ However, the degree to which these trends are applicable to mixed oxides such as La_2_ *B*_2_O_7_ at the nanocluster level remains largely unexplored. Addressing this gap is crucial because finite-size effects can change the stability, electronic structure, and catalytic performance compared to bulk phases.

A significant challenge within this domain is the design and characterization of the nanocluster structures. For example, the structural diversity that they exhibit, which originates from various potential atomic arrangements and stoichiometries, complicates the establishment of well-defined trends. In addition, their finite dimensions pose difficulties in identifying vacancy sites, core-surface segregation, and size-dependent electronic effects. In the absence of atomistic-level insights, our understanding of the evolution of nanoclusters with respect to composition and vacancy formation remains fragmented, thereby hindering the rational design of oxide-based catalysts on the nanoscale. To face these challenges, computational calculations based on density functional theory (DFT) present a robust methodological framework that facilitates a systematic exploration of nanoclusters, elucidating their stability landscapes and vacancy-induced modifications in a manner that remains beyond the reach of experimental techniques alone.

In this investigation, DFT calculations are used to characterize (La_2_ *B*_2_O_7_)_*n*_ nanoclusters (*B* = Ti, Zr, Ce; *n* = 2, 4, 6, 8, 10), with a specific emphasis on the evolution of the physical-chemical properties as a function of size and the role of oxygen vacancies. Our analyses indicate that the size of the nanoclusters governs the development of bulk-like cores, with lanthanum (La) preferentially segregating to surfaces and *B* cations contributing to the stabilization of core regions. Calculations of binding energies reveal that Zr-based clusters are the most stable, attributed to robust metal–oxygen interactions, while Ce-based clusters exhibit the lowest energies for vacancy formation, particularly at the surfaces. Moreover, distinct magnetic responses are uncovered: Ce nanoclusters manifest localized *f*-electron magnetism upon vacancy formation, Ti nanoclusters demonstrate size-dependent magnetic behavior, and Zr nanoclusters remain nonmagnetic, indicative of their resistance to vacancy formation. Together, these findings offer atomistic insight into the interrelation of size, composition, and defects within mixed-oxide nanoclusters, thereby opening new avenues for the strategic design of nanoscale catalysts.

## 2 Theoretical Approach and Computational Details

### 2.1 Total Energy Calculations

Our calculations were based on the spin-polarized DFT^16,17^ framework using the semilocal Perdew–Burke–Ernzerhof (PBE) formulation for the exchange-correlation (XC) energy functional.^18^ Although PBE is one of the most used approximations in computational material science,^19–21^ it can not provide an accurate description of particular properties of specific materials, such as the nature of the Ce *f*-states, which can change from delocalized (Ce^4+^ in CeO_2_) to localized (Ce^3+^ in Ce_2_O_3_).^22,23^ To improve the description of the localization of electronic states, we employed the PBE + *U* Dudarev rotational invariant approach,^24^ with an effective Hubbard parameter (*U*_eff_ = *U* – *J*) for each cationic species, namely, 4.5 eV for Ce,^22^ 3.0 eV for Ti,^25^ and 4.0 eV for Zr atoms,^26^ which were selected based on previous theoretical studies.^22,25,26^ Furthermore, the long-range interactions of the system were accounted for using the empirical vdW D3 ^27^ correction with the aim of correcting the binding energy and structural properties of the nanoclusters.^21,28^

The solution of the Kohn–Sham (KS)^17^ equations was obtained by expanding the KS orbitals in plane waves, while the interaction between valence and core electrons was described by the frozen-core projector augmented-wave (PAW) method,^29,30^ provided within the Vienna Ab initio Simulation Package (VASP),^30,31^ version 5.4.4. To ensure convergence, we used a plane wave cutoff energy of 488.734 eV, which is 12.5% higher than the maximum value recommended for the selected atomic species, namely La, Ce, Ti, Zr and O. The equilibrium structures were obtained once the atomic forces in each atom fell below 0.05 eV Å^–1^, together with the use of a total energy convergence parameter of 1 × 10^–5^ eV to achieve a self-consistent electron density during each iterative cycle.

From previous calculations,^32,33^ the nanoclusters were modeled within a cubic box with side lengths equal to *a* = 2*R*_c_ + 15 Å, where *R*_c_ is the radius of the nanocluster. Then, for the (La_2_ *B*_2_O_7_)_*n*_ nanoclusters with *B* = Ce, Ti, and Zr and *n* = 2, 4, 6, 8, and 10, the box dimensions were defined as 25.20, 28.48, 30.12, 32.08, and 34.36 Å, respectively. Finally, the Brillouin zone (BZ) integration was performed using only the Γ-point due to the absence of dispersion in the electronic states within the BZ. A Gaussian smearing parameter of 1 meV was used with the aim of obtaining an accurate description of the occupation of the electronic states close to the highest occupied molecular orbital, i.e., to avoid fractional electronic occupation.

For comparison, we calculated the bulk volume of La_2_ *B*_2_O_7_, using a pyrochlore structure for *B* = Zr^11^ and Ce,^34^ and a perovskite structure for *B* = Ti.^11^ A plane wave cutoff of 651.646 eV, which is 1.5 times larger than the highest recommended value for the selected atomic species (La, Ce, Ti, Zr, and O), was used to achieve convergence of the stress tensor as a function of the number of plane waves.^35^ The integration of the BZ was performed using a *k*-mesh of 3 × 4 × 2, 3 × 3 × 3, and 3 × 3 × 3 for *B* = Ti, Zr, and Ce, respectively. For subsequent bulk property calculations, we used the same cutoff energy as that used for nanocluster optimization. Additional details are reported in the electronic Supporting Information (SI) file.

### 2.2 Atomic Structure Configurations

As mentioned above, our computational investigation is centered on two goals: (i) Characterize the structural, energetic, and electronic attributes of mixed (La_2_ *B*_2_O_7_)_*n*_ nanoclusters as dependent on cluster size (*n*) and the specific identity of the *B* cation, namely Ti, Zr, and Ce; (*ii*) Characterize the formation of oxygen vacancies within these finite-sized mixed-oxide clusters and their impact on physicochemical properties. The accomplishment of these goals requires atomistic models of (La_2_ *B*_2_O_7_)_*n*_ nanoclusters, which are presently not well-represented in the existing literature due to the inherent complexities of multicomponent systems at the nanoscale regime. In contrast to bulk oxides, nanoclusters demonstrate properties that are size-dependent, resulting from high surface-to-core ratios, cation segregation, coordination variability, and an increase in surface reactivity. These characteristics, in conjunction with the potential for various structural motifs, surface reconstructions, and defect distributions, require detailed modeling of core–shell regions and probable sites for oxygen vacancies to encompass the full spectrum of structural and electronic phenomena. Thus, the formulation of nanocluster models constitutes the primary challenge in this research.

Building on our expertise in investigating nanoclusters of metals^36–41^ and oxides^15,42–45^ over several years, we combined a variety of methodologies and strategies to generate diverse structural configurations, each with varying conformations, thereby allowing the formation of unique chemical environments for the mixed La_2_ *B*_2_O_7_ nanoclusters. Herein, we delineate the principal steps involved in the synthesis of nanocluster structures.

1. The initial set of configurations for the (La_2_Ti_2_O_7_)_*n*_ nanocluster was produced using our Basin-Hopping Monte Carlo (BHMC) implementation (GOTNano),^46^ coupled with Coulomb and Buckingham pair-potentials.^47^ Although this method is influenced by the force-field bias, it yields configurations with reasonable atomic distances and coordination environments for the chemical species, as opposed to randomly generated atomic positions within a cubic box, particularly for large nanoclusters as those examined in this study. However, many structures generated during the BHMC optimization display similar environments, suggesting that performing DFT optimizations for such comparable configurations is not advisible due to the significant computational cost incurred.
2. The *k-means* clustering algorithm^48,49^ was employed in conjunction with the Coulomb matrix representation^50^ to select a subset of 10 × *n* (La_2_Ti_2_O_7_)_*n*_ representative configurations from the BHMC data set, which were preoptimized by DFT calculations using lower computational parameters, e.g., plane-wave cutoff energy and electron-density self-consistent criteria. From the set of 10 × *n* configurations, we observed many similar structures. Thus, to reduce the computational cost further, we employed *k-means* again to reduce this set to 5 × *n* trial configurations for (La_2_Ti_2_O_7_)_*n*_.
3. Based on our analysis, these structures resulted in a set of representative configurations, comprising trial structures of 10, 20, 30, 40, and 50 corresponding to *n* = 2, 4, 6, 8, and 10, respectively, where *B* = Ti, Zr, and Ce are applicable. Subsequently, all selected configurations were optimized using DFT calculations using the computational parameters defined in Section 2.1.

Consequently, the optimization of all selected structures using DFT represents one of the most resource-intensive undertakings in this study, attributable to the extensive array of configurations. Furthermore, it is important to keep in mind that our characterization is done for all optimized structures and not only for the lowest-energy structure.

## 3 Results and Discussion

To enhance the organization of the results and discussion, our findings are organized into four subsections: (*i*) Characterization of the most significant physicochemical attributes of all optimized (La_2_ *B*_2_O_7_)_*n*_ structures; (*ii*) Examination of the structural, electronic, and energetic properties of the lowest energy (La_2_ *B*_2_O_7_)_*n*_ configurations; (*iii*) Exploration of the effects induced by the formation of oxygen vacancies in these minimum energy structures; (*iv*) Finally, analysis of the mechanisms that underpin the stability and oxygen vacancy formation in the chosen nanoclusters.

### 3.1 Characterization of All Optimized Structures

In this section, we provide a systematic discussion of the most relevant physicochemical properties concerning the characterization of the studied nanoclusters.

#### 3.1.1 Energy Distribution Profile

To assess the effectiveness of our strategy to explore the space of structural possibilities, we evaluated the distribution of the total energies associated with the optimized configurations. Thus, to quantify the total energy distribution, we calculated the relative total energy per atom, Δ*E*_tot_, for each optimized structure. This metric is defined by the following equation:

$$\Delta E_{\mathrm{tot}}=(E_{\mathrm{tot}}^{i}-E_{\mathrm{tot}}^{\mathrm{lowest}})/11n$$ 1

where *E*_tot_^*i*^ is the total energy of a given configuration *i*, and *E*_tot_^lowest^ is the total energy of the most stable configuration within each group of nanoclusters having the same *n*. The factor 11*n* corresponds to the total number of atoms in the nanocluster, ensuring normalization between different sizes, which is critical for a fair comparison among nanoclusters with different sizes.

As shown in Figure 1, the Δ*E*_tot_ for each particle size exhibits a continuous decrease in optimized configurations. Notably, no plateau is observed in any of the distributions, indicating that each structure represents a distinct local minimum and that degenerate energy states are nearly absent. For the smallest systems (*n* = 2 and 4), the range of Δ*E*_tot_ reaches 145 meV/atom, reflecting significant structural variability due to the lower coordination environments compared to those of the larger nanoclusters. For example, for larger systems (*n* = 6, 8, and 10), the energy spread remains below 80 meV/atom, suggesting a more constrained configurational landscape as the size of the nanocluster increases, which is related to the structural characteristics of the initial structural configurations. This behavior is generally consistent across all cationic species.

**Figure 1:**
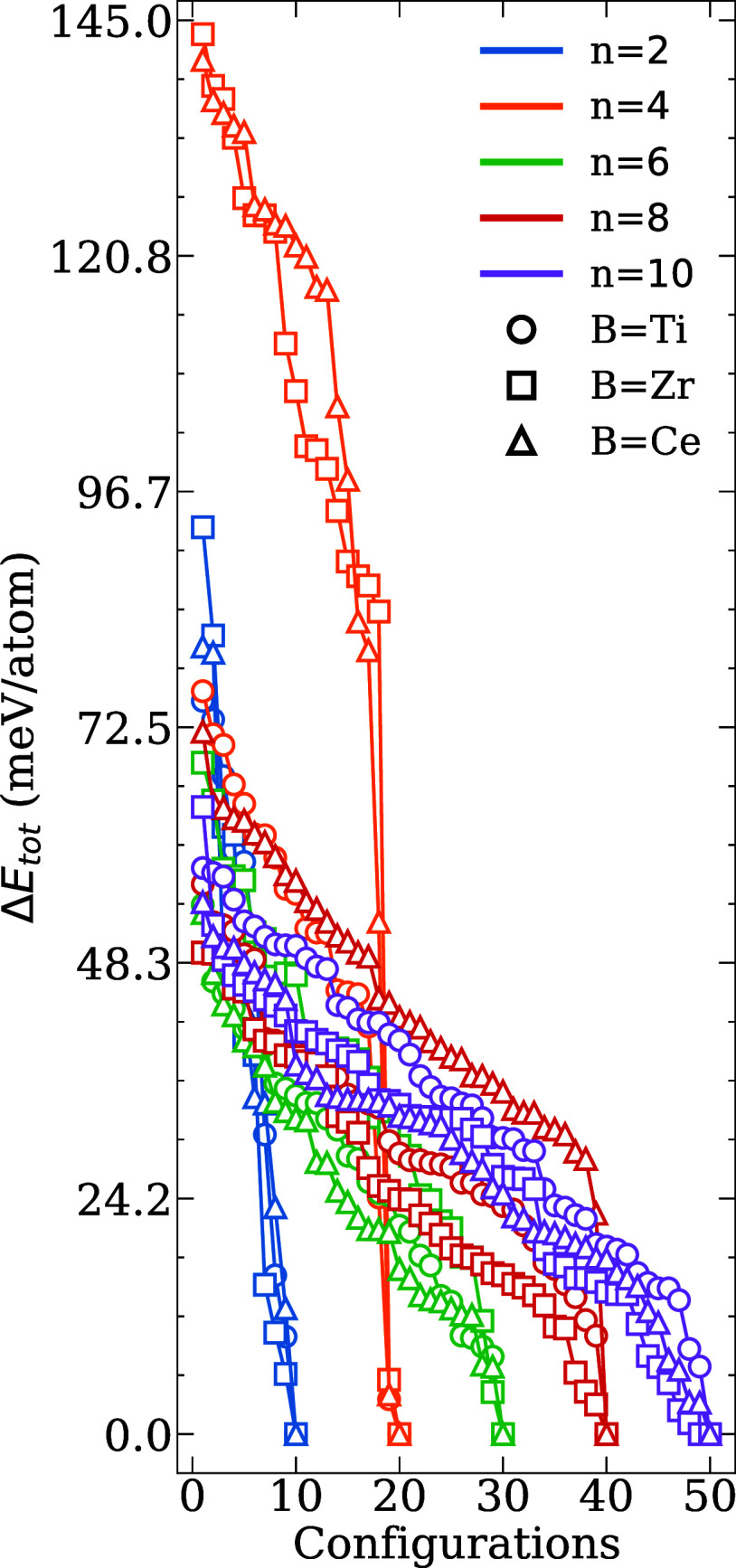
Relative total energy (Δ*E*_tot_) for the (La_2_ *B*_2_O_7_)_*n*_ nanoclusters for different values of *n* and for *B* = Ti, Zr, and Ce.

For all *n* values, the structural and energetic trends observed between *B* = Zr and Ce exhibit notable similarities, attributable to their comparable ionic radii, e.g., 0.60, 0.86, and 1.01 Å for Ti, Zr, and Ce, respectively.^51^ In contrast, substantial differences are evident when contrasting Zr- and Ce-based systems with those incorporating Ti. In the case of *n* ≤ 4, nanoclusters composed of *B* = Zr or Ce exhibit larger and more dispersed energy profiles compared to Ti nanoclusters. This phenomenon arises from the larger ionic radii of Zr^4+^ and Ce^4+^, which cause increased structural perturbations and configurational variability in small groups due to mismatches in packing and imbalances in coordination. However, as the size of the cluster increases, enhanced atomic coordination and stronger geometric constraints reduce these effects, resulting in more convergent and stable energy profiles across the three *B* species.

#### 3.1.2 Effect of Atomic Radius via Root Mean Square Deviation Analysis

To quantify the structural differences among the optimized structures for (La_2_Ti_2_O_7_)_*n*_, (La_2_Zr_2_O_7_)_*n*_, and (La_2_Ce_2_O_7_)_*n*_, we calculated the root-mean-square deviation (RMSD)^52,53^ between all corresponding optimized structures. The resulting RMSD values are plotted in Figure 2, for all nanocluster sizes. Our analysis reveals consistent and insightful trends across all nanocluster sizes. First, RMSD^Ce–Ti^ exhibits the highest values among the three pairwise comparisons, confirming that the substitution of Ti by a significantly larger Ce leads to the most pronounced structural changes. In contrast, RMSD^Zr–Ti^ values are consistently lower, suggesting that Zr-substituted structures retain a closer resemblance. As expected from their relative ionic radii, RMSD^Ce–Zr^ values fall between the two, further supporting the interpretation that the atomic size plays a dominant role in driving the observed deviations.

**Figure 2:**
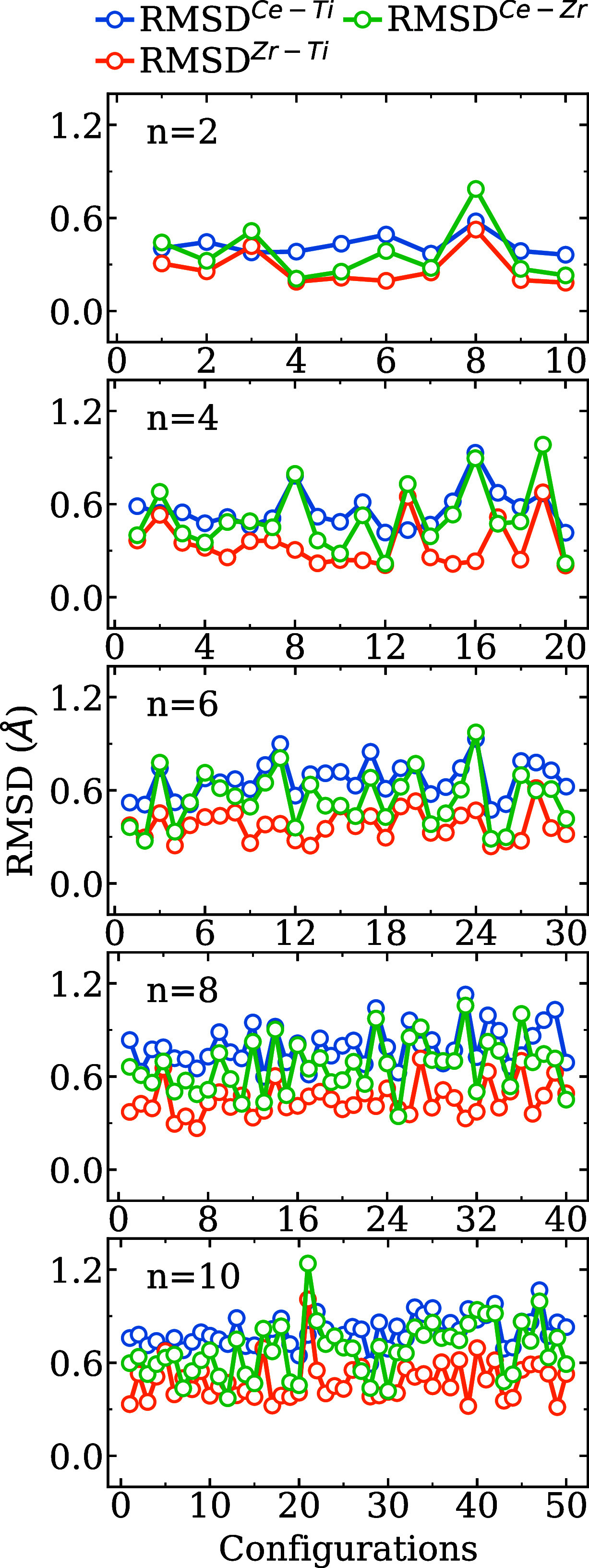
Root mean square deviation (RMSD) of structure configurations comparing (La_2_Ce_2_O_7_)_*n*_ and (La_2_Zr_2_O_7_)_*n*_ with (La_2_Ti_2_O_7_)_*n*_ (Ce–Ti and Zr–Ti), and (La_2_Ce_2_O_7_)_*n*_ with (La_2_Zr_2_O_7_)_*n*_ (Ce–Zr).

A clear size-dependent behavior is also evident: smaller nanoclusters (*n* = 2 and 4) show relatively modest RMSD values and low structural variability, with values typically below 0.90 Å. However, as *n* increases (*n* = 6, 8, and 10), the RMSD values not only increase in magnitude (ranging up to 1.2 Å) but also exhibit larger fluctuations between configurations, indicating a higher degree of structural rearrangement upon substitution of cations. This trend reflects the increasing structural complexity and configurational flexibility of the larger nanoclusters. Despite the general patterns, deviations from the expected order are observed in the specific configurations. In some cases, RMSD^Zr–Ti^ surpasses RMSD^Ce–Ti^, suggesting that structural rearrangements due to cation substitution are not dictated solely by ionic size. Furthermore, the known differences in the bulk crystal structures of La_2_Ti_2_O_7_, La_2_Zr_2_O_7_, and La_2_Ce_2_O_7_, as reported by Xu et al.,^11^ imply inherent preferences for different geometric arrangements that could influence the outcome of optimization, especially in nanoclusters approaching a larger number of atoms.

#### 3.1.3 Formation of Core Regions in Mixed-Oxide Nanoclusters

As the particle size increases, the distribution of chemical species within the core (atoms not exposed to vacuum) and surface (atoms exposed to vacuum) regions exhibits a size-dependent evolution. Consequently, considering the number of atoms in the core and surface domains, one can determine the minimum size required for the formation of a core region, an aspect essential to understanding the genesis of mixed-oxide nanoclusters. Using an in-house algorithm,^40^ the ratio of chemical species exposed to the vacuum and located at the core was calculated (*n* ^core^/*n* ^surf^). The results, which include the total number of atoms and the number of distinct species, are illustrated in Figure 3.

**Figure 3:**
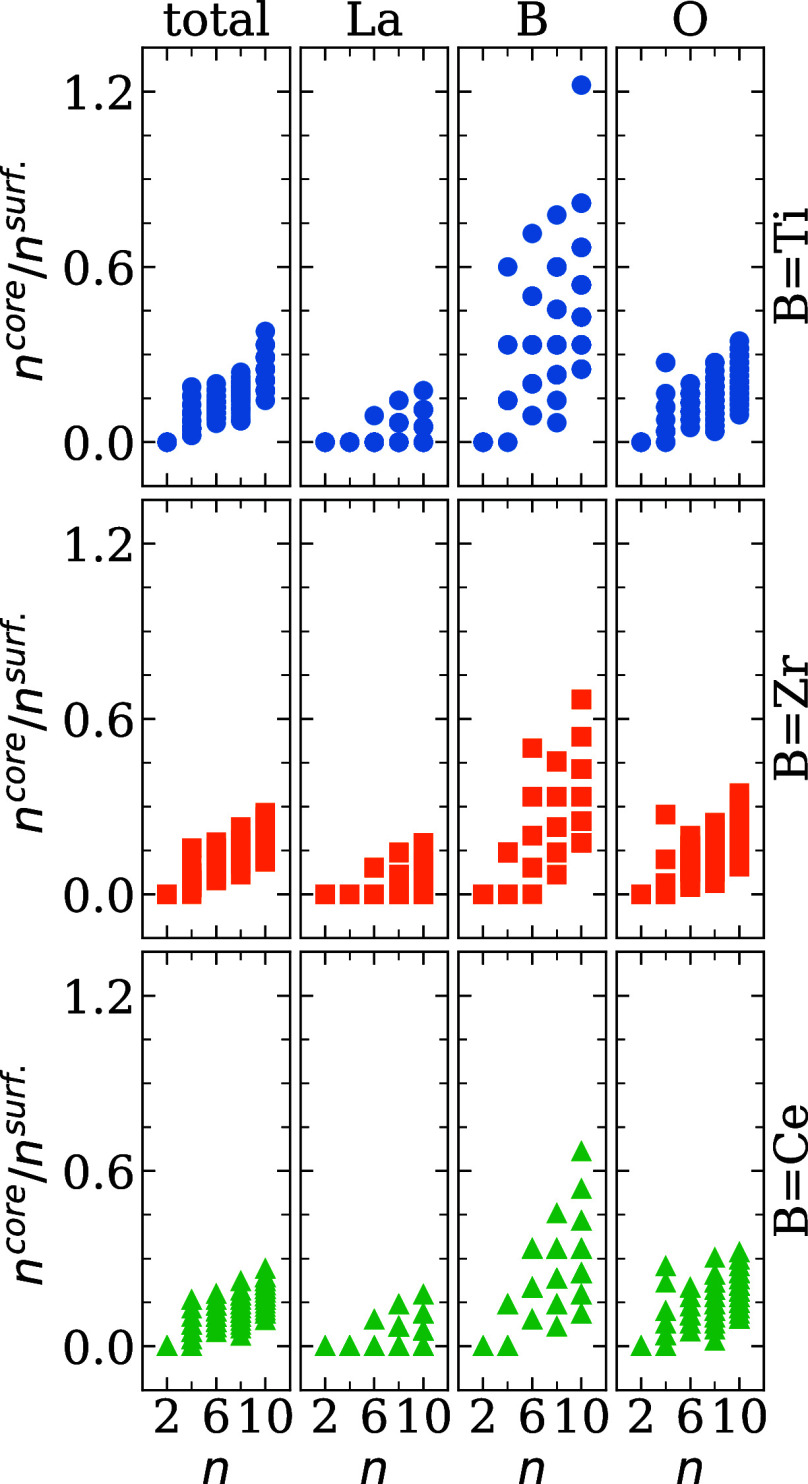
Ratio between the number of atoms located in the core and in the surface of the nanocluster, calculated for the total number of atoms in (La_2_ *B*_2_O_7_)_*n*_ structures as well as separately for each atomic species (La, B, and O), where *B* = Ti, Zr, or Ce, and *n* = 2, 4, 6, 8, and 10. Each dot indicates a result of a particular optimized structure.

For the smallest nanoclusters (*n* = 2), the ratio *n* ^core^/*n* ^surf^ of the total number of atoms is strictly zero in all configurations, indicating that all atoms are exposed to the vacuum region. This observation is consistent with the high surface-to-volume ratio expected for small nanoclusters. This explains the broad energetic distribution in Figure 1. Surface atoms typically experience lower coordination and higher reactivity, leading to a greater number of accessible local minima during structural optimization. As the size of the particles increases (*n* = 4), the ratio *n* ^core^/*n* ^surf^ spreads with values closer to zero (all atoms exposed to the vacuum) and up to 0.15 (starts the formation of the core region). This range suggests the beginning of geometric differentiation between the interior and exterior atomic environments. However, the small number of atoms is insufficient to stabilize a well-defined core, which again correlates with the relatively large energetic spread observed for *n* = 4 nanoclusters.

A notable change occurs for nanoclusters with *n* ≥ 6, where the distribution of *n* ^core^/*n* ^surf^ values begins far from zero, in most cases, and becomes broader and more continuous, with several configurations exceeding the threshold of *n* ^core^/*n* ^surf^ > 0.1. This transition indicates the emergence of more compact three-dimensional structures. Then, a subset of atoms can be consistently categorized as core atoms, that is, those with greater coordination and reduced exposure to the vacuum. The presence of such interior atoms generally correlates with increased structural stability and lower total energy variations. Furthermore, the dispersion in the ratio among configurations at a given *n* reveals the diversity of structural motifs that can be formed in this regime, depending on how atoms organize spatially to minimize energy under the constraint of finite size.

The distribution of *n* ^core^/*n* ^surf^ values for the La atoms indicates a particular pattern in the three compositions studied, namely *B* = Ti, Zr, and Ce. For the smallest nanoclusters (*n* = 2), the ratio *n* ^core^/*n* ^surf^ is zero since all atoms are exposed to the vacuum. A zero value of *n* ^core^/*n* ^surf^ was also observed for structures with *n* = 4. In this case, although the ratio involving total atoms indicates the beginning of the formation of the core region, we found that all La cations are located on the surface. The dispersion increases slightly from 0.00 to 0.15 for nanoclusters with *n* ≥ 6. Despite this increase, the appearance of values of *n* ^core^/*n* ^surf^ near zero indicates a tendency for La atoms to remain in the outermost layers of the nanocluster, regardless of the nanocluster size. This effect is attributed to the atomic radius of the La atoms being larger than that of the B species.

In contrast to La, the *n* ^core^/*n* ^surf^ ratio for B presents higher values, indicating a significant distribution of B atoms in the core region. For structures with *B* = Ti, nanoclusters with *n* = 4 showed a large variation in the *n* ^core^/*n* ^surf^ ratio, suggesting that nanocluster configurations can exhibit both structures in which all Ti atoms are located on the surface and others in which they also contribute to the core region. The variety of structural conformations in this set explains the range of energy values observed for nanoclusters of this size. The variation in the *n* ^core^/*n* ^surf^ ratio is even more pronounced for nanoclusters with *n* ≥ 6, ranging from 0.25 to 1.2 at *n* = 10, suggesting that B species tend to occupy the core region. In particular, in configurations, the number of Ti atoms in the core exceeds those on the surface. The distributions of Zr and Ce atoms in their respective nanoclusters show similar behavior, where smaller systems have a smaller radius that increases with the size of the nanocluster, indicating a tendency toward greater structural variation as the nanocluster grows.

In general, the *n* ^core^/*n* ^surf^ ratio for the O atoms exhibits behavior similar to that of the ratio for the total number of atoms due to the predominance of anionic species in the nanocluster. For all B configurations, the *n* ^core^/*n* ^surf^ ratio remains close to zero, varying from 0.00 to 0.35, indicating the tendency of these atoms to occupy regions near the surface. This can be attributed to the small size of the oxygen species and the resulting low coordination. As observed previously, the set with *n* = 4 shows a higher dispersion in the *n* ^core^/*n* ^surf^ ratio, reflecting a wide range of configurations at this size, which represents the point at which atoms begin to occupy regions closer to the core. With increasing nanocluster size (*n* ≥ 6), the ratio *n* ^core^/*n* ^surf^ becomes nonzero but remains relatively small, confirming a higher concentration of atoms on the surface than in the core.

#### 3.1.4 Coordination and Bonding Environment

To understand the local bonding environment and coordination behavior of atomic species within the (La_2_ *B*_2_O_7_)_*n*_ particles, we employed the effective coordination number concept,^54^ which provides a more realistic assessment of atomic environments in finite systems where discrete coordination shells and geometric distortions are prevalent. Using this formalism, the effective coordination number (ECN) for an atom *i* and the average weighted bond lengths are defined by the following equations,

$${\mathrm{ECN}}^{i}=\sum_{\begin{array}{c} j=1\\ i\neq j \end{array}}\exp\left[1-{\left(\frac{2d_{ij}}{d_{\mathrm{av}}^{i}+d_{\mathrm{av}}^{j}}\right)}^{6}\right]$$ 2

where

$$d_{\mathrm{av}}^{i}=\frac{\sum_{\begin{array}{c} j=1\\ i\neq j \end{array}}d_{ij}\exp\left[1-{\left(\frac{2d_{ij}}{d_{\mathrm{av}}^{i,\mathrm{old}}+d_{\mathrm{av}}^{j,\mathrm{old}}}\right)}^{6}\right]}{\sum_{\begin{array}{c} j=1\\ i\neq j \end{array}}\exp\left[1-{\left(\frac{2d_{ij}}{d_{\mathrm{av}}^{i,\mathrm{old}}+d_{\mathrm{av}}^{j,\mathrm{old}}}\right)}^{6}\right]}$$ 3

where the starting values for *d*_av_^*i*,old^ and *d*_av_^*j*,old^ are the smallest distances between the *i* and *j* atoms, which are updated during the self-consistent approach, until the obtaining or convergence criteria (|*d*_av_^*j*,old^ – *d*_av_^*i*,old^| < 10^–4^). The exponential weight function ensures that more significant contributions are given to shorter, chemically relevant bonds.

These metrics were computed for each atomic species, namely, La, B, and O, for all *n* values. Figure 4 shows that the *d*_av_^*i*^ values display a clear size-dependent behavior, with shorter average bond distances for smaller nanoclusters (*n* = 2) and a nearly constant profile from *n* = 4 onward. Among the three cationic species, the Ce-based nanoclusters consistently exhibit the longest bond distances, followed by Zr, and then Ti, consistent with the increasing order of atomic radii (Ti < Zr < Ce).^51^ The *d*_av_^*i*^ values of La and O tend to approximate each other, especially for systems with *B* = Zr and Ce. This result is supported by the tendency of these species to occupy the same region in the nanoclusters (close to the surface), as indicated by the small values of *n* ^core^/*n* ^surf^.

**Figure 4:**
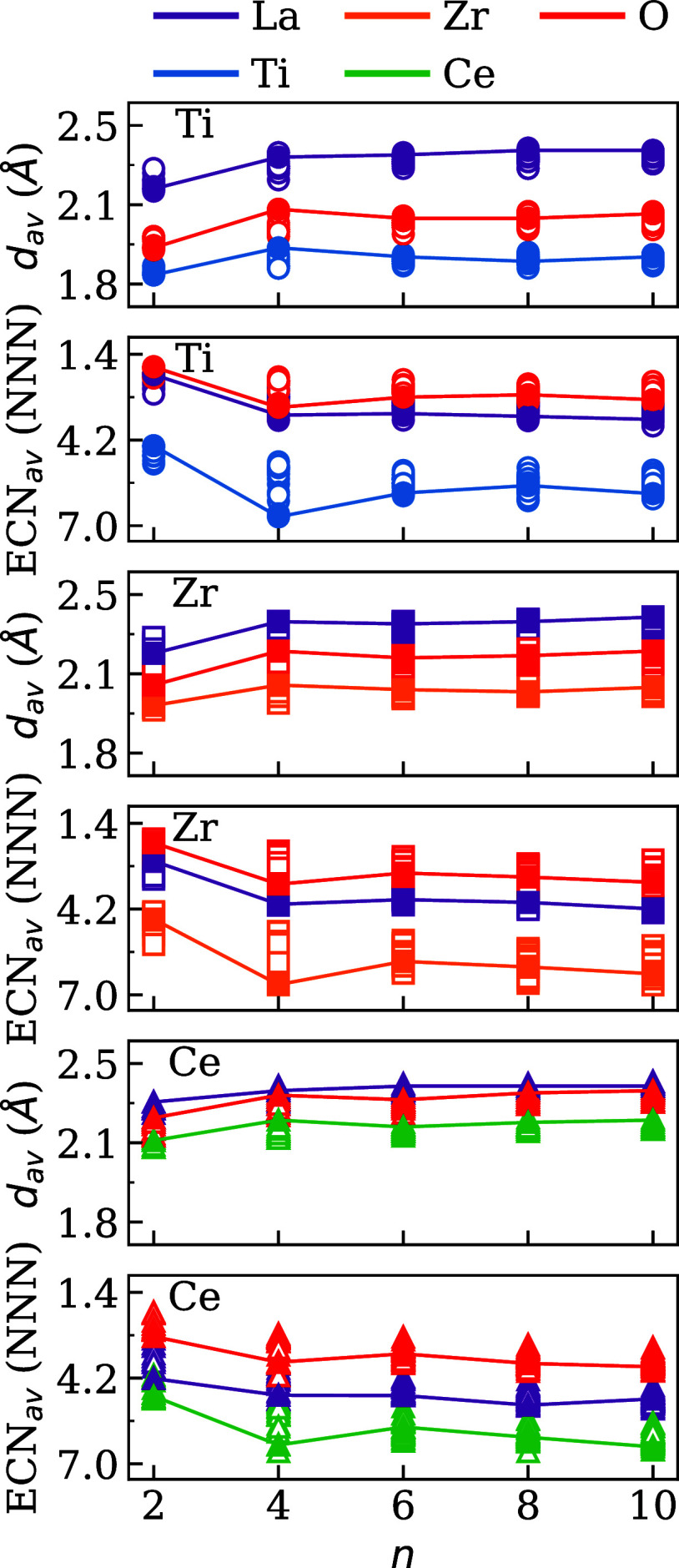
Average bond distance (*d*_av_) and average effective coordination number (ECN_av_) for atomic species of (La_2_ *B*_2_O_7_)_*n*_ nanoclusters with *B* = Ti, Zr, and Ce.

The average ECN values (ECN_av_) reveal a complementary trend with respect to the bond distances. For the smallest nanoclusters (*n* = 2), all atomic species show low coordination numbers, consistent with their complete exposure to the vacuum region. This behavior is consistent with previous observations in the core-to-surface atom ratio analysis; Figure 3, which showed *n* ^core^/*n* ^surf^ = 0 for *n* = 2. As the size of the nanocluster increases, the ECN_av_ values also increase for all atomic types, indicating the progressive development of internal coordination environments. The shift becomes more significant for *n* ≥ 6, where atoms begin to populate the nanocluster core, stabilizing compact three-dimensional motifs.

An intriguing deviation occurs at *n* = 4, where a modest increase in the level of ECN_av_ is observed, especially in the La and B atoms. This is consistent with a particularly symmetric and compact configuration that locally maximizes coordination despite its overall small size. These configurations also exhibited higher values of *n* ^core^/*n* ^surf^, suggesting the formation of incipient core regions, even at small nanocluster sizes. However, because of the limited atom count, the increase in coordination is not as pronounced as in larger groups and does not translate into the same degree of energetic stabilization. For *n* ≥ 6, both *d*_av_ and ECN_av_ exhibit more consistent and material-dependent trends, reflecting structural convergence. The emergence of a stable core and the higher ECN contribute to the reduced energetic variability among configurations, as seen in the narrower energy distributions. In particular, the Ce-based nanoclusters maintain a lower ECN_av_ compared to their Zr and Ti counterparts, which may reflect both the larger size of the Ce atoms and the more flexible bonding network required to accommodate them.

#### 3.1.5 Energetic Stability via Binding Energy

To evaluate the energetic stability of the mixed oxide particles, we calculated the average binding energy per atom (*E*_b_), as a metric of the energetic stability. Then, *E*_b_ can be calculated using the following equation,

$$E_{b}=\frac{1}{N_{\mathrm{tot}}}\left(E_{\mathrm{tot}}^{\mathrm{cluster}}-\sum_{i}N_{i}E_{\mathrm{tot}}^{i}\right)$$ 4

where *E*_tot_^cluster^ denotes the total energy of the nanocluster, and *E*_tot_^*i*^ is the total energy of a single atom in vacuum, expressed as *i* = Ti, Zr, Ce, La, O. Here, *N*_*i*_ represents the number of atoms for each chemical element *i*, while *N*_tot_ indicates the total number of atoms present in the particles. Equation 4 calculates the energy required to completely separate atoms in the structure, which means that a high value of *E*_b_ corresponds to strongly bonded atoms, leading to greater stability. The same concept was used to calculate *E*_b_ for the bulk phases to facilitate a comparative analysis.

Figure 5 shows the *E*_b_ for (La_2_ *B*_2_O_7_)_*n*_ as a function of particle size (*n* = 2, 4, 6, 8, and 10) and *B* = Ti, Zr, and Ce. Our results show a decrease in *E*_b_ as the *n* value increases, reflecting greater stability in higher nanoclusters. This behavior is consistent across all three systems and aligns with expectations from surface-to-core ratio effects. As *n* increases, the *n* ^core^/*n* ^surf^ ratio increases, reducing the influence of lower coordinated surface atoms and favoring the formation of core regions. Thus, it also explains the lower binding energies (higher stability) for the bulk phases, as indicated by the dashed horizontal lines in Figure 5. In addition to the difference of approximately 0.60 eV between the nanocluster and bulk binding energy, the order of stability of each compound is in accordance with that of their respective bulk counterparts.

**Figure 5:**
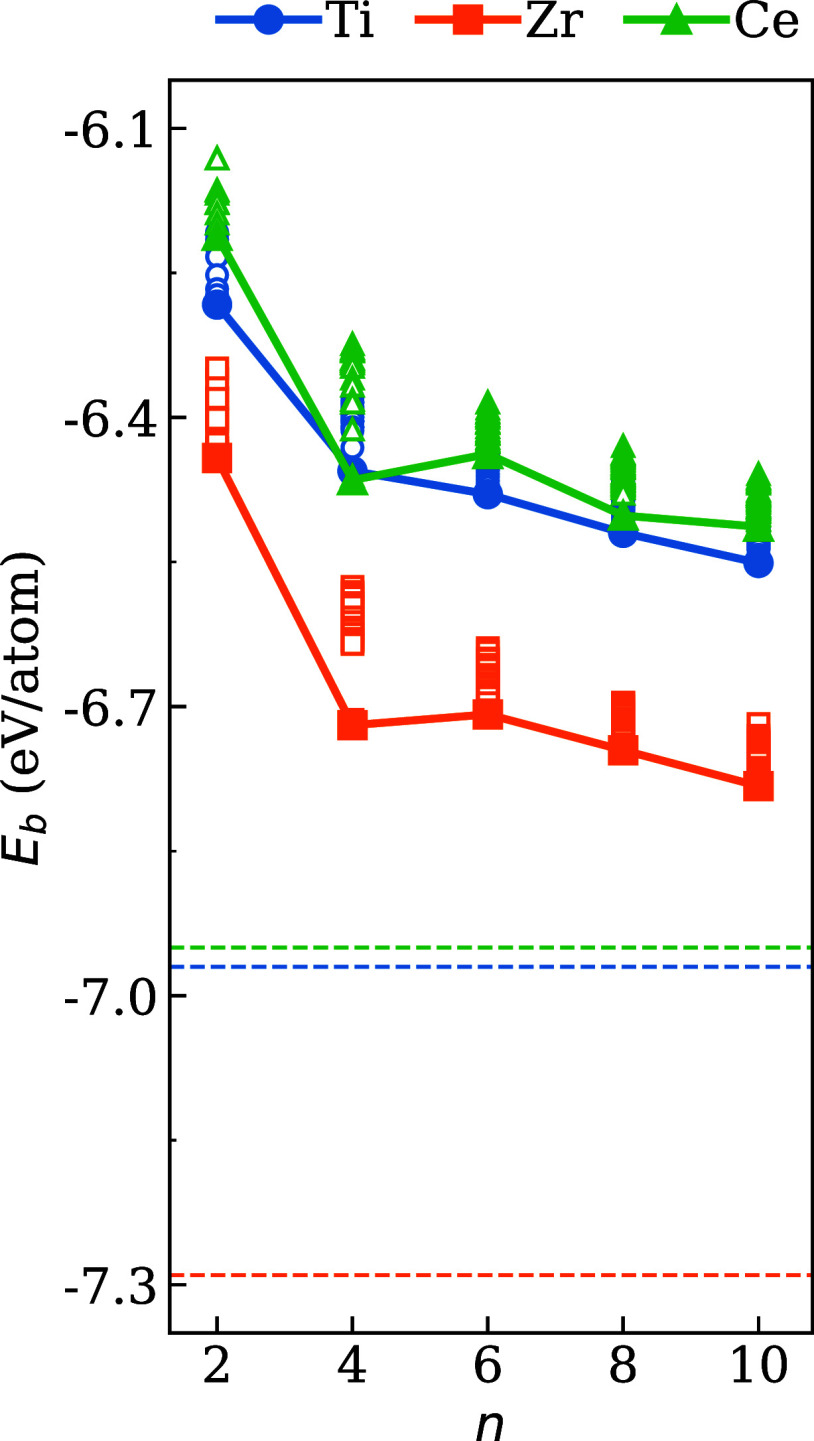
Binding energy (*E*_b_) of the optimized (La_2_ *B*_2_O_7_)_*n*_ structures for *B* = Zr, Ti and Ce as a function of the particle size (*n* = 2, 4, 6, 8 10). The filled marks represent the lowest energy configuration, and the dashed horizontal lines indicate the oxides in bulk phase.

In particular, a deviation from this trend is identified in *n* = 4 in all systems, particularly within the nanoclusters with *B* = Ti and Ce. This anomaly signifies a local stabilization, potentially attributable to the emergence of compact, energetically favorable core-like motifs at this intermediate size. This observation is consistent with the increase in coordination number (ECN_av_) in *n* = 4, which is correlated to a higher packing efficiency and a decrease in surface stress.

The differences among the three systems can be rationalized based on the chemical nature and bonding preferences of the *B* site cations: the systems based on (*i*) Zr exhibit the most negative binding energies, indicating the strongest metal–oxygen bonds. This aligns with the high charge density of Zr^4+^, which makes it a hard acid and facilitates strong electrostatic interactions with oxygen, a hard base, resulting in high lattice energy in zirconium oxides. (*ii*) Ti- and Ce-based systems show smaller and similar *E*_b_ due to their lower affinity for O and the reduction capacity of their oxidation states, leading to the weakening of the chemical bonds Ti and Ce–O.

#### 3.1.6 Electronic Fundamental Energy Band Gap

The fundamental electronic band gap (*E*_g_) is determined as the energy separation between the highest occupied molecular orbital (HOMO) and the lowest unoccupied molecular orbital (LUMO). As expected, in finite systems, *E*_g_ is substantially influenced by the impacts of quantum confinement due to the particle sizes, structure features, and orbital hybridization.^55^ Figure 6 shows the evolution of *E*_g_ as a function of the number of formula units *n* for (La_2_ *B*_2_O_7_)_*n*_ with *B* = Ti, Zr, and Ce for all optimized structures, including the lowest energy and higher energy structures. In addition, for comparative purposes, values obtained for the bulk phases are also presented.

**Figure 6:**
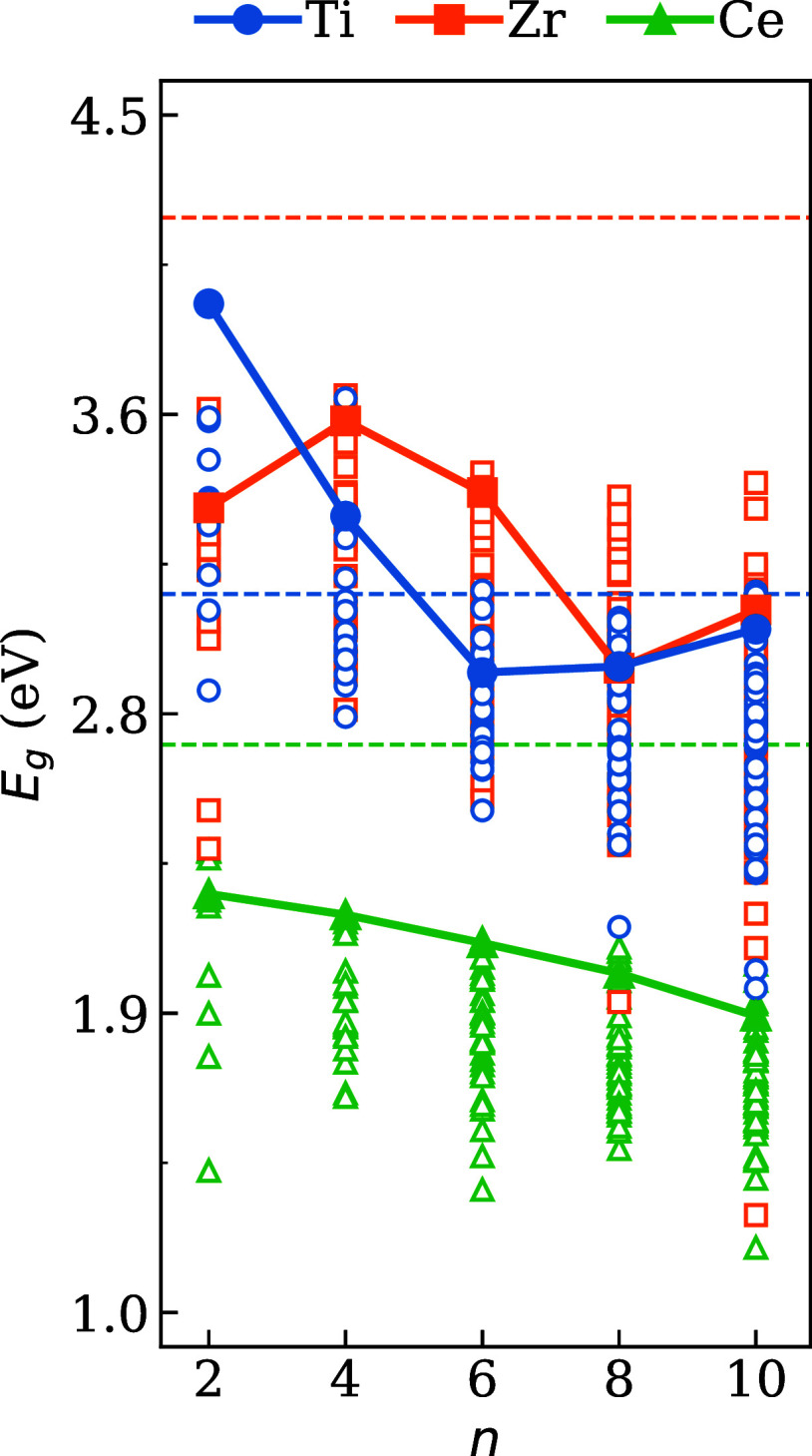
Fundamental electronic band gap (*E*_g_) of the optimized (La_2_ *B*_2_O_7_)_*n*_ structures for *B* = Zr, Ti and Ce as a function of the particle size (*n* = 2, 4, 6, 8 10). The filled marks represents the lowest energy configuration and the dashed horizontal lines indicate the oxides in bulk phase. The higher energy configurations are denoted by open symbols.

For *B* = Ce and Zr, the largest *E*_g_ values are associated with the structures of the lowest energy, whereas the structures of the highest energy exhibit reduced band gaps. In addition, as the value of *n* increases, a reduction in the band gap is observed. However, this does not trend toward the bulk value. Smaller nanoclusters, that is, with few atoms, tend to have a lower gap value compared to the bulk.^42^ However, in systems such as Ti, oscillations are expected as *E*_g_ approaches the bulk values for significantly large values of *n*, showing a reduction in *E*_g_ as the size increases, although this decline is characterized by less consistency. These systems showed a marked reduction in the band gap from *n* = 2 to *n* = 6, which subsequently led to a plateau indicative of the emergence of bulk-like behavior. The bandgap oscillation of nanoclusters based on Ti close to the bulk is explained by the tendency of structures based on TiO_2_ to form stable structural units, even on a reduced scale, which maintains their electronic structure close to that of the bulk.^56^

A similar crossover is seen for the nanoclusters based on Zr. Although their *E*_g_ values generally follow a decreasing trend, they exhibit a local maximum at *n* = 4. This anomaly correlates with the drop in binding energy observed at the same size, Figure 5, suggesting a structural rearrangement or an improved location of the frontier orbitals. This behavior mirrors that described by Zibordi-Besse et al.,^42^ who observed that certain configurations (ZrO_2_)_*n*_ can stabilize electronic states that deviate from bulk-like trends due to the formation of specific coordination geometries or bonding motifs.

### 3.2 Lowest Energy Structures

From this point, our analysis focuses on the characterization and discussion of the lowest energy (La_2_ *B*_2_O_7_)_*n*_ structures, as shown in Figure 7. Table 1 provides a summary of the principal structural properties of these nanoclusters.

**Figure 7:**
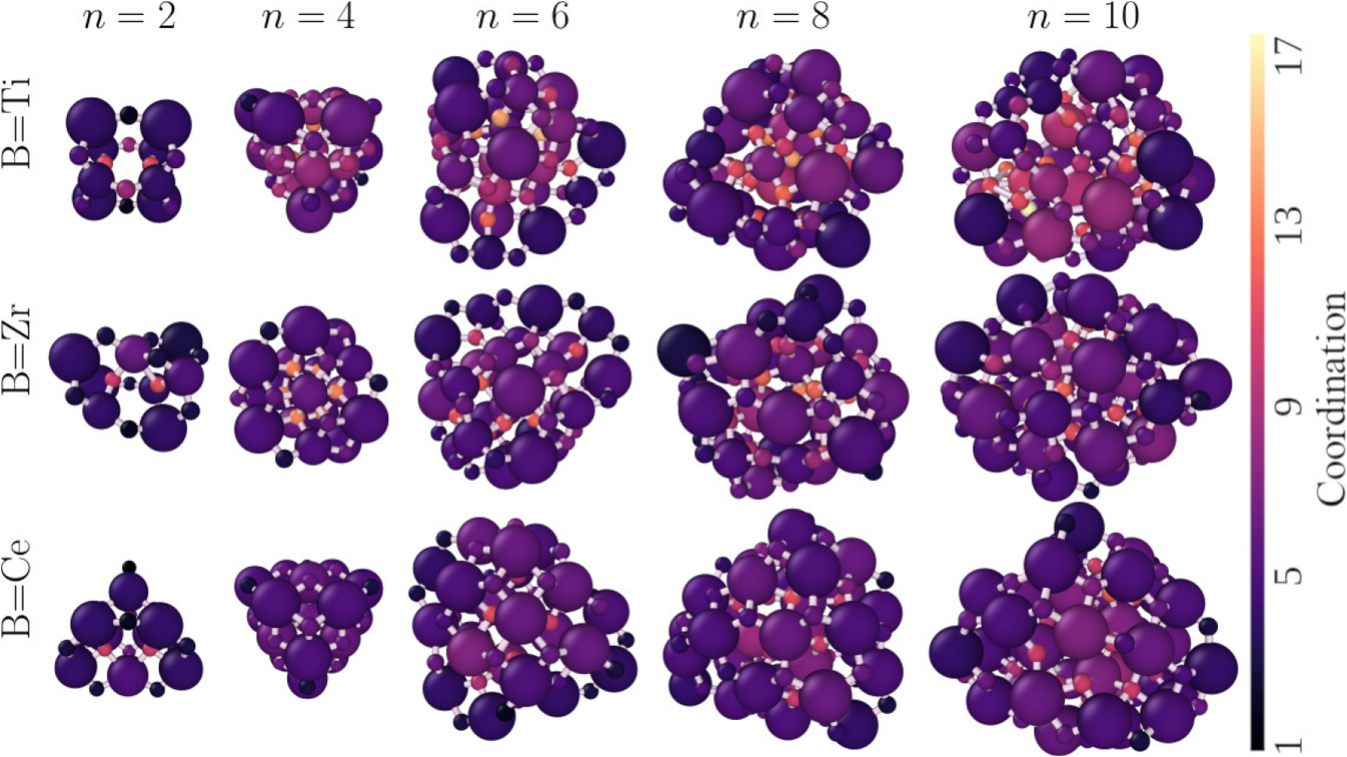
Lowest energy (La_2_ *B*_2_O_7_)_*n*_ structures for different *B* species and different values of *n*. Dark colors represent low atomic coordination.

**Table 1:** Selected Structural Parameters Calculated for the Lowest Energy (La_2_ *B*_2_O_7_)_*n*_ Nanoclusters^a^

*B*	*n*	*n* ^core^	*n* ^surf^	*n*_La_^core^	*n*_B_^core^	*n*_O_^core^	*n*_La_^surf^	*n*_B_^surf^	*n*_O_^surf^
Ti	2	0	22	0	0	0	4	4	14
	4	6	38	0	0	6	8	8	22
	6	10	56	0	3	7	12	9	35
	8	12	76	1	3	8	15	13	48
	10	20	90	2	7	11	18	13	59
Zr	2	0	22	0	0	0	4	4	14
	4	6	38	0	0	6	8	8	22
	6	8	58	0	2	6	12	10	36
	8	11	77	1	3	7	15	13	49
	10	23	87	0	6	17	20	14	56
Ce	2	0	22	0	0	0	4	4	14
	4	6	38	0	0	6	8	8	22
	6	9	57	0	2	7	12	10	35
	8	14	74	2	2	10	14	14	46
	10	21	89	0	5	16	20	15	54

a Total number of atoms in core (*n* ^core^) and surface (*n* ^surf^) region; Total number of La, B and O atoms located in the core region (*n*_La_^core^, *n*_B_^core^, *n*_O_^core^), respectively; Total number of La, B and O atoms located in the surface region (*n*_La_^surf^, *n*_B_^surf^, *n*_O_^surf^), respectively.

#### 3.2.1 Structural Features: Morphology, Coordination, and Local Environments

Materials within the nanocluster phase can exhibit varied morphologies and characteristics, depending on their size and chemical composition. Figure 1 shows that nanoclusters with an identical number of atoms and composed of the same chemical species can give rise to a wide spectrum of energy levels, indicating substantial structural variations among configurations within the same ensemble. However, we observed certain consistent attributes that correlate with the most stable structures, as shown in Figure 7.

Small nanoclusters (*n* ≤ 4) demonstrated low coordination for most atoms, since many of these atoms, particularly when *n* = 2, are exposed to the vacuum region. In the case of *n* = 4, all cationic species are located on the surface, as seen in Table 1. Within these systems, a slight increase in coordination was noted as the smaller atoms progressed toward the geometric center of the structure. This phenomenon was most apparent in structures that possess *n* = 4 and *B* = Ti, where the cationic species exhibited a smaller atomic radius compared to those of *B* = Ce and Zr. In addition, systems that incorporate *n* ≤ 4 tend to demonstrate greater structural symmetry as the difference between the atomic radii of *B* and La decreases.

As particle sizes expand (*n* ≥ 6), the structures tend to exhibit a decreased symmetry but a notable increase in the number of highly coordinated atoms in the core region, particularly oxygen. Structures containing *B* = Ti comprised 12, 15, 18 La atoms on the surface corresponding to *n* = 6, 8, and 10, followed by structures with *B* = Zr, which incorporated 12, 16, 20 La atoms, and *B* = Ce, which demonstrated 12, 14, 20 La atoms for identical nanocluster sizes, as can be seen in Table 1. Currently, the presence of *B* atomic species on the surface tends to decrease as the atomic radii of its species decrease, reaching the lowest levels when *B* = Ti.

#### 3.2.2 Atomic Species Distribution via Radial Distribution Function

The probability of occurrence of atomic species in specific regions of the nanocluster, as well as its distribution throughout the structure, is verified. The radial distribution function *g*(*r*) represents the local density of neighboring atoms *j* around a reference atom *i*, normalized by the average density. Mathematically, it can be expressed by the following equation,

$$g\left(r\right)=\frac{\left\langle \rho \left(r,r^{\prime }\right)\right\rangle }{\rho }$$ 5

where ρ is the average particle density, obtained by

$$\rho =\frac{N}{V}$$ 6

with *N* and *V* being, respectively, the number of atoms and the volume of distribution. Then, ρ­(*r*, *r′*) is the correlation density function of the spatial correlation between two particles, given by

$$\rho \left(r,r^{\prime }\right)=\left\langle \overset{N}{\underset{i=1}{\sum }}\delta \left(r-r_{i}\right)\overset{N}{\underset{j=1}{\sum }}\delta \left(r-r_{j}\right)\right\rangle$$ 7

The calculation of *g*(*r*) for the La, O, and B species was performed using the OVITO software.^57^ The results are depicted in Figure 8.

**Figure 8:**
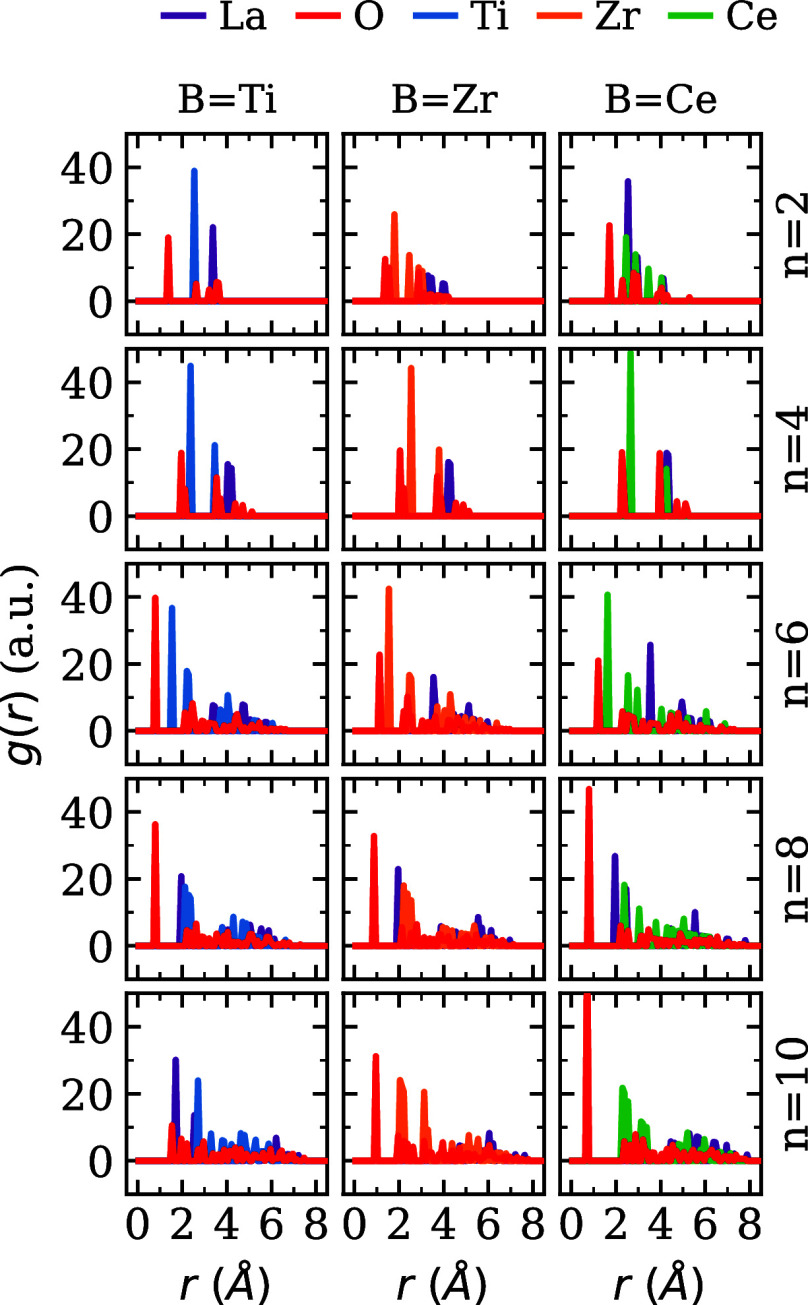
Radial distribution function of (La_2_ *B*_2_O_7_)_*n*_ configurations with *B* = Zr, Ti and Ce, and *n* = 2, 4, 6, 8 and 10.

In smaller systems, characterized by *n* = 2 and 4, the peaks exhibit a propensity toward regularity, consistent with the symmetry observed in groups of this dimension, as depicted in Figure 7. For systems with *n* = 2, there exists a high probability that the O atoms are situated near the geometric center of the structure, in close proximity to the cationic species, attributed to the exposure of all atoms to the vacuum. In structures incorporating *B* = Ce and Zr, a more extensive distribution of cations is observed compared to systems with *B* = Ti, which possess the smallest atomic radius among the three. For nanoclusters defined by *n* = 4, there is a distribution of O atoms throughout the structure, with a high probability of locating *B* atoms at distances ranging between 2 and 3 Å. In contrast, La atoms are more likely to be found at greater distances, corroborating their presence in the surface region, as demonstrated in Figure 3.

An increase in particle size to *n* = 6 has resulted in an elevated probability that *B* cations are located nearer to the geometric center, attributed to their atomic radii being intermediate in size relative to the radii of the O and La atoms. This observation corroborates the findings depicted in Figure 3, wherein the ratio *n* ^core^/*n* ^surf^ increases for the atoms of this species. Consequently, in larger nanoclusters (*n* = 8 and 10), the probability of situating cationic species at proximate distances from the geometric center of the nanocluster (less than 2 Å) is also corroborated, notably for *B* = Ti, which possesses the smallest radii among the three cations. Generally, the O atoms tend to be homogeneously dispersed to maintain the electrical neutrality of the nanocluster, as observed by Mocelim et al. in large (La_2_Ce_2_O_7_)_*n*_, (La_4_O_6_)_*n*_, and (Ce_4_O_8_)_*n*_ nanoclusters.^45^

#### 3.2.3 Mapping Local Electrostatic Potential Environments

The electrostatic potential energy can play a role in the formation of materials, influencing the binding energy of a system and the formation of defects.^58^ For this reason, we aim to map the electrostatic potential of the (La_2_ *B*_2_O_7_)_*n*_ lowest-energy structures. We evaluated this property using the smooth particle-mesh Ewald method (PME),^59^ as implemented in the Visual Molecular Dynamics (VMD) software.^60^ This approach considers the charge *q* and the van der Waals radius *r* to solve the Poisson equation on a numerical grid, evaluate long-range electrostatic interactions, and generate a map of electrostatic potential (MEP).^15^ Within this framework, all atomic species of the structure are modeled as spheres, with charge distributed on their surfaces. Our PME calculations were performed considering the local effective atomic charge calculated by VASP.

The results shown in Figure 9 indicate that clusters with *n* = 2 and 4 exhibit intermediate values of electrostatic potential (0.64 V) due to the high symmetry of these structures and the low coordination difference among the atoms. An exception occurs in systems where *B* = Ti, since the difference between the atomic radii of the cations is greater compared to *B* = Ce and Zr, affecting the coordination and charge distribution.

**Figure 9:**
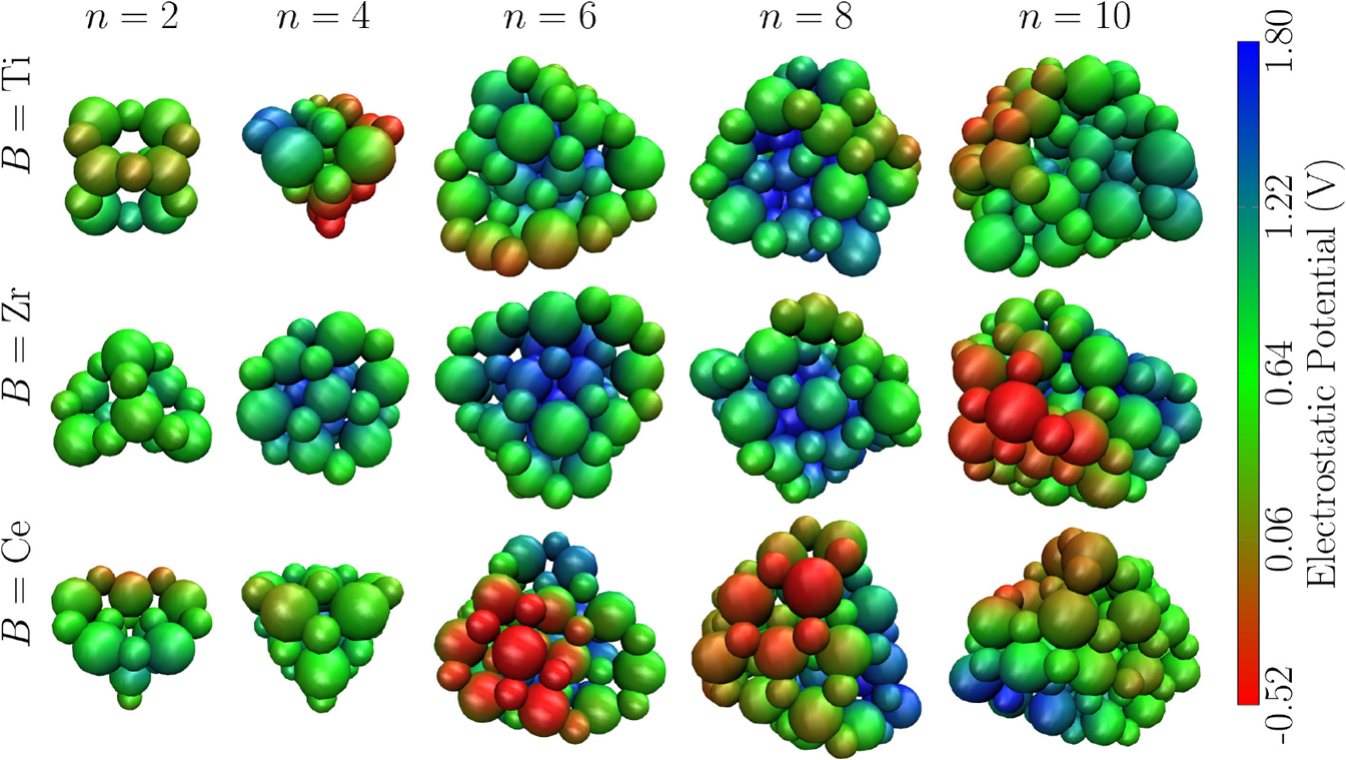
Electrostatic potential map obtained for (La_2_ *B*_2_O_7_)_*n*_ nanoclusters with *B* = Ti, Zr and Ce and *n* = 2, 4, 6, 8, and 10. The color line represents the potential value in V.

With an increase in the size of the nanocluster and the formation of core regions in their structures (*n* ≥ 6), a high and positive electrostatic potential (1.5 V) can be observed in the core, where highly coordinated atoms are present, as shown in Figure 7. These results help explain the stability of the larger nanoclusters observed in Figure 5. In contrast, the surface regions tend to exhibit islands of low electrostatic potential, suggesting lower stability in these regions and a propensity for defect formation. This effect can be associated with the difference in the charge distribution between more coordinated and less coordinated atoms present on the surface, which affects the balance of electrostatic forces, especially in reducible materials such as Ce and Ti.

### 3.3 Characterization of Oxygen Vacancy Formation in Lowest Energy Structures

In the following, we will focus on the formation of oxygen vacancies within the nanoclusters, in particular, in the lowest-energy structures, given their high stability and probability of occurrence, providing reliable physical properties.

#### 3.3.1 Selection of the Oxygen Vacancies

Once the energetic, electronic, and structural properties of the most stable structures within the (La_2_ *B*_2_O_7_)_*n*_ nanocluster set are understood, we aim to investigate how the formation of vacancies affects these properties. Thus, we selected the lowest-energy structures to study the formation of oxygen vacancies. The choice of an appropriate O site for the formation of vacancies can be a challenging task due to the low symmetry of the nanoclusters, resulting in many possible O sites surrounded by different chemical environments. To address this problem, we combined two internal Python algorithms with the aim of selecting the O sites with a wide range of chemical environments. The procedure, depicted in Figure 10, was carried out as follows:

1. *Geometric Analysis of Atomic Positions*: Using a Python script, we computed the Euclidean distance of every O atom to the geometric center. Thus, O atoms were classified based on their radial distance from the geometric center, segmented into bins of 0.5 Å. This discretization allows spatial partitioning of the nanocluster into core, subshell, as shown in Figure 10a, and surface-like regions, facilitating the sampling of chemically unique sites throughout the volume of the particles.
2. *Chemical Environment Classification*: In a second Python script, we parsed the output of VASP (OUTCAR file) to identify the local chemical environment of each O atom, in particular the identity and number of nearest neighbors (La, B), exemplified in Figure 10b.
3. *Selection of Unique Vacancy Sites*: This information was tabulated into a data structure containing O atom indices, neighbor identity, coordination number, and radial distance. Thus, within each distance bin, we selected one representative oxygen atom per unique chemical environment to form a vacancy.

Therefore, this systematic approach ensures that the oxygen vacancies generated cover a broad range of chemical and spatial environments in the nanoclusters. Such a strategy is especially relevant for low-symmetry, finite-size systems such as nanoclusters, where the energetic impact of a vacancy can be highly sensitive to the local atomic surroundings. All generated structures were reoptimized using the DFT framework.

**Figure 10:**
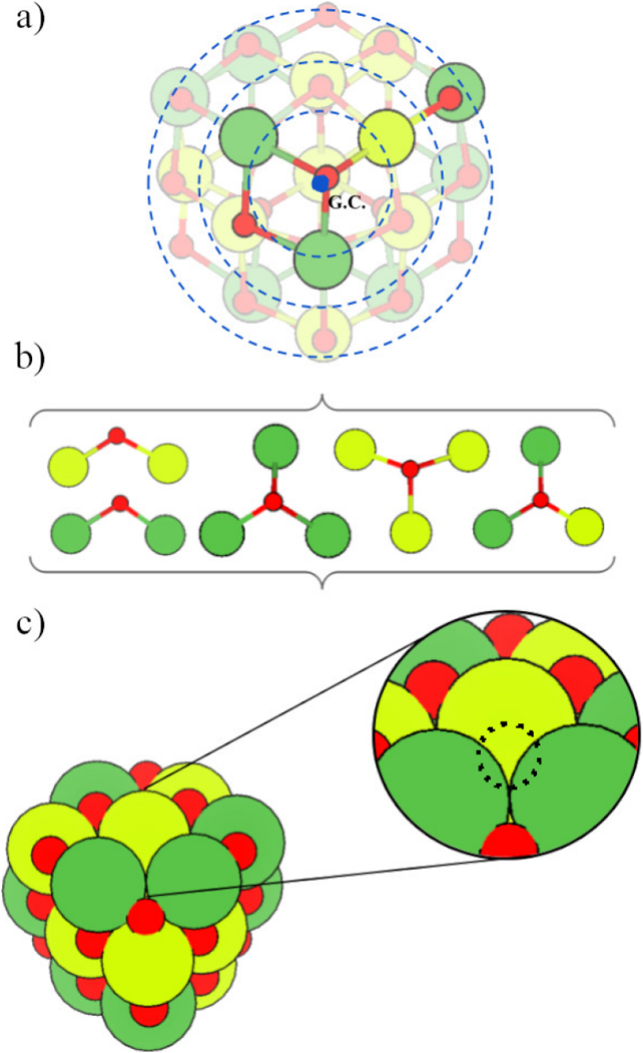
Schematic representation of the procedure adopted to generate oxygen vacancies in the (La_2_ *B*_2_O_7_)_*n*_ nanoclusters: (a) radial shells centered at the geometric center; (b) selection of oxygen atoms from distinct chemical environments; (c) generation of defective configurations via selective removal of unique oxygen atoms.

#### 3.3.2 Oxygen Vacancy Energy Formation Versus Distance to the Geometric Center

To improve our atom-level understanding of the formation of oxygen vacancies within mixed oxide nanoclusters, we calculated the formation of the oxygen vacancy energy (*E*_vac_) with respect to the O_2_ molecule for different oxygen sites. *E*_vac_ is calculated using the following equation:

$$E_{\mathrm{vac}}=E_{\mathrm{tot}}^{{\mathrm{La}}_{2n}B_{2n}O_{7n-1}}+\frac{1}{2}E_{\mathrm{tot}}^{O_{2}}-E_{\mathrm{tot}}^{{\mathrm{La}}_{2n}B_{2n}O_{7n}}$$ 8

where *E*_tot_^La_2*n*_B_2*n*_O_7*n*–1_^, *E*_tot_^O_2_^, and *E*_tot_^La_2*n*_B_2*n*_ *O*_7*n*_^ indicate the total energy of nanoclusters with an oxygen vacancy, gas-phase O_2_ molecule, and nanocluster, respectively. This formulation encapsulates the energy to create the oxygen vacancy and the formation of a O_2_ molecule in the gas phase, which mimics an experimental environment.^3^ The results are shown in Figure 11, which indicates a dependence of *E*_vac_ on the chemical identity of the *B*-cation (Ti, Zr, and Ce), as well as on the spatial position of the oxygen vacancy relative to the geometric center of the nanocluster (*d*_GC_).

**Figure 11:**
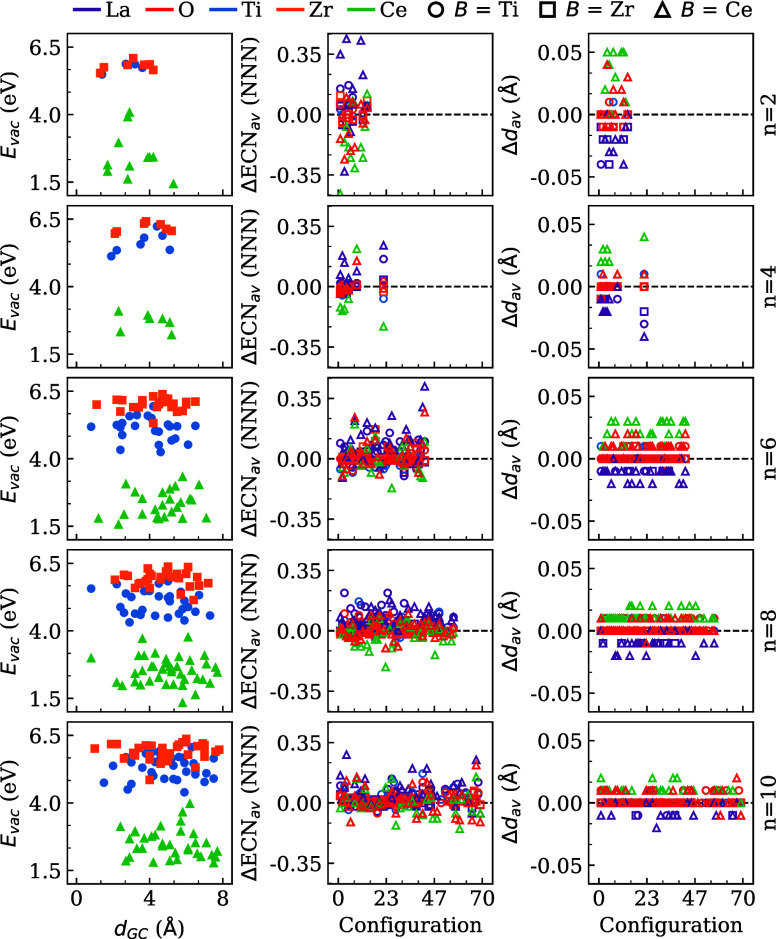
Vacancy energy, *E*_vac_, versus the initial position of the oxygen vacancy with respect to the geometric center of the nanocluster, *d*_GC_. Variation of effective coordination number (ΔECN), resulted from the vacancy formation. Variation of Average bond distance (Δ*d*_av_), resulting from the vacancy formation.

As expected, more reducible compounds are inclined to demonstrate lower vacancy formation energies. This phenomenon can be ascribed to the capacity of the cations to accommodate electrons after oxygen removal, particularly by means of their partially filled or low-lying *d*- or *f*-orbitals.^61^ For example, within the systems analyzed, the Ce-based nanoclusters consistently exhibit the lowest *E*_vac_, followed by the Ti- and Zr-based nanoclusters. This pattern signifies the electronic adaptability of Ce, attributable to the presence of available *f*-states that effectively localize the excess electrons, thus making vacancy formation energetically favorable.^62^

##### 3.3.2.1 Ce-Based Nanoclusters

Ce-containing nanoclusters display significant variability in the *E*_vac_ values, ranging from approximately 1 to 4 eV. A notable trend is observed: vacancies located in larger *d*_GC_, specifically near the nanocluster surface, demonstrate greater stability, as indicated by a lower *E*_vac_. This observation suggests that the surface regions of the nanocluster exhibit greater reducibility and possess an enhanced ability to stabilize oxygen vacancies, which aligns with the trends of defect formation discussed in Figure 9. In addition, this observation also aligns with the established understanding that surface oxygen atoms in reducible oxides exhibit higher stability. This phenomenon becomes increasingly evident as the nanocluster size enlarges, reflecting a pronounced contrast between the reducibility of surface and bulk regions.

##### 3.3.2.2 Ti-Based Nanoclusters

Ti-based nanoclusters display intermediate vacancy formation energies (approximately 4–6 eV) with a moderate dependence on *d*_GC_. For larger nanoclusters (e.g., *n* = 8 and 10), there is a slight but discernible decrease in *E*_vac_ with increasing *d*_GC_, pointing to a modest preference for the formation of vacancies near the surface of the nanocluster. This trend is consistent with the limited capacity of Ti to localize electrons in its 3*d* states, which are more delocalized and higher in energy than the Ce 4*f* levels.

##### 3.3.2.3 Zr-based Nanoclusters

In contrast, zirconium-based nanoclusters exhibit the highest vacancy formation energies, typically within the range of 5.5 to 6.5 eV, with minimal dependence on the position of the vacancy. This suggests a low intrinsic reducibility and a more rigid electronic structure that resists the localization of vacancy-induced electrons. The pronounced stability of zirconium–oxygen bonds and the limited involvement of Zr 4*d*-states in electron localization probably contribute to this phenomenon. As the size of the nanocluster increases from *n* = 2 to *n* = 10, the systems exhibit an increased number of potential vacancy sites and a more continuous distribution of *d*_GC_. However, observed chemical trends remain consistent between different sizes, indicating that the reducibility is primarily determined by the nature of the *B*-site element modulated by geometric and surface effects.

#### 3.3.3 Structural Changes due the Oxygen Vacancy Formation

In order to identify the structural changes induced on the nanocluster by the formation of oxygen vacancies, we calculated the changes in the effective coordination number (ΔECN_av_) and average bond distance (Δ*d*_av_), for all (La_2_ *B*_2_O_7_)_*n*_ studied configurations, using the following relations (i) ΔECN_av_ = ECN_av_^vac^ – ECN_av_ and (ii) Δ*d*_av_ = *d*_av_^vac^ – *d*_av_, where vac indicates nanoclusters with oxygen vacancies. The results are listed in Figure 11.

As expected, vacancy formation tends to lead to structural distortions in the nanoclusters. Such effects are more pronounced in smaller nanoclusters because the low coordination of the atoms makes them more sensitive to deformations. For these structures, we observed an increase in ECN_av_ for the La species, followed by a decrease in this property for the *B* species. As a consequence, an opposite behavior is observed for *d*_av_, which tends to decrease for La species and increase for *B* species. This behavior is observed on a smaller scale for nanoclusters with *n* ≥ 6, since the formation of the core indicates stability in the nanocluster, as discussed in Figure 5, suppressing the atomic rearrangement.

The aforementioned structural variations differ in relation to their origin for each type of chemical composition.^3^ For example, in a nanocluster based on Ce, the excess electrons from the removal specie O are responsible for the formation of a small polaron and the consequent reduction of two Ce atoms from Ce^4+^ to Ce^3+^, increasing its atomic radius. Then, a positive electrostatic potential is induced in the vacancy region, repelling the cationic neighboring species.^63^ The combination of both effects can be responsible for the reordering of the cationic species and the higher dispersions in the values ΔECN_av_ and Δ*d*_av_.

Ti-based nanoclusters presented lower structural variations compared with the Ce-based nanoclusters. In this materials, a vacancy formation can lead to two different effects: (*i*) Electronic delocalization, where electrons originating from vacancies can behave as free carriers, without reducing Ti species and conserving the atomic radius of Ti^4+^ atoms; (*ii*) Reduction of Ti^4+^ atoms to Ti^3+^, forming small polaron, which combined to the electrostatic potential are responsible for distortions in the lattice, observed in our results.^64^

In contrast to Ce and Ti, the material based on Zr showed the lowest dispersion in ΔECN_av_ and Δ*d*_av_. In these materials, the electrons remaining in the absence of O^2–^ tend to remain localized at defective sites, due to the irreducibility of Ce^4+^.^65^ The low structural variations observed here are in accordance with the high *E*_vac_ depicted in Figure 11, denoting the resistance of this material to the removal of oxygen.

#### 3.3.4 Electron Localization Effects due to the Oxygen Vacancy Formation

With the aim of understanding the magnetic polarization resulting from oxygen vacancies in the nanoclusters, we performed a spin density difference analysis using the VASPkit^66^ code, in which the spin density difference is defined as follows:

$$\rho {\left(r\right)}^{s}=\rho {\left(r\right)}^{\alpha }-\rho {\left(r\right)}^{\beta }$$ 9

where ρ­(**r**)^α^ and ρ­(**r**)^β^ represent the spin-up and spin-down electron densities, respectively. Figure 12 shows the isosurfaces of spin densities obtained by eq 9. The electronic and structural properties of the same nanocluster structures are presented in Table 2, where *E*_g_ is the difference in energy between the HOMO and LUMO states, considering the two spin components.

**Figure 12:**
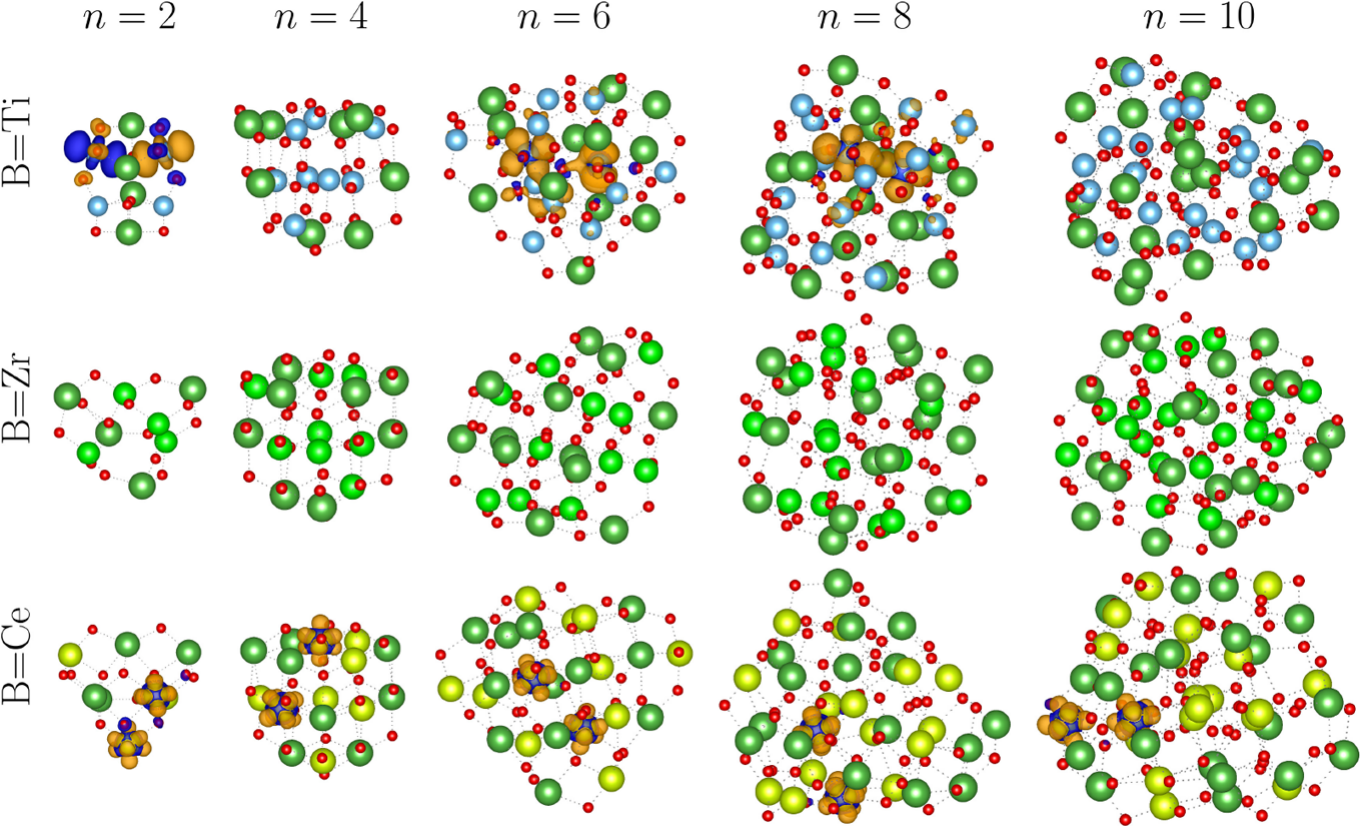
Isosurfaces visualization of spin density difference calculated for (La_2_ *B*_2_O_7_)_*n*_ nanoclusters, where *B* = Ti, Zr, and Ce and *n* = 2, 4, 6, 8, and 10. Orange regions represent spin up polarization, while blue regions represent spin down. The bonds between atoms were represented by dotted lines for better visualization of isosurfaces (0.01 Å^–3^).

**Table 2:** Energetic and Electronic Properties of (La_2_ *B*_2_O_7_)_*n*_ Nanoclusters with One Oxygen Vacancy^a^

*B*	*n*	*d*_GC_ (Å)	*E*_vac_ (eV)	*E*_g_ (eV)	*m*_tot_ (μ_B_)
Ti	2	1.40	5.48	1.68	0
	4	1.90	5.13	1.25	0
	6	4.60	4.25	1.13	2
	8	2.90	4.31	1.02	2
	10	5.90	4.39	0.79	0
Zr	2	1.30	5.54	1.83	0
	4	2.10	5.97	1.68	0
	6	4.20	5.32	1.12	0
	8	6.40	5.14	0.69	0
	10	4.00	4.86	0.62	0
Ce	2	5.30	1.43	1.57	2
	4	5.20	2.21	1.66	2
	6	2.30	1.56	1.18	2
	8	5.80	1.33	1.23	2
	10	7.50	1.77	0.96	2

a Vacancy energy formation, *E*_vac_, fundamental electronic band gap, *E*_g_, and magnetic moment, *m*_tot_.

Our results indicate that the effects on the electronic structure of an oxide due to the formation of vacancies strongly depend on the nature of the cationic species *B*. In materials based on Ce, the two excess electrons from vacancy formation tend to occupy the *f*-states of the two closest Ce species, changing their oxidation state from Ce^4+^ to Ce^3+^, resulting in the presence of a magnetic moment, as seen in Table 2.^61^ Then, the reduced species tend to occupy regions near vacancy, as an example of (La_2_Ce_2_B_7_)_6_, once the finite nature of a nanocluster allows the accommodation of Ce^3+^ in the structure. As the size of the nanocluster increases, we can observe a severe reduction in *E*_g_ and a slight decrease in the stability of the vacancy formation, indicating reactivity in higher structures.

The dual behavior of the electronic structure in materials based on Ti has been shown to be influenced by the size and symmetry of the structure. In this context, smaller configurations such as *n* = 2 and 6 indicate the reduction of Ti atoms, driven by the formation of vacancies that lead to a magnetic moment of zero or two, depending on whether the spins are opposite (*n* = 2) or aligned (*n* = 6). In highly symmetric configurations, such as *n* = 4 or larger clusters (*n* = 8 and 10), the atomic *B* species do not exhibit reducibility, resulting in no magnetic moment.

As in Ce-based nanoclusters, the energy gap of the Ti nanocluster decreases as the size of the nanocluster increases, following the behavior of the stoichiometric structures. The vacancy energy tends to decrease with the nanocluster size while remaining significantly higher relative to that of ceria systems. This behavior confirms that the reducibility is an important factor for the stability of vacancies. As expected, the Zr-based nanoclusters showed irreducible behavior. Electronic delocalization of the electrons does not reduce any Zr atom that retains the magnetic moment equal to zero, as seen in Table 2. As a consequence, the vacancy energies are higher, reinforcing the resistance to the formation of vacancies in these species.

## 4 Conclusions

We employed density functional theory calculations, incorporating Hubbard *U* and van der Waals D3 corrections, to elucidate the influence of nanocluster size and cation chemistry on the structural, energetic, electronic, and defect characteristics of mixed (La_2_ *B*_2_O_7_)_*n*_ nanoclusters (*B* = Ti, Zr, Ce; *n* = 2, 4, 6, 8, 10). Through systematic generation and optimization of structural models, coupled with extensive sampling of vacancy sites, we present a unified atomistic view that connects size-dependent morphology (such as core–shell formation and coordination environments), binding energies, electronic band gaps, electrostatic potential profiles, and the energetics of oxygen-vacancy formation with the underlying size effects and cationic chemistry.

The size of the nanocluster operates as the main determinant of structural motifs: for the smallest clusters (*n* = 2, 4), all atoms are predominantly exposed to the surface and exhibit a broad spectrum of low coordination geometries, while nanoclusters with *n* ≥ 6 form distinctly well-defined bulklike core regions. This morphological transition accounts for the narrowing of the energy distributions as *n* increases. Enhancements in coordination and 3D packing progressively constrain the configurational freedom. Throughout various sizes, there is a discernible inclination for La atoms to segregate to the outermost shells in the nanoclusters, whereas smaller *B* cations show a preference for cation sites in the core regions. This segregation is explained by considerations of size and coordination preferences and is corroborated by radial-distribution and RMSD analyses, which reveal more pronounced structural perturbations when Ti is substituted with the larger Zr or Ce in small clusters.

The average binding energy per atom decreases with the expansion of the cluster size, indicative of a decrease in the surface stress and the energetic benefits conferred by interior coordination. Clusters based on Zr demonstrate the strongest binding, attributable to potent Zr–O interactions and an elevated lattice energy. The unusual local stabilization observed in certain *n* = 4 structures is attributed to the advantageous local packing and the elevated effective coordination numbers within those specific isomers. Mapping of electrostatic potentials further elucidates stability trends: the cores of larger clusters exhibit highly positive potentials, which provide stabilization for interior, highly coordinated atoms, while surface regions reveal low-potential “islands” that reduce the energy cost associated with the formation of defects and focus reactivity at the surface.

The fundamental HOMO–LUMO energy gaps diminish as nanocluster size increases, which is attributed to the relaxation of quantum-confinement effects. However, the transition toward bulk-like gaps is not monotonic; anomalies in the gaps of Ti- and Zr-based clusters are observed at intermediate sizes, attributable to structural rearrangements and local coordination alterations that reposition the frontier orbitals. Trends in the average bond distance and effective coordination number support this observation: Ce-based clusters exhibit systematically extended bonds and slightly reduced coordination, consistent with their larger ionic radii and a more adaptable bonding network, which influences orbital hybridization and the energetic positioning of states proximate to the gap.

The formation energies of oxygen vacancies follow a distinct hierarchy La_2_Ce_2_O_7_ < La_2_Ti_2_O_7_ < La_2_Zr_2_O_7_, indicative of increasing reducibility from Zr to Ti to Ce and aligned with the relative ease of charge accommodation: Ce clusters exhibit the lowest formation energies *E*_vac_ (≈1 up to 4 eV across sites) with a pronounced surface preference; Ti nanoclusters present intermediate values (≈4 up to 6 eV), potentially demonstrating either electron delocalization or small-polaron formation, whereas Zr nanoclusters exhibit resistance to reduction (≈5.5 up to 6.5 eV) and largely remain nonreducible. The formation of vacancies induces characteristic structural changes, such as variations in ECN and average bond lengths, particularly pronounced in small clusters and Ce systems, where the reduction ${\mathrm{Ce}}^{4+}\to {\mathrm{Ce}}^{3+}$ results in an increase in the ionic radius and local repulsion, thus reorganizing adjacent cations. Patterns of electron localization and spin-density analyses reveal vacancy-induced magnetic moments (up to ∼2.0 μ_B_) in Ce systems and select Ti configurations, while Zr systems remain nonmagnetic, a behavior that directly links electronic/redox adaptability to potential catalytic function.

## Supplementary Material



## Data Availability

The authors declare no competing financial interest. As mentioned, all DFT calculations were done using the VASP package, which can be used under a nonfree academic license. Furthermore, additional details are provided within the electronic Supporting Information, while additional crude data can be obtained directly from the authors on request.

## Notes

Data used for the figures, as well as complementary analyses and additional technical details (PDF)

## Abbreviations

DFT: density functional theory
PBE: Perdew–Burke-Ernzerhof
VASP: Vienna ab initio simulation package
PAW: projected augmented-wave
ECN: effective coordination number
