# Supplementary material for: Computational Investigation of the Size Evolution of (La2 B 2O7) n Nanoclusters (B = Ce, Ti, Zr)
Source: ACS Omega. 2025 Oct 10;10(41):48829–43. doi: 10.1021/acsomega.5c06927 (PMC12547521; doi:10.1021/acsomega.5c06927)
Supplement: Supplementary file 1 [file ao5c06927_si_001.pdf]

Electronic Supporting Information File: Computational Investigation of the Size-Evolution of $(La_2B_2O_7)_n$ Nanoclusters (B = Ce, Ti, Zr)

Carina S. T. Peraça, Mauricio Mocelim, Mylena N. Santos, and Juarez L. F. Da Silva*

São Carlos Institute of Chemistry, University of São Paulo, Av. Trabalhador São-Carlense 400, 13560-970*, São Carlos, SP, Brazil*

E-mail: juarez_dasilva@iqsc.usp.br

Contents

S1 Introduction **S3**

S2 Additional Theoretical Approach and Computational Details **S3**

S2.1 Selected PAW Projectors S3

S2.2 Box Size Definition S3

S2.3 Bulk Convergence Calculation S5

S2.4 Oxygen Vacancy Site S6

S3 Additional Results for Non-Defective Nanoclusters **S16**

S3.1 Root Mean Square Deviation S17

S3.2 Radial Distribution Function S23

S3.3 Average Bond Length Distance and Effective Coordination Number of La, *B* and O Species S25

S3.4 Additional Features of Defect-Free Structures S26

S4 Additional Results for Lowest Energy Nanoclusters Configuration **S41**

S4.1 Binding Energy S42

S4.2 Electrostatic Potential Mapping S42

S5 Additional Effects of O-Vacancy Formation **S43**

S5.1 Additional Features for Defective Structures S47

References **S59**

S1 INTRODUCTION

This Electronic Supporting Information file contains all computational details relevant for data reproduction. We also provided additional data and analysis that supported our discussions and conclusions. Additional material is available upon request from the authors.

S2 ADDITIONAL THEORETICAL APPROACH AND COMPUTATIONAL DETAILS

S2.1 Selected PAW Projectors

All calculations in this project are performed via density functional theory (DFT) as implemented in Vienna *Ab initio* Simulation Package (VASP) software. In VASP, the Kohn–Sham (KS) equations are solved using the Frozen-Core Approximation (PAW) method to describe the interactions between core and valence electrons, with KS orbitals expanded in a plane-wave basis set. In this study, we selected the most recent PAW projectors for each chemical species, providing an accurate description of its valence states, as shown in Table S1.

Table S1. Computational technical details for the selected PAW-PBE projectors. Recommended maximum cutoff energy for the plane wave basis set, `ENMAX`, number of valence electrons, Z_{val}, and valence electronic configuration.

Element	PAW Projector	Date	Z_{val}	Valence	`ENMAX` (eV)
Ce	Ce_GW	03/26/2009	12	$6S^2 5s^2 5p^6 5d^1 4f^1$	304.625
La	La_GW	05/16/2012	11	$5s^2 5p^6 6s^2 5d^1$	313.688
O	O_GW_new	03/19/2012	6	$2s^2 2p^4$	434.431
Ti	Ti_sv_GW_new	12/05/2013	12	$3s^2 3p^6 3d^4$	383.774
Zr	Zr_sv_GW_new	12/05/2013	12	$4s^2 4p^6 4d^4$	346.364

S2.2 Box Size Definition

We defined the nanocluster box size (a) based on the following criteria:

$$a = 2R_c + 15\,\text{Å}, \tag{S1}$$

where R_c is the nanocluster radius. Table S2, contain the R_c and a of a representative configuration of $(La_2B_2O_7)_n$ nanocluster with B = Ce, Ti, Zr and $n = 2, 4, 6, 8, 10$. The criteria for choosing the representative configuration was the largest R_c value. We choose the same box size for different species of B according to the respective value of n. Then, the box dimensions were defined as 25.20, 28.48, 30.12, 32.08, and 34.36 Å, for $n = 2, 4, 6, 8, 10$, respectively.

Table S2. Box size parameters obtained for representative $(La_2B_2O_7)_n$ nanoclusters, where n is the number of units and B the metallic center. The parameters studied are the radius of the nanocluster R_c and the size of the nanocluster box a, both properties are described in Å.

Ti			Zr			Ce		
n	R_c	a	n	R_c	a	n	R_c	a
2	4.84	24.68	2	5.10	25.20	2	4.88	24.76
4	6.38	27.76	4	6.67	28.34	4	6.74	28.48
6	7.31	29.62	6	7.49	29.98	6	7.56	30.12
8	8.53	32.06	8	8.45	31.90	8	8.54	32.08
10	8.54	32.08	10	9.01	33.02	10	9.68	34.36

S2.3 Bulk Convergence Calculation

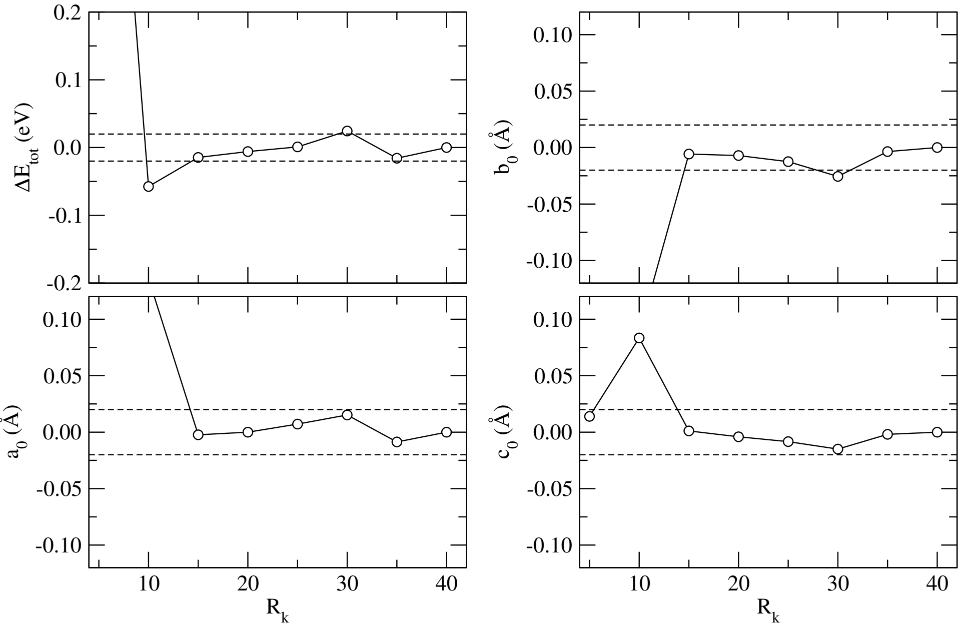

Figure S1. Computational convergence of the equilibrium lattice constant (a_0, b_0 and c_0) and relative total energy (ΔE_{tot}) as a function of the number of $\mathbf{k}$-points in the irreducible Brillouin Zone ($R_{\mathbf{k}}$) for the structure of $La_2Ti_2O_7$ in bulk phase. The dotted line indicates the acceptable margin of error for convergence.

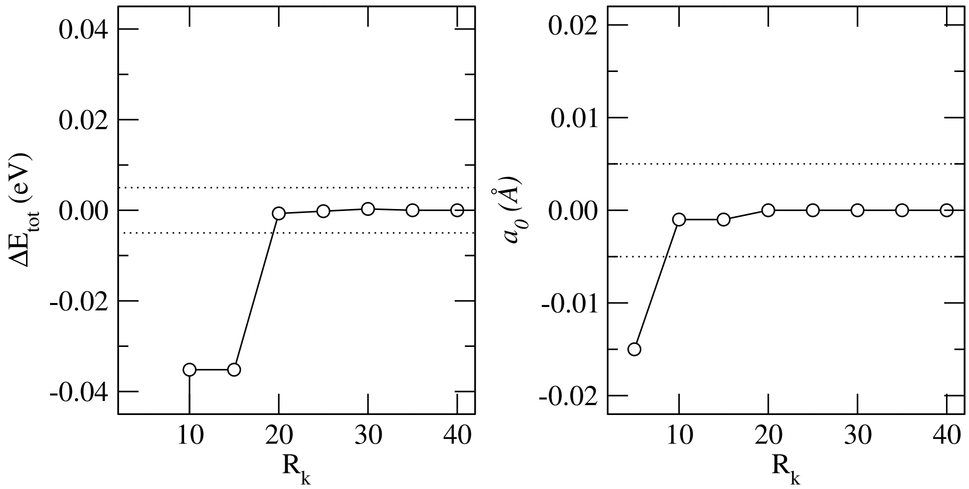

Figure S2. Computational convergence of the equilibrium lattice constant (a_0) and relative total energy (ΔE_{tot}) as a function of the number of $\mathbf{k}$-points in the irreducible Brillouin Zone ($R_{\mathbf{k}}$) for the structure of $La_2Zr_2O_7$ in bulk phase. The dotted line indicates the acceptable error for convergence.

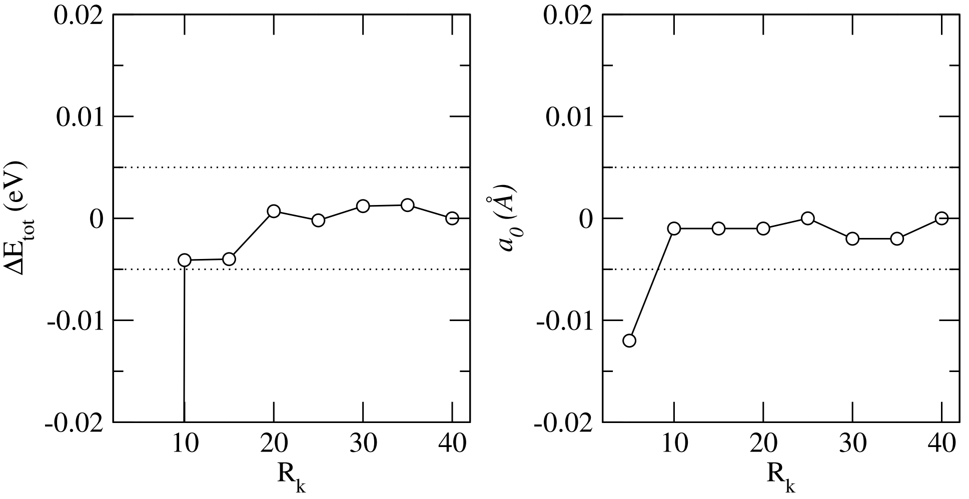

Figure S3. Computational convergence of the equilibrium lattice constant (a_0) and relative total energy (ΔE_{tot}) as a function of the number of **k**-points in the irreducible Brillouin Zone ($R_{\mathbf{k}}$) for the structure of $La_2Ce_2O_7$ in bulk phase. The dotted line indicates the acceptable error for convergence.

S2.4 Oxygen Vacancy Site

Table S3. Output from the in-house algorithm that compares distance from CG and chemical ambient for $(La_2Ti_2O_7)_2$. The first, second, third, and fourth columns represent the indices of atomic species, i, atomic species, the chemical environment and the distance from the respective specie to the CG, d^{CG}, in Å.

i	Species	Environment	d^{CG}
7	O	Ti Ti	1.4
14	O	Ti Ti	1.4
2	O	Ti Ti	2.7
3	O	Ti Ti	2.7
1	O	La La	3.2
4	O	La La	3.2
5	O	Ti La	3.6
6	O	Ti La	3.6
8	O	Ti La	3.6
9	O	Ti La	3.6
10	O	Ti La	3.6
11	O	Ti La	3.6
12	O	Ti La	3.6
13	O	Ti La	3.6

Table S4. Output from the in-house algorithm that compares distance from CG and chemical ambient for $(La_2Ti_2O_7)_4$. The first, second, third, and fourth columns represent the indices of atomic species, i, atomic species, the chemical environment and the distance from the respective specie to the CG, d^{CG}, in Å.

i	Species	Environment	d^{CG}
5	O	Ti Ti Ti La	1.9
8	O	Ti Ti Ti La	1.9
24	O	Ti Ti Ti La	1.9
27	O	Ti Ti Ti La	1.9
9	O	Ti Ti La La	2.2
14	O	Ti Ti La La	2.2
4	O	Ti Ti La	3.5
11	O	Ti Ti La	3.5
17	O	Ti Ti La	3.5
23	O	Ti Ti La	3.5
7	O	Ti Ti La	3.6
10	O	Ti Ti La	3.6
13	O	Ti Ti La	3.6
19	O	Ti Ti La	3.6
2	O	Ti La La	3.7
6	O	Ti La La	3.7
16	O	Ti La La	3.7
20	O	Ti La La	3.7
3	O	Ti La La	4.4
15	O	Ti La La	4.4
21	O	Ti La La	4.4
28	O	Ti La La	4.4
1	O	Ti La	4.7
12	O	Ti La	4.7
18	O	Ti La	4.7
25	O	Ti La	4.7
22	O	La La	5.1
26	O	La La	5.1

Table S5. Output from the in-house algorithm that compares distance from CG and chemical ambient for $(La_2Ti_2O_7)_6$. Each column presents the index (i), atomic species, chemical environment (Env.), and distance from the CG, d^{CG} (in Å).

i	Species	Env.	d^{CG}	i	Species	Env.	d^{CG}	i	Species	Env.	d^{CG}
8	O	Ti Ti Ti La	0.8	5	O	Ti Ti La	3.4	23	O	La La La	4.5
42	O	Ti La La La	2.2	9	O	Ti Ti La	3.4	26	O	La La La	4.5
24	O	Ti Ti Ti	2.4	18	O	Ti Ti La	3.9	36	O	Ti Ti La	4.5
40	O	Ti Ti La	2.4	34	O	Ti Ti La La	4.1	1	O	Ti La La	4.6
3	O	Ti Ti La La	2.5	27	O	Ti La La	4.2	13	O	Ti La La	4.6
38	O	Ti Ti	2.5	32	O	Ti Ti	4.3	28	O	Ti La La	4.8
19	O	Ti Ti La	2.9	16	O	Ti La La	4.4	39	O	Ti La	5.0
35	O	Ti Ti La La	2.9	21	O	Ti Ti La	4.4				
31	O	Ti Ti Ti La	3.2	33	O	Ti La La	4.4				
37	O	Ti Ti La	3.3	7	O	Ti Ti La	4.5				

Table S6. Output from the in-house algorithm that compares distance from CG and chemical ambient for $(La_2Ti_2O_7)_8$. Each column presents the index (i), atomic species, chemical environment (Env.), and distance from the CG, d^{CG} (in Å).

i	Species	Env.	d^{CG}	i	Species	Env.	d^{CG}	i	Species	Env.	d^{CG}
8	O	Ti Ti Ti La	0.8	27	O	Ti La La	4.2	12	O	Ti Ti	5.2
42	O	Ti La La La	2.2	32	O	Ti Ti	4.3	41	O	La La La	5.2
24	O	Ti Ti Ti	2.4	16	O	Ti La La	4.4	6	O	Ti La	5.3
40	O	Ti Ti La	2.4	21	O	Ti Ti La	4.4	2	O	Ti Ti	5.4
3	O	Ti Ti La La	2.5	33	O	Ti La La	4.4	17	O	Ti La	5.4
38	O	Ti Ti	2.5	7	O	Ti Ti La	4.5	25	O	Ti La La	5.4
19	O	Ti Ti La	2.9	23	O	La La La	4.5	15	O	Ti La	5.5
35	O	Ti Ti La La	2.9	26	O	La La La	4.5	20	O	Ti La	5.7
31	O	Ti Ti Ti La	3.2	36	O	Ti Ti La	4.5	11	O	Ti La	5.9
37	O	Ti Ti La	3.3	1	O	Ti La La	4.6	22	O	Ti La	6.1
5	O	Ti Ti La	3.4	13	O	Ti La La	4.6	4	O	Ti La	6.2
9	O	Ti Ti La	3.4	28	O	Ti La La	4.8	29	O	Ti La	6.4
18	O	Ti Ti La	3.9	39	O	Ti La	5.0	30	O	Ti La	6.5
34	O	Ti Ti La La	4.1	14	O	La La La	5.1				

Table S7. Output from the in-house algorithm that compares distance from CG and chemical ambient for $(La_2Ti_2O_7)_{10}$. Each row presents the index (i), atomic species, chemical environment (Env.), and distance from the CG, d^{CG} (in Å).

i	Species	Env.	d^{CG}	i	Species	Env.	d^{CG}	i	Species	Env.	d^{CG}
56	O	Ti Ti La	1.5	17	O	Ti La La	4.9	47	O	Ti La	6.4
20	O	Ti Ti La	2.0	42	O	Ti Ti La	4.9	48	O	Ti La La	6.4
18	O	Ti Ti La	2.2	54	O	Ti La La	4.9	51	O	Ti La	6.4
23	O	Ti Ti La	2.7	3	O	Ti La La	5.2	64	O	Ti La La	6.4
41	O	Ti Ti	2.8	43	O	Ti Ti La	5.2	1	O	La La La	6.5
12	O	Ti Ti La La	3.0	7	O	Ti La	5.3	35	O	La La La	6.5
45	O	Ti Ti La	3.0	60	O	Ti Ti La La	5.3	58	O	Ti La La	6.5
34	O	Ti Ti La	3.4	65	O	Ti Ti La	5.3	62	O	Ti La	6.5
25	O	Ti Ti Ti	3.6	29	O	Ti Ti La	5.4	63	O	Ti La La	6.6
5	O	Ti Ti	3.8	28	O	Ti La	5.5	30	O	Ti La	6.8
55	O	Ti Ti La La	3.9	10	O	Ti La La	5.6	38	O	La La La	6.8
66	O	Ti Ti La La	3.9	31	O	Ti La La	5.6	46	O	Ti La	6.8
11	O	Ti La La	4.1	53	O	Ti La La	5.6	6	O	La La La	6.9
27	O	Ti Ti Ti	4.2	32	O	Ti Ti La	5.8	9	O	Ti La	6.9
39	O	Ti Ti La	4.2	49	O	Ti Ti La	5.8	37	O	Ti La	6.9
69	O	Ti Ti La	4.2	2	O	Ti Ti La	5.9	33	O	La La La	7.0
22	O	Ti La	4.3	19	O	Ti Ti	5.9	52	O	Ti La La	7.0
67	O	Ti La	4.3	36	O	Ti Ti La La	5.9	68	O	Ti La	7.1
8	O	Ti Ti	4.4	59	O	Ti La La	5.9	24	O	Ti La	7.5
15	O	Ti Ti La	4.4	26	O	Ti La La	6.0	50	O	Ti La La	7.5
13	O	Ti Ti La	4.6	61	O	Ti La La	6.0				
14	O	Ti Ti	4.6	4	O	Ti La La	6.2				
44	O	Ti La La	4.6	70	O	Ti La La	6.2				
57	O	Ti Ti La La	4.6	16	O	Ti Ti	6.3				
21	O	Ti Ti Ti	4.8	40	O	Ti La	6.4				

Table S8. Output from the in-house algorithm that compares distance from CG and chemical ambient for $(La_2Zr_2O_7)_2$. The first, second, third, and fourth columns represent the indices of atomic species,i, atomic species, the chemical environment and the distance from the respective specie to the CG, d^{CG}, in Å.

i	Specie	Environment	d^{CG}
7	O	Zr Zr	1.3
14	O	Zr Zr La	1.5
1	O	Zr La	2.8
11	O	Zr La	2.8
8	O	Zr Zr	2.9
12	O	Zr La	3.1
6	O	Zr La	3.4
10	O	Zr La	3.4
2	O	Zr La	3.7
9	O	Zr La	3.8
13	O	Zr La	3.9
4	O	Zr La	4.0
3	O	Zr La	4.1
5	O	La La	4.2

Table S9. Output from the in-house algorithm that compares distance from CG and chemical ambient for $(La_2Zr_2O_7)_4$. The first, second, third, and fourth columns represent the indices of atomic species,i, atomic species, the chemical environment and the distance from the respective specie to the CG, d^{CG}, in Å.

i	Specie	Env.	d^{CG}
5	O	Zr Zr Zr La	2.1
8	O	Zr Zr Zr La	2.1
24	O	Zr Zr Zr La	2.1
27	O	Zr Zr Zr La	2.1
9	O	Zr Zr La La	2.2
14	O	Zr Zr La La	2.2
4	O	Zr Zr La	3.7
7	O	Zr Zr La	3.7
10	O	Zr Zr La	3.7
11	O	Zr Zr La	3.7
13	O	Zr Zr La	3.7
17	O	Zr Zr La	3.7
19	O	Zr Zr La	3.7
23	O	Zr Zr La	3.7
2	O	Zr La La	3.8
6	O	Zr La La	3.8
16	O	Zr La La	3.8
20	O	Zr La La	3.8
3	O	Zr La La	4.6
15	O	Zr La La	4.6
21	O	Zr La La	4.6
28	O	Zr La La	4.6
1	O	Zr La	4.9
12	O	Zr La	4.9
18	O	Zr La	4.9
25	O	Zr La	4.9
22	O	La La	5.2
26	O	La La	5.2

Table S10. Output from the in-house algorithm that compares distance from CG and chemical ambient for $(La_2Zr_2O_7)_6$. Each subtable presents the index (i), atomic species, chemical environment (Env.) and distance from the CG, d^{CG} (in Å).

i	Species	Env.	d^{CG}	i	Species	Env.	d^{CG}	i	Species	Env.	d^{CG}
8	O	Zr Zr Zr La	1.1	33	O	Zr La La	4.3	41	O	La La La	5.4
42	O	Zr La La La	2.2	34	O	Zr Zr	4.4	12	O	Zr Zr	5.5
24	O	Zr Zr Zr	2.4	26	O	La La La	4.5	15	O	Zr La	5.8
38	O	Zr Zr	2.4	13	O	Zr La La	4.6	17	O	Zr La	5.8
3	O	Zr Zr La	2.5	16	O	Zr La La	4.6	20	O	Zr La	5.8
40	O	Zr Zr La	2.5	1	O	Zr La La	4.7	2	O	Zr Zr	6.0
35	O	Zr Zr La	3.1	21	O	Zr Zr La	4.7	11	O	Zr La	6.0
19	O	Zr Zr La	3.3	23	O	La La La	4.7	22	O	Zr La	6.3
31	O	Zr Zr Zr La	3.4	28	O	Zr La La	4.8	4	O	Zr La	6.5
5	O	Zr Zr La	3.6	32	O	Zr Zr	4.8	29	O	Zr La	6.6
9	O	Zr Zr La La	3.6	36	O	Zr Zr	5.0	30	O	Zr La	6.8
37	O	Zr Zr La	3.6	39	O	Zr La	5.0	10	O	Zr La	6.9
18	O	Zr Zr La	4.2	14	O	La La La	5.2				
27	O	Zr La La	4.2	25	O	Zr La La	5.3				
7	O	Zr La	4.3	6	O	Zr La	5.4				

Table S11. Output from the in-house algorithm that compares distance from CG and chemical ambient for $(La_2Zr_2O_7)_8$. Each subtable presents the index (i), atomic species, chemical environment (Env.) and distance from the CG, d^{CG} (in Å).

i	Species	Env.	d^{CG}	i	Species	Env.	d^{CG}	i	Species	Env.	d^{CG}
19	O	Zr Zr Zr	0.9	14	O	Zr Zr La La	4.1	1	O	Zr La La	5.5
43	O	Zr Zr La La	2.1	24	O	Zr Zr La	4.2	22	O	Zr La La	5.5
8	O	Zr Zr La	2.6	52	O	Zr Zr La La	4.2	36	O	Zr La La	5.5
7	O	Zr Zr La	2.7	9	O	Zr Zr La	4.6	39	O	Zr Zr La	5.6
10	O	Zr Zr Zr	2.8	31	O	Zr Zr	4.6	21	O	La La La	5.7
45	O	Zr Zr La	2.8	11	O	La La La	4.9	35	O	Zr La La	5.7
28	O	Zr Zr La	3.2	53	O	Zr Zr La	4.9	29	O	Zr Zr La	5.9
6	O	Zr Zr	3.4	2	O	Zr La	5.0	55	O	Zr La La	5.9
34	O	Zr Zr La	3.5	17	O	Zr Zr	5.0	12	O	Zr La La	6.0
30	O	Zr Zr La	3.8	38	O	Zr Zr La	5.0	15	O	Zr La	6.0
49	O	Zr La La La	3.8	23	O	Zr La La	5.2	16	O	Zr La La	6.1
40	O	Zr Zr La La	3.9	25	O	Zr Zr La	5.2	44	O	Zr La	6.1
48	O	Zr Zr Zr La	3.9	51	O	Zr La La	5.2	46	O	Zr Zr	6.1
13	O	Zr La La	4.0	42	O	Zr La La	5.4	20	O	Zr Zr	6.2
47	O	Zr Zr La	4.0	50	O	Zr La La	5.4	32	O	La La La	6.2

Table S12. Output from the in-house algorithm that compares distance from CG and chemical ambient for $(La_2Zr_2O_7)_8$. Each subtable presents the index (i), atomic species, chemical environment (Env.) and distance from the CG, d^{CG} (in Å).

i	Species	Env.	d^{CG}	i	Species	Env.	d^{CG}	i	Species	Env.	d^{CG}
66	O	Zr Zr Zr Zr	1.0	69	O	Zr Zr Zr	4.1	52	O	Zr La La	5.4
56	O	Zr Zr	1.9	63	O	Zr Zr La	4.2	18	O	La La La	5.5
45	O	Zr Zr Zr	2.2	34	O	Zr Zr La La	4.4	54	O	Zr La La	5.6
23	O	Zr Zr Zr	2.4	42	O	Zr Zr La	4.4	60	O	Zr La La	5.7
9	O	Zr Zr La	2.6	31	O	Zr Zr La La	4.6	27	O	Zr Zr La	5.8
41	O	Zr Zr La	3.1	21	O	Zr Zr La	4.7	47	O	Zr La La	5.9
44	O	Zr Zr Zr	3.2	36	O	Zr La La	4.7	49	O	Zr La La	5.9
12	O	Zr Zr Zr	3.3	39	O	Zr La La	4.7	58	O	La La La	5.9
15	O	Zr Zr La	3.4	22	O	Zr Zr La	5.0	59	O	Zr La La	5.9
67	O	Zr Zr La La	3.4	55	O	Zr Zr La	5.0	68	O	Zr La La	5.9
5	O	Zr Zr La	3.8	11	O	Zr Zr	5.1	10	O	Zr La La	6.0
20	O	Zr Zr La	3.8	30	O	Zr La La	5.1	16	O	Zr Zr La	6.0
4	O	Zr Zr La La	4.0	43	O	Zr La La	5.2	17	O	Zr La	6.0
13	O	Zr Zr La	4.0	2	O	Zr Zr La	5.3	40	O	Zr La La	6.0
29	O	Zr Zr Zr La	4.0	3	O	Zr Zr La	5.3	53	O	Zr Zr La	6.0

Table S13. Output from the in-house algorithm that compares distance from CG and chemical ambient for $(La_2Ce_2O_7)_2$. The first, second, third, and fourth columns represent the indices of atomic species,i, atomic species, the chemical environment and the distance from the respective specie to the CG, d^{CG}, in Å.

i	Specie	Environment	d^{CG}
7	O	Ce Ce La La	1.7
12	O	La Ce La La	1.7
11	O	Ce La La	2.3
3	O	Ce Ce Ce	2.8
4	O	Ce La La	2.8
13	O	Ce La	2.9
14	O	Ce Ce La	2.9
5	O	Ce La	3.9
1	O	La La	4.0
2	O	Ce La	4.0
9	O	Ce La	4.0
8	O	Ce La	4.2
10	O	Ce La	4.3
6	O	Ce	5.3

Table S14. Output from the in-house algorithm that compares distance from CG and chemical ambient for $(La_2Ce_2O_7)_4$. The first, second, third, and fourth columns represent the indices of atomic species,i, atomic species, the chemical environment and the distance from the respective specie to the CG, d^{CG}, in Å.

i	Specie	Environment	d^{CG}
5	O	Ce Ce Ce La	2.3
8	O	Ce Ce Ce La	2.3
24	O	Ce Ce Ce La	2.3
27	O	Ce Ce Ce La	2.3
9	O	Ce Ce La La	2.4
14	O	Ce Ce La La	2.4
4	O	Ce Ce La	3.9
7	O	Ce Ce La	3.9
10	O	Ce Ce La	3.9
11	O	Ce Ce La	3.9
13	O	Ce Ce La	3.9
17	O	Ce Ce La	3.9
19	O	Ce Ce La	3.9
23	O	Ce Ce La	3.9
2	O	Ce La La	4.0
6	O	Ce La La	4.0
16	O	Ce La La	4.0
20	O	Ce La La	4.0
3	O	Ce La La	4.7
15	O	Ce La La	4.7
21	O	Ce La La	4.7
28	O	Ce La La	4.7
1	O	Ce La	5.1
12	O	Ce La	5.1
18	O	Ce La	5.1
25	O	Ce La	5.1
22	O	La La	5.2
26	O	La La	5.2

Table S15. Output from the in-house algorithm that compares distance from CG and chemical ambient for $(La_2Ce_2O_7)_6$. Each subtable presents the index (i), atomic species, chemical environment (Env.) and distance from the CG, d^{CG} (in Å).

i	Species	Env.	d^{CG}	i	Species	Env.	d^{CG}	i	Species	Env.	d^{CG}
8	O	Ce Ce La Ce	1.2	42	O	Ce La La La	2.3	40	O	Ce Ce La	2.6
3	O	Ce Ce La La	2.7	24	O	Ce Ce La	2.7	38	O	Ce Ce La	2.8
35	O	Ce Ce La La	3.2	19	O	Ce La Ce	3.6	31	O	Ce Ce Ce	3.6
5	O	Ce Ce La	3.7	37	O	Ce Ce La	3.8	9	O	Ce Ce La	3.9
33	O	Ce La La	4.3	18	O	Ce Ce La La	4.4	27	O	Ce La La	4.4
26	O	La La La	4.5	23	O	La La La	4.6	16	O	Ce La La	4.7
34	O	Ce Ce La La	4.7	1	O	Ce La La	4.8	7	O	Ce Ce La	4.8
13	O	La Ce La	4.8	36	O	Ce Ce La	4.8	21	O	Ce Ce La	4.9
28	O	Ce La La	5.0	20	O	Ce Ce La	5.1	32	O	Ce Ce	5.1
14	O	La La La	5.2	39	O	Ce La	5.4	41	O	La La	5.5
25	O	Ce La La	5.7	6	O	Ce La	5.8	12	O	Ce Ce	5.8
15	O	Ce La	6.0	17	O	Ce La	6.0	2	O	Ce Ce	6.2
11	O	Ce La	6.3	4	O	Ce La	6.7	22	O	Ce La	6.7
29	O	Ce La	6.8	30	O	Ce La	7.1	10	O	Ce La	7.3

Table S16. Output from the in-house algorithm that compares distance from CG and chemical ambient for $(La_2Ce_2O_7)_8$. Each subtable presents the index (i), atomic species, chemical environment (Env.) and distance from the CG, d^{CG} (in Å).

i	Species	Env.	d^{CG}	i	Species	Env.	d^{CG}	i	Species	Env.	d^{CG}
7	O	Ce Ce La La	0.8	30	O	Ce Ce	2.2	20	O	Ce Ce La	2.5
35	O	Ce La Ce	3.2	49	O	Ce Ce Ce La	3.2	14	O	Ce La La	3.4
34	O	Ce Ce La La	3.5	8	O	La La Ce La	3.6	44	O	Ce La Ce La	3.6
27	O	Ce Ce La	3.7	53	O	Ce La Ce La	3.8	25	O	Ce La La	4.0
40	O	Ce Ce La La	4.1	12	O	Ce Ce La	4.2	18	O	Ce La La La	4.3
32	O	Ce Ce La	4.4	17	O	Ce Ce Ce	4.5	13	O	Ce Ce Ce	4.6
22	O	Ce La La	4.7	5	O	Ce Ce Ce	4.9	19	O	Ce La La La	4.9
45	O	Ce Ce La	4.9	54	O	Ce Ce La	4.9	46	O	Ce Ce La	5.0
52	O	Ce Ce	5.1	6	O	Ce Ce Ce	5.2	37	O	Ce La La	5.2
1	O	Ce Ce La	5.3	24	O	Ce La La	5.5	47	O	La La La	5.5
42	O	Ce Ce La	5.6	16	O	Ce La La	5.7	31	O	Ce La Ce	5.7
4	O	Ce Ce Ce	5.8	29	O	Ce La	5.8	33	O	Ce Ce	5.9
39	O	Ce La La	5.9	43	O	Ce La	6.0	11	O	Ce La La	6.1
36	O	Ce La La	6.1	2	O	Ce Ce La	6.2	21	O	Ce La La	6.2
48	O	Ce La La	6.2	41	O	Ce La	6.3	50	O	Ce La La	6.3
26	O	La La La	6.5	28	O	Ce Ce La	6.5	38	O	Ce La	6.5
51	O	Ce La La	6.6	3	O	Ce La	6.7	56	O	Ce La	6.9
15	O	La La	7.0	10	O	Ce Ce	7.1	9	O	Ce La La	7.3
55	O	Ce La	7.6	23	O	La La La	7.7				

Table S17. Output from the in-house algorithm that compares distance from CG and chemical ambient for $(La_2Ce_2O_7)_{10}$. Each subtable presents the index (i), atomic species, chemical environment (Env.) and distance from the CG, d^{CG} (in Å).

i	Species	Env.	d^{CG}	i	Species	Env.	d^{CG}	i	Species	Env.	d^{CG}
66	O	Ce Ce Ce Ce	0.7	67	O	Ce Ce La	3.6	55	O	Ce Ce La	5.5
45	O	Ce Ce Ce La	2.4	4	O	Ce La Ce La	3.7	46	O	Ce La	5.7
23	O	Ce Ce Ce	2.7	69	O	Ce Ce Ce	4.1	60	O	Ce La La	5.7
9	O	Ce Ce Ce Ce	2.9	13	O	Ce Ce La La	4.2	2	O	Ce Ce La	5.8
56	O	Ce Ce La	2.9	39	O	La La Ce Ce	4.3	27	O	Ce Ce La	5.8
12	O	Ce Ce Ce La	3.2	63	O	Ce Ce Ce La	4.3	47	O	Ce La La	5.9
20	O	Ce Ce Ce	3.2	61	O	Ce La Ce	4.5	7	O	Ce Ce La	6.0
41	O	Ce Ce La	3.3	5	O	Ce Ce La Ce	4.6	26	O	Ce Ce Ce	6.1
44	O	Ce Ce Ce	3.4	15	O	Ce Ce La Ce	4.6	59	O	Ce La La	6.1
29	O	Ce La Ce Ce	4.6	68	O	Ce La La	6.1	58	O	La La La	6.3
11	O	Ce La Ce La	4.7	17	O	Ce La La	6.2	16	O	Ce Ce	6.4
31	O	Ce La Ce	4.7	28	O	La La La	6.2	54	O	La Ce La	6.4
42	O	Ce La Ce	4.8	70	O	Ce La La	6.2	8	O	Ce La	6.5
22	O	Ce La Ce	5.0	14	O	Ce La La	6.3	10	O	Ce La La	6.5
34	O	Ce La Ce	5.1	40	O	Ce La La	6.3	50	O	Ce La La	6.5
36	O	La La La	5.1	52	O	Ce La La	6.3	30	O	Ce La La	6.6
53	O	Ce Ce La	5.1	3	O	Ce La	5.5	65	O	Ce La	6.8
18	O	La La La	5.4	25	O	Ce La	5.5	19	O	Ce La	6.9
21	O	Ce Ce La	5.4	43	O	Ce La La	5.4	33	O	Ce La	6.9
35	O	Ce La La	7.0	49	O	Ce La	6.9	48	O	Ce La	7.1

The number of structures generated for each nanocluster is available on Table S18.

Table S18. Number of calculated structures of $(La_2Zr_2O_7)_n$, $(La_2Ce_2O_7)_n$ and $(La_2Ti_2O_7)_n$ with a single O vacancy formation.

B/n	2	4	6	8	10
Ti	4	7	24	30	38
Zr	7	7	25	30	33
Ce	11	7	25	40	34

S3 ADDITIONAL RESULTS FOR NON-DEFECTIVE NANOCLUSTERS

This section contains additional results used in the analyses that gave rise to the graphs and figures presented in the body of the article.

S3.1 Root Mean Square Deviation

Table S19. RMSD value for comparison of $(La_2Ce_2O_7)_2$ and $(La_2Zr_2O_7)_2$ with $(La_2Ti_2O_7)_2$ and between each other, $RMSD^{Ti-Ce}$, $RMSD^{Ti-Zr}$ and $RMSD^{Zr-Ce}$, respectively, measured in Å.

config.	$RMSD^{Ti-Ce}$	$RMSD^{Ti-Zr}$	$RMSD^{Zr-Ce}$
1	0.404	0.307	0.442
2	0.445	0.256	0.324
3	0.378	0.419	0.516
4	0.383	0.189	0.209
5	0.434	0.215	0.254
6	0.493	0.195	0.387
7	0.370	0.249	0.278
8	0.578	0.525	0.787
9	0.387	0.200	0.272
10	0.363	0.183	0.230

Table S20. RMSD value for comparison of $(La_2Ce_2O_7)_4$ and $(La_2Zr_2O_7)_4$ with $(La_2Ti_2O_7)_4$ and between each other, $RMSD^{Ti-Ce}$, $RMSD^{Ti-Zr}$ and $RMSD^{Zr-Ce}$, respectively, measured in Å.

config.	$RMSD^{Ti-Ce}$	$RMSD^{Ti-Zr}$	$RMSD^{Zr-Ce}$
1	0.587	0.366	0.400
2	0.544	0.531	0.679
3	0.547	0.351	0.411
4	0.477	0.319	0.353
5	0.517	0.257	0.485
6	0.461	0.362	0.490
7	0.509	0.366	0.451
8	0.783	0.305	0.795
9	0.519	0.219	0.366
10	0.486	0.240	0.282
11	0.613	0.238	0.528
12	0.417	0.207	0.217
13	0.433	0.648	0.730
14	0.468	0.257	0.393
15	0.618	0.215	0.533
16	0.931	0.232	0.896
17	0.673	0.516	0.474
18	0.582	0.242	0.488
19	0.670	0.676	0.983
20	0.416	0.205	0.219

Table S21. RMSD value for comparison of $(La_2Ce_2O_7)_6$ and $(La_2Zr_2O_7)_6$ with $(La_2Ti_2O_7)_6$ and between each other, $RMSD^{Ti-Ce}$, $RMSD^{Ti-Zr}$ and $RMSD^{Zr-Ce}$, respectively, measured in Å.

config.	$RMSD^{Ti-Ce}$	$RMSD^{Ti-Zr}$	$RMSD^{Zr-Ce}$
1	0.521	0.375	0.363
2	0.508	0.297	0.275
3	0.743	0.453	0.778
4	0.523	0.245	0.334
5	0.510	0.375	0.523
6	0.677	0.428	0.713
7	0.652	0.436	0.614
8	0.672	0.456	0.562
9	0.608	0.260	0.495
10	0.763	0.380	0.649
11	0.899	0.384	0.808
12	0.566	0.277	0.360
13	0.704	0.244	0.637
14	0.710	0.352	0.501
15	0.719	0.496	0.501
16	0.630	0.369	0.434
17	0.848	0.434	0.681
18	0.610	0.295	0.428
19	0.743	0.496	0.623
20	0.760	0.530	0.771
21	0.576	0.326	0.382
22	0.621	0.328	0.452
23	0.744	0.438	0.604
24	0.931	0.471	0.973
25	0.474	0.239	0.288
26	0.511	0.270	0.298
27	0.789	0.275	0.698
28	0.779	0.615	0.600
29	0.729	0.357	0.608
30	0.624	0.318	0.416

Table S22. RMSD value for comparison of $(La_2Ce_2O_7)_8$ and $(La_2Zr_2O_7)_8$ with $(La_2Ti_2O_7)_8$ and between each other, $RMSD^{Ti-Ce}$, $RMSD^{Ti-Zr}$ and $RMSD^{Zr-Ce}$, respectively, measured in Å.

config.	$RMSD^{Ti-Ce}$	$RMSD^{Ti-Zr}$	$RMSD^{Zr-Ce}$
1	0.835	0.372	0.662
2	0.632	0.424	0.609
3	0.775	0.395	0.560
4	0.788	0.655	0.697
5	0.718	0.295	0.503
6	0.712	0.343	0.574
7	0.652	0.267	0.486
8	0.728	0.434	0.515
9	0.886	0.499	0.751
10	0.757	0.404	0.584
11	0.717	0.478	0.424
12	0.948	0.335	0.824
13	0.598	0.379	0.434
14	0.921	0.603	0.903
15	0.692	0.401	0.480
16	0.814	0.411	0.803
17	0.613	0.472	0.650
18	0.846	0.502	0.719
19	0.734	0.453	0.564
20	0.798	0.389	0.577
21	0.832	0.416	0.695
22	0.679	0.491	0.552
23	1.041	0.409	0.973
24	0.789	0.524	0.684
25	0.625	0.391	0.344
26	0.962	0.356	0.853
27	0.807	0.716	0.917
28	0.835	0.399	0.706
29	0.684	0.513	0.700
30	0.769	0.462	0.700
31	1.128	0.330	1.057
32	0.725	0.373	0.502
33	0.994	0.631	0.824
34	0.895	0.399	0.765
35	0.664	0.503	0.537
36	0.738	0.701	1.003
37	0.862	0.360	0.692
38	0.963	0.477	0.745
39	1.030	0.624	0.716
40	0.690	0.492	0.451

Table S23. RMSD value for comparison of $(La_2Ce_2O_7)_{10}$ and $(La_2Zr_2O_7)_{10}$ with $(La_2Ti_2O_7)_{10}$ and between each other, $RMSD^{Ti-Ce}$, $RMSD^{Ti-Zr}$ and $RMSD^{Zr-Ce}$, respectively, measured in Å.

config.	$RMSD^{Ti-Ce}$	$RMSD^{Ti-Zr}$	$RMSD^{Zr-Ce}$
1	0.759	0.333	0.597
2	0.781	0.529	0.638
3	0.712	0.347	0.527
4	0.740	0.510	0.591
5	0.683	0.675	0.632
6	0.760	0.396	0.652
7	0.680	0.501	0.435
8	0.734	0.429	0.546
9	0.796	0.546	0.616
10	0.774	0.387	0.680
11	0.751	0.445	0.511
12	0.720	0.470	0.371
13	0.888	0.390	0.750
14	0.707	0.418	0.524
15	0.712	0.379	0.467
16	0.682	0.695	0.819
17	0.817	0.324	0.673
18	0.885	0.388	0.834
19	0.720	0.379	0.475
20	0.646	0.407	0.455
21	0.779	1.008	1.239
22	0.929	0.550	0.869
23	0.815	0.402	0.720
24	0.767	0.450	0.770
25	0.780	0.431	0.697
26	0.831	0.553	0.693
27	0.815	0.574	0.546
28	0.593	0.382	0.437
29	0.860	0.389	0.703
30	0.690	0.412	0.418
31	0.834	0.404	0.666
32	0.757	0.564	0.661
33	0.957	0.509	0.828
34	0.913	0.527	0.778
35	0.952	0.448	0.858
36	0.771	0.603	0.759

Table S24. Continuation of Table S23.

config.	$RMSD^{Ti-Ce}$	$RMSD^{Ti-Zr}$	$RMSD^{Zr-Ce}$
37	0.857	0.440	0.769
38	0.810	0.617	0.744
39	0.946	0.321	0.850
40	0.877	0.694	0.940
41	0.906	0.491	0.916
42	0.980	0.616	0.917
43	0.690	0.355	0.479
44	0.698	0.374	0.525
45	0.865	0.554	0.864
46	0.862	0.589	0.740
47	1.067	0.590	0.995
48	0.772	0.529	0.634
49	0.860	0.314	0.761
50	0.829	0.527	0.591

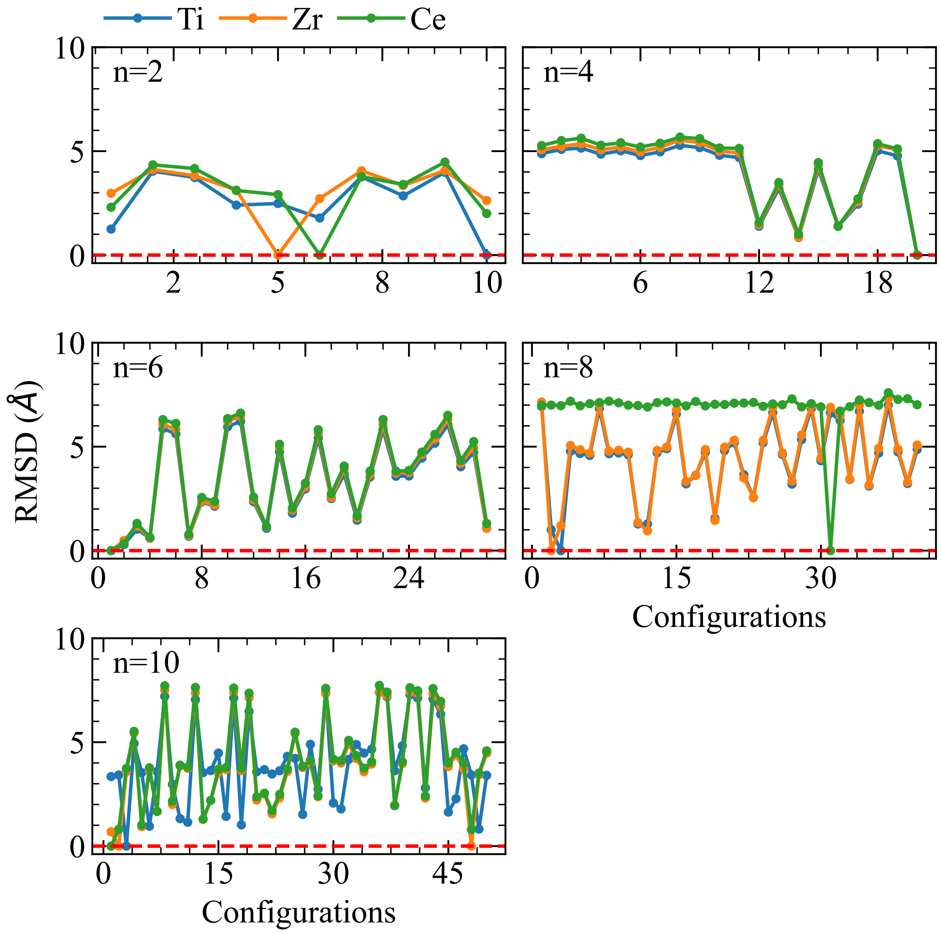

Figure S4. Root-Mean-Square Deviation. Comparison of structural differences between each $(La_2B_2O_7)_n$ structure (for corresponding n and B) and the lowest-energy configuration within the corresponding set.

S3.2 Radial Distribution Function

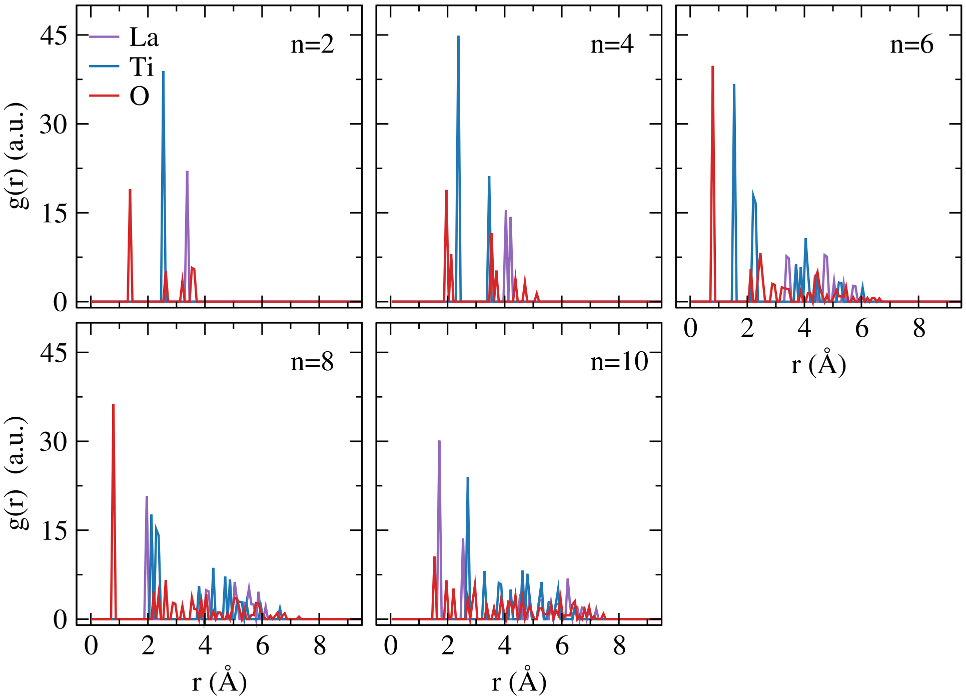

Figure S5. Partial Radial Distribution Function and Effective Coordination Number varying with the distance from the center of gravity of the $(La_2Ti_2O_7)_n$ nanoclusters.

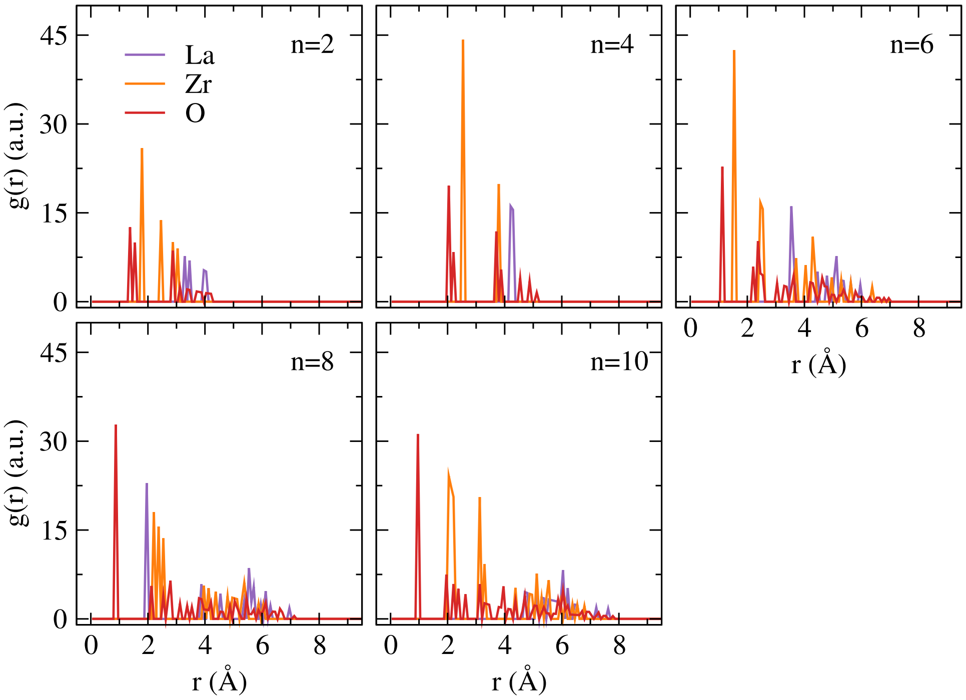

Figure S6. Partial Radial Distribution Function and Effective Coordination Number varying with the distance from the center of gravity of the $(La_2Zr_2O_7)_n$ nanoclusters.

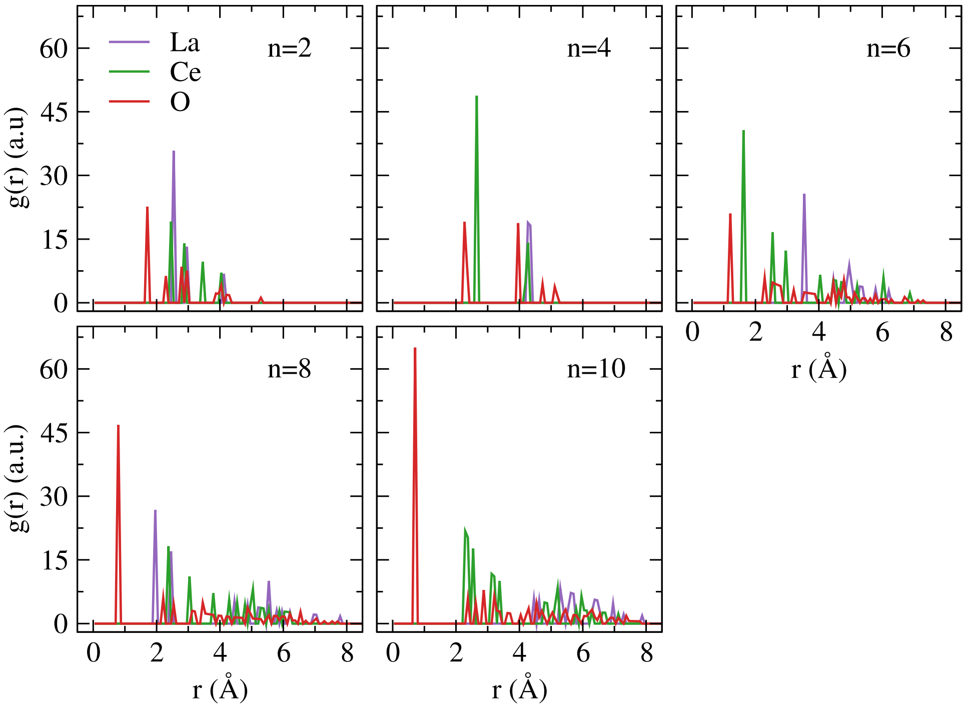

Figure S7. Partial Radial Distribution Function and Effective Coordination Number varying with the distance from the center of gravity of the $(La_2Ce_2O_7)_n$ nanoclusters.

S3.3 Average Bond Length Distance and Effective Coordination Number of La, B and O Species

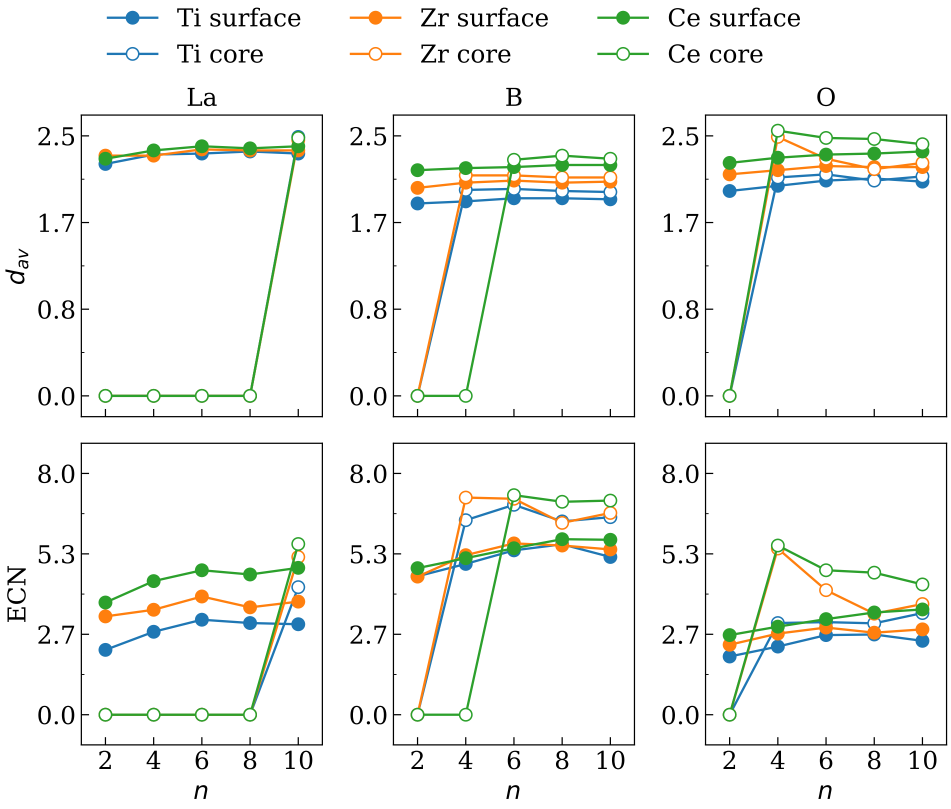

Figure S8. Average Bond Length Distance and Effective Coordination Number Core and Surface La, *B* and O Species. Null values indicate the absence of species in the core or surface region.

S3.4 Additional Features of Defect-Free Structures

Table S25. Properties of $(La_2Ti_2O_7)_2$ nanoclusters. Relative energy per atom, ΔE_{tot} (meV), HOMO-LUMO gap, E_g (eV), average radius of nanocluster, R_{av} (eV), average bond length distance of La, Ti and O, d_{av}^{La}, d_{av}^{Ti} and d_{av}^{O} (Å), respectively, average effective coordination number of La, Ti and O, $\mathrm{ECN}_{av}^{\mathrm{La}}$, $\mathrm{ECN}_{av}^{\mathrm{Ti}}$ and $\mathrm{ECN}_{av}^{\mathrm{O}}$ (NNN), respectively, and ratio of internal atoms to those exposed to the vacuum, n^{core}/n^{surf}.

ΔE_{tot}	E_g	R_{av}	d_{av}^{La}	d_{av}^{Ti}	d_{av}^{O}	$\mathrm{ECN}_{av}^{\mathrm{La}}$	$\mathrm{ECN}_{av}^{\mathrm{Ti}}$	$\mathrm{ECN}_{av}^{\mathrm{O}}$	$\frac{n^{core}}{n^{surf}}$
75	3.16	4.05	2.24	1.84	1.96	2.27	4.42	1.91	0.00
73	3.38	4.15	2.26	1.87	2.00	2.44	4.71	2.08	0.00
67	2.82	4.86	2.21	1.85	1.95	2.05	4.46	1.86	0.00
60	3.61	4.30	2.29	1.88	2.01	2.54	5.00	2.20	0.00
59	3.05	3.99	2.25	1.88	2.01	2.59	4.90	2.22	0.00
50	3.30	3.92	2.26	1.86	1.99	2.37	4.75	2.07	0.00
31	3.37	3.87	2.22	1.84	1.96	2.05	4.41	1.85	0.00
16	3.49	4.31	2.31	1.85	2.00	2.74	4.73	2.18	0.00
10	3.62	3.95	2.23	1.85	1.97	2.15	4.59	1.93	0.00
0	3.95	3.58	2.22	1.84	1.96	2.11	4.41	1.86	0.00

Table S26. Properties of $(La_2Ti_2O_7)_4$ nanoclusters. Relative energy per atom, ΔE_{tot} (meV), HOMO-LUMO gap, E_g (eV), average radius of nanocluster, R_{av} (eV), average bond length distance of La, Ti and O, d_{av}^{La}, d_{av}^{Ti} and d_{av}^{O} (Å), respectively, average effective coordination number of La, Ti and O, $\mathrm{ECN}_{av}^{\mathrm{La}}$, $\mathrm{ECN}_{av}^{\mathrm{Ti}}$ and $\mathrm{ECN}_{av}^{\mathrm{O}}$ (NNN), respectively, and ratio of internal atoms to those exposed to the vacuum, n^{core}/n^{surf}.

ΔE_{tot}	E_g	R_{av}	d_{av}^{La}	d_{av}^{Ti}	d_{av}^{O}	$\mathrm{ECN}_{av}^{\mathrm{La}}$	$\mathrm{ECN}_{av}^{\mathrm{Ti}}$	$\mathrm{ECN}_{av}^{\mathrm{O}}$	$\frac{n^{core}}{n^{surf}}$
76	2.97	5.10	2.32	1.88	2.03	2.68	5.15	2.31	0.10
72	3.08	6.04	2.31	1.88	2.02	2.59	5.24	2.29	0.10
71	2.93	6.39	2.26	1.88	2.01	2.56	4.93	2.18	0.05
67	3.06	5.17	2.32	1.89	2.05	2.86	5.33	2.45	0.02
65	2.74	5.16	2.31	1.88	2.03	2.73	5.12	2.30	0.05
62	2.99	5.51	2.34	1.88	2.04	2.90	5.09	2.34	0.05
61	3.30	5.65	2.31	1.88	2.03	2.82	5.12	2.33	0.13
59	2.93	5.26	2.32	1.91	2.06	3.06	5.65	2.63	0.07
56	3.15	5.43	2.30	1.91	2.04	2.61	5.48	2.39	0.05
55	2.83	4.67	2.38	1.93	2.12	3.59	6.20	3.10	0.02
52	3.08	5.59	2.29	1.87	2.01	2.45	5.18	2.22	0.19
51	2.87	5.29	2.32	1.88	2.03	2.75	5.18	2.32	0.10
51	2.95	5.27	2.31	1.90	2.04	2.81	5.32	2.40	0.07
46	3.05	5.29	2.32	1.87	2.03	2.96	5.12	2.36	0.05
45	2.91	5.41	2.32	1.87	2.03	2.83	5.07	2.31	0.05
45	3.27	5.33	2.33	1.92	2.07	2.88	5.91	2.64	0.05
42	3.31	5.31	2.38	1.92	2.11	3.55	6.00	3.04	0.05
24	3.67	5.77	2.32	1.87	2.03	2.81	5.04	2.29	0.16
4	3.26	5.11	2.36	1.96	2.13	3.46	6.61	3.14	0.02
0	3.33	5.10	2.36	1.96	2.13	3.42	6.71	3.17	0.05

Table S27. Properties of $(La_2Ti_2O_7)_6$ nanoclusters. Relative energy per atom, ΔE_{tot} (meV), HOMO-LUMO gap, E_g (eV), average radius of nanocluster, R_{av} (eV), average bond length distance of La, Ti and O, d_{av}^{La}, d_{av}^{Ti} and d_{av}^{O} (Å), respectively, average effective coordination number of La, Ti and O, $\mathrm{ECN}_{av}^{\mathrm{La}}$, $\mathrm{ECN}_{av}^{\mathrm{Ti}}$ and $\mathrm{ECN}_{av}^{\mathrm{O}}$ (NNN), respectively, and ratio of internal atoms to those exposed to the vacuum, n^{core}/n^{surf}.

ΔE_{tot}	E_g	R_{av}	d_{av}^{La}	d_{av}^{Ti}	d_{av}^{O}	$\mathrm{ECN}_{av}^{\mathrm{La}}$	$\mathrm{ECN}_{av}^{\mathrm{Ti}}$	$\mathrm{ECN}_{av}^{\mathrm{O}}$	$\frac{n^{core}}{n^{surf}}$
54	2.64	6.16	2.32	1.91	2.07	3.24	5.50	2.61	0.14
46	2.87	6.49	2.38	1.91	2.08	3.25	5.73	2.69	0.14
45	2.71	6.63	2.32	1.92	2.07	2.96	5.63	2.59	0.16
44	2.84	6.27	2.34	1.90	2.06	3.12	5.47	2.55	0.14
42	2.90	6.41	2.33	1.92	2.08	3.15	5.70	2.68	0.10
41	2.61	6.37	2.31	1.89	2.02	2.57	5.21	2.27	0.10
40	2.65	6.32	2.34	1.89	2.06	3.02	5.50	2.55	0.08
36	2.74	6.06	2.33	1.91	2.06	3.03	5.63	2.58	0.14
35	2.70	6.57	2.33	1.90	2.06	2.90	5.47	2.48	0.14
35	2.68	6.13	2.34	1.90	2.06	3.03	5.52	2.55	0.20
34	2.69	6.38	2.34	1.92	2.08	2.99	5.87	2.70	0.10
34	2.97	7.33	2.35	1.90	2.07	3.18	5.63	2.68	0.12
32	2.47	6.47	2.33	1.90	2.06	2.95	5.50	2.49	0.08
31	2.86	6.25	2.34	1.90	2.07	3.21	5.45	2.59	0.12
29	2.75	5.99	2.36	1.92	2.10	3.58	5.70	2.83	0.14
28	2.59	5.85	2.33	1.90	2.06	3.01	5.42	2.49	0.12
26	2.59	6.77	2.36	1.90	2.07	3.27	5.48	2.62	0.08
25	2.76	6.59	2.36	1.90	2.07	3.11	5.63	2.62	0.06
24	2.69	5.89	2.33	1.89	2.05	2.89	5.34	2.43	0.14
21	2.93	6.14	2.36	1.91	2.08	3.32	5.60	2.68	0.12
21	3.06	6.42	2.34	1.88	2.05	2.88	5.35	2.44	0.12
18	2.92	6.41	2.36	1.90	2.06	3.11	5.36	2.51	0.14
17	3.11	6.91	2.35	1.90	2.07	3.13	5.63	2.64	0.12
14	3.06	6.21	2.37	1.89	2.08	3.43	5.61	2.75	0.12
14	2.97	6.42	2.35	1.91	2.08	3.07	5.76	2.65	0.14
10	2.81	6.42	2.36	1.93	2.10	3.37	6.03	2.87	0.16
10	2.65	6.64	2.35	1.89	2.05	3.09	5.28	2.46	0.14
09	2.64	6.47	2.37	1.91	2.08	3.18	5.79	2.73	0.18
08	2.88	6.07	2.36	1.91	2.08	3.31	5.63	2.70	0.14
00	2.87	6.43	2.37	1.92	2.09	3.37	5.94	2.84	0.18

Table S28. Properties of $(La_2Ti_2O_7)_8$ nanoclusters. Relative energy per atom, ΔE_{tot} (meV), HOMO-LUMO gap, E_g (eV), average radius of nanocluster, R_{av} (eV), average bond length distance of La, Ti and O, d_{av}^{La}, d_{av}^{Ti} and d_{av}^{O} (Å), respectively, average effective coordination number of La, Ti and O, $\mathrm{ECN}_{av}^{\mathrm{La}}$, $\mathrm{ECN}_{av}^{\mathrm{Ti}}$ and $\mathrm{ECN}_{av}^{\mathrm{O}}$ (NNN), respectively, and ratio of internal atoms to those exposed to the vacuum, n^{core}/n^{surf}.

ΔE_{tot}	E_g	R_{av}	d_{av}^{La}	d_{av}^{Ti}	d_{av}^{O}	$\mathrm{ECN}_{av}^{\mathrm{La}}$	$\mathrm{ECN}_{av}^{\mathrm{Ti}}$	$\mathrm{ECN}_{av}^{\mathrm{O}}$	$\frac{n^{core}}{n^{surf}}$
56	2.64	7.16	2.35	1.93	2.10	3.42	5.96	2.89	0.17
53	2.63	8.10	2.33	1.91	2.07	3.04	5.56	2.56	0.16
52	3.02	7.38	2.31	1.90	2.04	2.75	5.45	2.41	0.07
52	2.60	6.93	2.36	1.94	2.12	3.45	6.19	3.00	0.16
49	2.68	7.05	2.35	1.92	2.09	3.35	5.79	2.80	0.21
49	2.80	6.96	2.35	1.91	2.08	3.17	5.67	2.69	0.14
41	2.55	7.22	2.35	1.90	2.08	3.26	5.52	2.66	0.21
40	2.56	6.64	2.31	1.91	2.05	2.88	5.46	2.49	0.10
40	2.84	6.61	2.34	1.91	2.07	3.25	5.53	2.63	0.19
39	2.57	6.66	2.36	1.92	2.08	3.05	5.85	2.68	0.17
39	2.59	7.28	2.34	1.90	2.06	2.89	5.67	2.54	0.13
38	2.89	7.28	2.34	1.90	2.05	2.77	5.57	2.47	0.16
38	2.50	6.53	2.36	1.91	2.09	3.28	5.71	2.70	0.14
37	2.81	7.01	2.37	1.92	2.10	3.45	5.71	2.77	0.22
35	2.90	7.31	2.35	1.90	2.06	2.83	5.64	2.52	0.11
34	2.70	6.86	2.35	1.93	2.08	3.03	5.93	2.71	0.17
34	2.56	7.19	2.35	1.90	2.07	3.11	5.40	2.53	0.19
33	3.03	7.51	2.35	1.90	2.06	3.13	5.43	2.53	0.17
30	2.60	7.30	2.36	1.87	2.05	3.13	5.12	2.42	0.09
29	3.01	7.49	2.36	1.92	2.08	3.03	5.91	2.68	0.19
28	2.40	6.61	2.39	1.92	2.11	3.60	5.83	2.88	0.21
28	2.37	6.87	2.35	1.90	2.06	3.03	5.42	2.50	0.22
28	2.47	7.12	2.35	1.94	2.09	3.11	6.18	2.83	0.19
28	2.65	7.10	2.34	1.89	2.06	3.14	5.36	2.51	0.07
27	2.65	6.90	2.37	1.90	2.08	3.42	5.58	2.74	0.11
26	2.87	7.20	2.36	1.90	2.07	3.20	5.44	2.60	0.21
26	2.97	7.52	2.37	1.89	2.08	3.36	5.46	2.67	0.16
25	2.81	7.30	2.36	1.91	2.07	2.95	5.73	2.59	0.21
24	2.81	6.54	2.38	1.93	2.12	3.53	6.13	2.99	0.16
23	2.94	7.10	2.36	1.90	2.08	3.30	5.46	2.61	0.14
23	2.79	7.17	2.36	1.91	2.08	3.04	5.82	2.66	0.19
21	2.13	7.37	2.35	1.89	2.06	3.10	5.30	2.48	0.17
20	2.98	6.83	2.36	1.89	2.08	3.47	5.47	2.69	0.11
18	2.78	6.97	2.36	1.92	2.10	3.46	5.81	2.83	0.16
17	3.02	7.21	2.34	1.91	2.07	3.02	5.74	2.61	0.07
15	2.65	6.84	2.39	1.92	2.11	3.53	6.07	2.96	0.24
14	2.65	7.25	2.36	1.90	2.06	2.97	5.62	2.57	0.19
12	2.83	6.64	2.37	1.90	2.09	3.45	5.61	2.76	0.16
10	2.95	6.63	2.40	1.91	2.11	3.54	5.88	2.86	0.17
0	2.89	7.12	2.39	1.90	2.09	3.46	5.70	2.76	0.16

Table S29. Properties of $(La_2Ti_2O_7)_{10}$ nanoclusters. Relative energy per atom, ΔE_{tot} (meV), HOMO-LUMO gap, E_g (eV), average radius of nanocluster, R_{av} (eV), average bond length distance of La, Ti and O, d_{av}^{La}, d_{av}^{Ti} and d_{av}^{O} (Å), respectively, average effective coordination number of La, Ti and O, $\text{ECN}_{av}^{\text{La}}$, $\text{ECN}_{av}^{\text{Ti}}$ and $\text{ECN}_{av}^{\text{O}}$ (NNN), respectively, and ratio of internal atoms to those exposed to the vacuum, n^{core}/n^{surf}.

ΔE_{tot}	E_g	R_{av}	d_{av}^{La}	d_{av}^{Ti}	d_{av}^{O}	$\text{ECN}_{av}^{\text{La}}$	$\text{ECN}_{av}^{\text{Ti}}$	$\text{ECN}_{av}^{\text{O}}$	$\frac{n^{core}}{n^{surf}}$
58	2.28	7.81	2.35	1.92	2.08	3.05	5.87	2.71	0.25
58	2.54	7.54	2.34	1.90	2.06	3.17	5.44	2.56	0.15
57	2.60	7.52	2.35	1.92	2.09	3.16	5.87	2.73	0.18
55	2.71	7.18	2.37	1.91	2.09	3.55	5.48	2.70	0.28
53	2.50	7.58	2.36	1.92	2.09	3.30	5.94	2.84	0.28
52	2.77	7.10	2.36	1.93	2.10	3.61	5.82	2.89	0.22
51	2.67	7.78	2.36	1.92	2.10	3.39	5.85	2.81	0.24
50	2.54	7.92	2.33	1.91	2.06	3.01	5.58	2.58	0.22
50	2.55	7.75	2.34	1.89	2.06	3.13	5.33	2.50	0.15
50	2.55	7.37	2.37	1.93	2.10	3.29	6.06	2.87	0.25
49	2.75	8.57	2.37	1.92	2.10	3.41	6.05	2.93	0.16
48	2.65	7.62	2.35	1.92	2.09	3.20	5.83	2.74	0.24
48	2.00	7.61	2.35	1.90	2.07	3.03	5.53	2.57	0.18
44	2.86	7.79	2.35	1.91	2.08	3.45	5.45	2.66	0.25
44	2.73	7.48	2.38	1.90	2.08	3.23	5.63	2.64	0.24
43	2.44	7.43	2.34	1.93	2.09	3.21	5.94	2.77	0.28
42	2.72	7.23	2.34	1.91	2.08	3.23	5.64	2.65	0.20
42	2.40	8.28	2.35	1.91	2.08	3.35	5.69	2.72	0.25
41	1.95	8.44	2.35	1.90	2.08	3.47	5.49	2.72	0.18
40	2.59	7.31	2.35	1.90	2.06	2.96	5.42	2.47	0.17
39	2.56	7.98	2.35	1.92	2.09	3.38	5.78	2.79	0.18
37	2.35	7.79	2.33	1.89	2.04	2.80	5.20	2.33	0.16
36	2.30	7.79	2.35	1.92	2.08	3.09	5.83	2.69	0.21
35	2.70	7.34	2.35	1.92	2.09	3.19	5.88	2.74	0.22
34	2.37	7.52	2.34	1.91	2.07	3.06	5.63	2.61	0.21
34	2.50	7.76	2.36	1.89	2.06	3.10	5.49	2.55	0.15
34	2.77	7.86	2.36	1.91	2.08	3.32	5.60	2.69	0.18
32	2.59	7.77	2.36	1.92	2.09	3.25	5.80	2.71	0.29
30	2.78	7.34	2.38	1.93	2.10	3.37	5.99	2.86	0.29
30	2.71	7.39	2.37	1.90	2.07	3.08	5.59	2.58	0.24
30	2.66	7.21	2.39	1.91	2.11	3.60	5.83	2.92	0.21
29	2.90	8.21	2.36	1.88	2.06	3.06	5.34	2.48	0.16
29	2.50	7.32	2.36	1.89	2.06	3.32	5.31	2.57	0.16
25	3.01	7.96	2.38	1.90	2.08	3.39	5.56	2.67	0.24

Table S30. Continuation of Table S29

ΔE_{tot}	E_g	R_{av}	d_{av}^{La}	d_{av}^{Ti}	d_{av}^{O}	$\mathrm{ECN}_{av}^{\mathrm{La}}$	$\mathrm{ECN}_{av}^{\mathrm{Ti}}$	$\mathrm{ECN}_{av}^{\mathrm{O}}$	$\frac{n^{core}}{n^{surf}}$
23	2.72	7.73	2.38	1.89	2.07	3.08	5.42	2.51	0.21
23	2.87	7.30	2.38	1.92	2.10	3.31	5.87	2.76	0.25
22	2.81	7.80	2.38	1.91	2.08	3.28	5.78	2.74	0.18
22	3.11	8.06	2.37	1.91	2.09	3.35	5.78	2.76	0.26
19	2.74	7.35	2.38	1.90	2.09	3.33	5.75	2.74	0.18
19	2.66	7.86	2.38	1.92	2.12	3.77	5.94	3.01	0.21
19	2.96	7.29	2.36	1.91	2.09	3.34	5.74	2.73	0.21
18	2.67	7.69	2.38	1.91	2.09	3.42	5.80	2.81	0.22
17	2.75	7.49	2.38	1.91	2.09	3.25	5.86	2.75	0.29
16	2.85	7.71	2.38	1.91	2.10	3.52	5.86	2.88	0.20
15	2.87	7.32	2.38	1.92	2.09	3.25	5.83	2.70	0.28
15	2.86	7.32	2.38	1.92	2.09	3.25	5.83	2.70	0.28
14	2.70	7.29	2.38	1.93	2.11	3.38	6.13	2.88	0.28
09	2.84	7.88	2.38	1.89	2.08	3.24	5.64	2.66	0.20
07	3.10	7.77	2.37	1.90	2.07	3.23	5.63	2.66	0.17
00	3.00	7.22	2.39	1.92	2.11	3.56	5.96	2.92	0.22

Table S31. Properties of $(La_2Zr_2O_7)_2$ nanoclusters. Relative energy per atom, ΔE_{tot} (meV), HOMO-LUMO gap, E_g (eV), average radius of nanocluster, R_{av} (eV), average bond length distance of La, Zr and O, d_{av}^{La}, d_{av}^{Zr} and d_{av}^{O} (Å), respectively, average effective coordination number of La, Zr and O, $\mathrm{ECN}_{av}^{\mathrm{La}}$, $\mathrm{ECN}_{av}^{\mathrm{Zr}}$ and $\mathrm{ECN}_{av}^{\mathrm{O}}$ (NNN), respectively, and ratio of internal atoms to those exposed to the vacuum, n^{core}/n^{surf}.

ΔE_{tot}	E_g	R_{av}	d_{av}^{La}	d_{av}^{Zr}	d_{av}^{O}	$\mathrm{ECN}_{av}^{\mathrm{La}}$	$\mathrm{ECN}_{av}^{\mathrm{Zr}}$	$\mathrm{ECN}_{av}^{\mathrm{O}}$	$\frac{n^{core}}{n^{surf}}$
93	2.47	4.33	2.25	2.00	2.09	2.86	4.37	2.08	0.00
82	2.36	5.14	2.22	2.00	2.08	2.55	4.36	1.98	0.00
62	3.02	4.64	2.29	2.04	2.14	3.17	5.06	2.44	0.00
61	3.27	4.30	2.27	2.02	2.13	2.98	4.75	2.26	0.00
41	3.33	4.10	2.28	2.02	2.13	2.90	4.80	2.28	0.00
39	2.97	4.62	2.31	2.00	2.13	3.26	4.58	2.32	0.00
15	3.23	4.13	2.26	2.07	2.16	3.14	5.37	2.59	0.00
10	3.17	4.08	2.23	2.01	2.10	2.68	4.46	2.05	0.00
6	3.64	3.75	2.22	1.99	2.08	2.54	4.31	1.96	0.00
0	3.35	4.09	2.24	2.01	2.10	2.67	4.55	2.08	0.00

Table S32. Properties of $(La_2Zr_2O_7)_4$ nanoclusters. Relative energy per atom, ΔE_{tot} (meV), HOMO-LUMO gap, E_g (eV), average radius of nanocluster, R_{av} (eV), average bond length distance of La, Zr and O, d_{av}^{La}, d_{av}^{Zr} and d_{av}^{O} (Å), respectively, average effective coordination number of La, Zr and O, $\mathrm{ECN}_{av}^{\mathrm{La}}$, $\mathrm{ECN}_{av}^{\mathrm{Zr}}$ and $\mathrm{ECN}_{av}^{\mathrm{O}}$ (NNN), respectively, and ratio of internal atoms to those exposed to the vacuum, n^{core}/n^{surf}.

ΔE_{tot}	E_g	R_{av}	d_{av}^{La}	d_{av}^{Zr}	d_{av}^{O}	$\mathrm{ECN}_{av}^{\mathrm{La}}$	$\mathrm{ECN}_{av}^{\mathrm{Zr}}$	$\mathrm{ECN}_{av}^{\mathrm{O}}$	$\frac{n^{core}}{n^{surf}}$
144	2.91	6.44	2.31	2.06	2.18	3.25	5.59	2.72	0.02
138	3.01	5.40	2.33	2.04	2.17	3.33	5.31	2.63	0.05
137	3.26	5.84	2.32	2.03	2.16	3.34	5.14	2.52	0.00
133	2.76	6.69	2.27	2.03	2.13	3.07	4.95	2.35	0.02
127	3.23	5.75	2.30	2.03	2.14	2.98	5.13	2.38	0.02
125	3.06	5.41	2.32	2.06	2.18	3.43	5.60	2.82	0.05
125	2.91	5.68	2.32	2.02	2.15	3.29	4.96	2.42	0.05
123	3.29	5.62	2.32	2.02	2.15	3.37	4.97	2.45	0.05
112	3.15	5.73	2.31	2.06	2.17	3.19	5.50	2.61	0.05
107	2.86	5.44	2.31	2.05	2.16	3.20	5.27	2.49	0.05
101	3.00	5.41	2.33	2.06	2.18	3.54	5.62	2.79	0.02
101	3.40	5.36	2.31	2.06	2.18	3.48	5.53	2.79	0.05
99	2.99	5.69	2.32	2.08	2.21	3.64	5.95	3.04	0.05
95	3.35	5.54	2.34	2.05	2.18	3.60	5.48	2.73	0.05
89	3.68	5.53	2.38	2.06	2.23	4.00	5.92	3.21	0.07
88	3.32	4.93	2.36	2.09	2.24	4.09	6.26	3.42	0.10
87	3.54	6.03	2.32	2.05	2.18	3.61	5.35	2.72	0.05
84	3.39	5.46	2.35	2.07	2.20	3.57	5.96	2.90	0.07
6	3.47	5.15	2.38	2.10	2.25	4.10	6.60	3.40	0.16
0	3.61	5.15	2.38	2.10	2.25	4.07	6.67	3.42	0.16

Table S33. Properties of $(La_2Zr_2O_7)_6$ nanoclusters. Relative energy per atom, ΔE_{tot} (meV), HOMO-LUMO gap, E_g (eV), average radius of nanocluster, R_{av} (eV), average bond length distance of La, Zr and O, d_{av}^{La}, d_{av}^{Zr} and d_{av}^{O} (Å), respectively, average effective coordination number of La, Zr and O, $\mathrm{ECN}_{av}^{\mathrm{La}}$, $\mathrm{ECN}_{av}^{\mathrm{Zr}}$ and $\mathrm{ECN}_{av}^{\mathrm{O}}$ (NNN), respectively, and ratio of internal atoms to those exposed to the vacuum, n^{core}/n^{surf}.

ΔE_{tot}	E_g	R_{av}	d_{av}^{La}	d_{av}^{Zr}	d_{av}^{O}	$\mathrm{ECN}_{av}^{\mathrm{La}}$	$\mathrm{ECN}_{av}^{\mathrm{Zr}}$	$\mathrm{ECN}_{av}^{\mathrm{O}}$	$\frac{n^{core}}{n^{surf}}$
69	3.06	6.71	2.38	2.06	2.21	3.78	5.68	2.88	0.14
65	2.71	6.73	2.32	2.04	2.16	3.29	5.24	2.53	0.08
58	3.18	6.54	2.35	2.05	2.19	3.62	5.43	2.72	0.16
57	3.07	6.72	2.34	2.04	2.17	3.48	5.33	2.66	0.12
57	3.01	6.65	2.34	2.05	2.18	3.58	5.41	2.70	0.08
51	3.11	6.27	2.33	2.06	2.18	3.41	5.53	2.72	0.10
51	3.12	6.65	2.35	2.06	2.20	3.67	5.67	2.88	0.05
49	2.74	6.28	2.33	2.07	2.19	3.63	5.50	2.77	0.08
47	2.88	6.85	2.37	2.06	2.21	3.81	5.71	2.94	0.06
47	3.29	7.52	2.34	2.06	2.19	3.64	5.61	2.87	0.08
41	2.69	6.34	2.35	2.06	2.20	3.64	5.73	2.90	0.12
40	2.99	6.61	2.35	2.08	2.22	3.52	6.05	3.05	0.10
40	3.29	6.90	2.35	2.07	2.22	3.70	5.99	3.08	0.16
40	3.10	6.91	2.34	2.07	2.20	3.58	5.80	2.89	0.10
39	2.54	6.11	2.34	2.07	2.20	3.52	5.88	2.91	0.05
38	2.79	6.62	2.34	2.06	2.19	3.53	5.65	2.78	0.10
37	3.32	6.89	2.36	2.06	2.20	3.71	5.73	2.91	0.08
32	2.50	6.91	2.36	2.05	2.18	3.67	5.35	2.68	0.12
31	3.45	6.50	2.38	2.05	2.22	4.09	5.73	3.15	0.14
30	3.04	6.41	2.36	2.06	2.20	3.85	5.60	2.88	0.08
29	3.26	6.75	2.36	2.07	2.22	3.84	5.87	3.05	0.12
24	2.94	6.13	2.36	2.09	2.23	3.93	6.03	3.18	0.12
24	2.83	6.26	2.35	2.06	2.20	3.76	5.69	2.93	0.14
22	3.29	6.53	2.35	2.09	2.23	3.86	6.14	3.20	0.14
21	3.46	6.71	2.36	2.06	2.20	3.61	5.74	2.85	0.16
18	3.34	6.54	2.38	2.07	2.22	3.92	5.94	3.10	0.14
18	3.41	7.20	2.36	2.06	2.21	3.68	5.75	2.90	0.10
12	2.64	6.76	2.36	2.09	2.23	3.76	6.30	3.23	0.16
4	2.93	6.73	2.37	2.08	2.23	3.99	6.07	3.17	0.18
0	3.40	6.71	2.37	2.08	2.22	3.92	5.92	3.06	0.14

Table S34. Properties of $(La_2Zr_2O_7)_8$ nanoclusters. Relative energy per atom, ΔE_{tot} (meV), HOMO-LUMO gap, E_g (eV), average radius of nanocluster, R_{av} (eV), average bond length distance of La, Zr and O, d_{av}^{La}, d_{av}^{Zr} and d_{av}^{O} (Å), respectively, average effective coordination number of La, Zr and O, $\mathrm{ECN}_{av}^{\mathrm{La}}$, $\mathrm{ECN}_{av}^{\mathrm{Zr}}$ and $\mathrm{ECN}_{av}^{\mathrm{O}}$ (NNN), respectively, and ratio of internal atoms to those exposed to the vacuum, n^{core}/n^{surf}.

ΔE_{tot}	E_g	R_{av}	d_{av}^{La}	d_{av}^{Zr}	d_{av}^{O}	$\mathrm{ECN}_{av}^{\mathrm{La}}$	$\mathrm{ECN}_{av}^{\mathrm{Zr}}$	$\mathrm{ECN}_{av}^{\mathrm{O}}$	$\frac{n^{core}}{n^{surf}}$
49	2.49	8.49	2.34	2.07	2.20	3.63	5.76	2.88	0.13
49	1.91	7.46	2.36	2.05	2.19	3.77	5.55	2.87	0.10
49	3.16	7.78	2.35	2.07	2.21	3.50	5.96	2.92	0.13
46	3.19	7.66	2.33	2.07	2.19	3.59	5.70	2.82	0.09
45	2.96	7.12	2.36	2.06	2.20	3.68	5.59	2.83	0.17
42	3.06	7.15	2.36	2.06	2.20	3.79	5.64	2.91	0.13
40	2.96	7.62	2.36	2.06	2.20	3.56	5.75	2.81	0.14
40	2.68	6.89	2.36	2.06	2.20	3.68	5.70	2.86	0.13
39	2.37	7.67	2.36	2.05	2.20	3.72	5.50	2.84	0.14
39	2.76	7.41	2.34	2.06	2.19	3.69	5.56	2.80	0.09
38	3.00	7.67	2.37	2.06	2.22	3.90	5.75	3.02	0.14
38	2.71	7.07	2.34	2.08	2.21	3.90	5.97	3.13	0.13
37	3.04	7.49	2.37	2.08	2.23	3.95	6.09	3.20	0.13
33	3.39	7.21	2.37	2.08	2.23	3.97	6.04	3.17	0.13
32	2.91	7.69	2.34	2.07	2.20	3.73	5.75	2.91	0.19
31	3.17	7.71	2.35	2.07	2.21	3.66	5.84	2.93	0.17
27	3.24	7.65	2.35	2.07	2.20	3.62	5.73	2.82	0.16
25	3.29	7.44	2.35	2.06	2.19	3.55	5.67	2.76	0.06
25	2.45	6.75	2.36	2.08	2.22	3.89	5.95	3.09	0.19
24	3.16	7.17	2.38	2.05	2.21	4.04	5.62	3.00	0.13
24	3.02	7.46	2.37	2.08	2.23	4.05	6.01	3.15	0.17
22	3.03	6.97	2.39	2.08	2.25	4.16	6.06	3.25	0.19
22	2.76	7.60	2.36	2.06	2.20	3.62	5.79	2.87	0.16
20	3.05	7.15	2.35	2.07	2.21	3.75	5.89	2.99	0.11
19	2.87	7.47	2.35	2.07	2.21	3.87	5.78	2.97	0.16
18	3.29	7.12	2.39	2.06	2.22	4.00	5.74	2.98	0.14
18	3.07	7.72	2.35	2.07	2.20	3.53	5.87	2.86	0.21
17	3.06	6.81	2.37	2.10	2.26	3.95	6.63	3.54	0.17
16	3.20	7.24	2.35	2.08	2.21	3.60	6.05	2.95	0.16
16	2.72	7.43	2.37	2.08	2.21	3.61	5.99	2.91	0.17
15	3.01	6.91	2.38	2.07	2.23	4.02	5.82	3.10	0.16
15	2.84	7.22	2.36	2.08	2.22	4.06	5.96	3.17	0.11
14	3.16	7.72	2.35	2.08	2.22	3.86	5.89	3.05	0.17
13	2.55	7.18	2.37	2.07	2.22	3.92	5.89	3.05	0.17
11	3.33	7.45	2.36	2.08	2.22	3.67	6.14	3.02	0.19
11	2.98	6.92	2.39	2.08	2.24	4.17	6.12	3.28	0.19
6	2.94	7.04	2.35	2.11	2.25	3.92	6.53	3.41	0.19
4	3.16	8.08	2.38	2.08	2.22	3.80	6.08	3.06	0.16
3	2.87	6.82	2.38	2.10	2.26	4.24	6.44	3.53	0.16
0	2.88	7.00	2.38	2.07	2.23	4.01	6.10	3.19	0.14

Table S35. Properties of $(La_2Zr_2O_7)_{10}$ nanoclusters. Relative energy per atom, ΔE_{tot} (meV), HOMO-LUMO gap, E_g (eV), average radius of nanocluster, R_{av} (eV), average bond length distance of La, Zr and O, d_{av}^{La}, d_{av}^{Zr} and d_{av}^{O} (Å), respectively, average effective coordination number of La, Zr and O, $\mathrm{ECN}_{av}^{\mathrm{La}}$, $\mathrm{ECN}_{av}^{\mathrm{Zr}}$ and $\mathrm{ECN}_{av}^{\mathrm{O}}$ (NNN), respectively, and ratio of internal atoms to those exposed to the vacuum, n^{core}/n^{surf}.

ΔE_{tot}	E_g	R_{av}	d_{av}^{La}	d_{av}^{Ti}	d_{av}^{O}	$\mathrm{ECN}_{av}^{\mathrm{La}}$	$\mathrm{ECN}_{av}^{\mathrm{Ti}}$	$\mathrm{ECN}_{av}^{\mathrm{O}}$	$\frac{n^{core}}{n^{surf}}$
64	2.17	8.06	2.34	2.06	2.19	3.63	5.50	2.75	0.11
52	3.09	8.16	2.34	2.05	2.18	3.46	5.41	2.64	0.11
49	3.17	7.65	2.39	2.07	2.23	4.01	6.00	3.13	0.24
47	1.29	9.10	2.36	2.06	2.21	3.99	5.63	2.95	0.17
46	2.81	8.64	2.36	2.05	2.20	3.78	5.60	2.88	0.12
45	2.36	7.37	2.36	2.09	2.23	3.99	6.02	3.16	0.24
45	3.10	7.57	2.37	2.07	2.21	3.75	5.82	2.97	0.18
44	2.56	7.97	2.35	2.08	2.22	3.72	6.02	3.04	0.24
43	2.85	7.74	2.37	2.08	2.23	3.87	6.15	3.18	0.20
41	2.95	7.62	2.35	2.06	2.20	3.85	5.64	2.92	0.15
41	2.96	7.93	2.36	2.08	2.22	3.94	6.02	3.11	0.15
40	2.86	8.35	2.36	2.07	2.21	3.82	5.78	2.97	0.18
40	2.83	8.91	2.37	2.08	2.23	3.92	6.12	3.17	0.18
39	3.11	8.29	2.38	2.06	2.22	3.95	5.74	2.97	0.21
39	2.47	8.10	2.37	2.07	2.22	3.82	5.91	3.03	0.16
38	3.14	7.65	2.37	2.08	2.22	3.73	5.97	3.02	0.22
36	2.29	7.70	2.36	2.08	2.22	3.71	6.11	3.07	0.22
34	2.99	7.61	2.40	2.06	2.22	4.02	5.83	3.04	0.18
34	3.04	7.60	2.36	2.09	2.23	3.92	6.18	3.21	0.16
34	2.76	7.64	2.36	2.08	2.22	3.98	5.93	3.09	0.13
33	2.84	7.44	2.37	2.09	2.23	4.08	6.10	3.22	0.20
33	3.00	7.61	2.40	2.07	2.24	4.23	5.94	3.24	0.18
33	2.82	8.10	2.37	2.09	2.23	3.96	6.12	3.23	0.15
32	2.62	8.85	2.35	2.08	2.22	3.81	6.04	3.06	0.20
32	2.66	8.12	2.37	2.05	2.20	3.83	5.62	2.87	0.17
32	3.11	8.06	2.36	2.09	2.22	3.68	6.19	3.11	0.24
31	2.43	8.62	2.36	2.06	2.20	3.82	5.69	2.93	0.16
31	2.38	8.26	2.37	2.10	2.25	4.09	6.37	3.42	0.22
28	3.06	8.22	2.37	2.08	2.22	4.05	5.78	3.03	0.20
26	2.83	8.09	2.38	2.07	2.22	3.93	5.92	3.06	0.17
26	3.11	7.89	2.36	2.06	2.20	3.75	5.72	2.89	0.12
26	3.35	8.35	2.38	2.07	2.22	3.94	5.93	3.11	0.17
25	2.92	7.67	2.36	2.09	2.23	3.80	6.27	3.17	0.21
19	2.96	7.75	2.36	2.11	2.25	3.90	6.68	3.50	0.18
17	3.08	8.01	2.40	2.07	2.24	4.11	6.03	3.22	0.15

Table S36. Continuation of Table S35.

ΔE_{tot}	E_g	R_{av}	d_{av}^{La}	d_{av}^{Zr}	d_{av}^{O}	$\mathrm{ECN}_{av}^{\mathrm{La}}$	$\mathrm{ECN}_{av}^{\mathrm{Zr}}$	$\mathrm{ECN}_{av}^{\mathrm{O}}$	$\frac{n^{core}}{n^{surf}}$
17	2.85	7.69	2.36	2.07	2.21	3.80	5.76	2.91	0.15
16	2.73	7.79	2.37	2.09	2.24	3.93	6.38	3.30	0.22
16	2.07	8.07	2.39	2.08	2.24	4.34	6.05	3.32	0.16
16	3.19	7.84	2.39	2.07	2.24	4.17	6.05	3.23	0.25
15	2.71	7.65	2.37	2.09	2.23	3.83	6.26	3.22	0.17
14	3.00	8.18	2.38	2.08	2.24	4.04	6.14	3.23	0.20
14	2.52	7.89	2.37	2.10	2.24	3.84	6.48	3.33	0.22
11	3.42	7.69	2.39	2.09	2.25	4.13	6.24	3.29	0.24
8	2.99	8.11	2.37	2.09	2.24	3.90	6.36	3.25	0.18
7	2.69	7.61	2.39	2.08	2.23	3.98	6.16	3.13	0.20
5	3.07	7.66	2.38	2.09	2.25	4.10	6.33	3.30	0.22
2	2.92	7.56	2.40	2.07	2.23	4.11	6.01	3.17	0.17
1	2.84	7.76	2.38	2.09	2.24	3.99	6.24	3.24	0.26
0	3.05	7.53	2.40	2.09	2.25	4.22	6.32	3.36	0.28
0	3.05	7.53	2.40	2.09	2.25	4.22	6.32	3.36	0.26

Table S37. Properties of $(La_2Ce_2O_7)_2$ nanoclusters. Relative energy per atom, ΔE_{tot}, HOMO-LUMO gap, E_g, average radius of nanocluster, R_{av}, average bond length distance of La, Ce and O, d_{av}^{La}, d_{av}^{Ce} and d_{av}^{O}, respectively, average effective coordination number of La, Ce and O, $\mathrm{ECN}_{av}^{\mathrm{La}}$, $\mathrm{ECN}_{av}^{\mathrm{Ce}}$ and $\mathrm{ECN}_{av}^{\mathrm{O}}$, respectively, and ratio of internal atoms to those exposed to the vacuum, n^{core}/n^{surf}.

ΔE_{tot}	E_g	R_{av}	d_{av}^{La}	d_{av}^{Ce}	d_{av}^{O}	$\mathrm{ECN}_{av}^{\mathrm{La}}$	$\mathrm{ECN}_{av}^{\mathrm{Ce}}$	$\mathrm{ECN}_{av}^{\mathrm{O}}$	$\frac{n^{core}}{n^{surf}}$
81	1.41	5.48	2.24	2.13	2.18	3.11	4.13	2.09	0.00
80	1.75	4.59	2.26	2.13	2.18	3.20	4.19	2.14	0.00
48	2.35	3.93	2.25	2.14	2.19	2.96	4.31	2.10	0.00
47	2.19	4.34	2.30	2.16	2.23	3.53	4.71	2.48	0.00
44	2.35	4.83	2.31	2.18	2.25	3.87	4.83	2.68	0.00
35	1.87	4.23	2.30	2.18	2.24	3.64	4.90	2.64	0.00
34	1.98	4.26	2.30	2.16	2.24	3.63	4.73	2.57	0.00
23	2.20	4.30	2.26	2.16	2.21	3.44	4.56	2.38	0.00
13	2.33	4.25	2.28	2.17	2.24	3.72	4.86	2.64	0.00
0	2.22	4.91	2.33	2.16	2.26	4.24	4.81	2.88	0.00

Table S38. Properties of $(La_2Ce_2O_7)_4$ nanoclusters. Relative energy per atom, ΔE_{tot} (meV), HOMO-LUMO gap, E_g (eV), average radius of nanocluster, R_{av} (eV), average bond length distance of La, Ce and O, d_{av}^{La}, d_{av}^{Ce} and d_{av}^{O} (Å), respectively, average effective coordination number of La, Ce and O, $\mathrm{ECN}_{av}^{\mathrm{La}}$, $\mathrm{ECN}_{av}^{\mathrm{Ce}}$ and $\mathrm{ECN}_{av}^{\mathrm{O}}$ (NNN), respectively, and ratio of internal atoms to those exposed to the vacuum, n^{core}/n^{surf}.

ΔE_{tot}	E_g	R_{av}	d_{av}^{La}	d_{av}^{Ce}	d_{av}^{O}	$\mathrm{ECN}_{av}^{\mathrm{La}}$	$\mathrm{ECN}_{av}^{\mathrm{Ce}}$	$\mathrm{ECN}_{av}^{\mathrm{O}}$	$\frac{n^{core}}{n^{surf}}$
141	1.83	5.82	2.32	2.19	2.28	4.03	5.27	2.95	0.00
137	1.81	5.81	2.35	2.19	2.30	4.10	5.43	3.05	0.15
135	2.00	6.77	2.33	2.19	2.28	3.99	5.32	2.92	0.05
134	1.64	6.04	2.34	2.17	2.27	4.09	4.98	2.80	0.00
134	1.84	6.74	2.30	2.20	2.27	3.78	5.40	2.86	0.05
126	1.63	6.02	2.36	2.17	2.28	4.28	5.07	2.89	0.05
126	1.84	5.65	2.36	2.18	2.28	4.16	5.15	2.88	0.07
124	1.85	5.36	2.40	2.20	2.34	4.64	5.72	3.48	0.10
124	1.95	5.91	2.34	2.20	2.29	4.13	5.39	3.02	0.10
122	2.00	5.70	2.36	2.18	2.29	4.22	5.13	2.85	0.10
121	2.14	5.99	2.35	2.17	2.28	4.29	5.01	2.87	0.10
118	1.73	5.65	2.34	2.21	2.29	4.08	5.41	2.95	0.10
117	1.77	5.94	2.34	2.20	2.29	3.91	5.41	2.89	0.05
105	2.12	6.34	2.36	2.19	2.30	4.43	5.19	3.02	0.05
098	2.11	5.83	2.40	2.21	2.35	4.93	5.80	3.68	0.10
083	1.91	5.05	2.39	2.23	2.36	4.90	5.97	3.75	0.18
080	1.81	5.68	2.37	2.22	2.32	4.41	5.81	3.27	0.15
053	1.91	5.40	2.35	2.25	2.37	4.69	6.60	4.15	0.26
004	1.96	5.23	2.38	2.25	2.36	4.77	6.37	3.70	0.29
000	2.16	5.22	2.38	2.25	2.36	4.78	6.39	3.71	0.00

Table S39. Properties of $(La_2Ce_2O_7)_6$ nanoclusters. Relative energy per atom, ΔE_{tot} (meV), HOMO-LUMO gap, E_g (eV), average radius of nanocluster, R_{av} (eV), average bond length distance of La, Ce and O, d_{av}^{La}, d_{av}^{Ce} and d_{av}^{O} (Å), respectively, average effective coordination number of La, Ce and O, $\mathrm{ECN}_{av}^{\mathrm{La}}$, $\mathrm{ECN}_{av}^{\mathrm{Ce}}$ and $\mathrm{ECN}_{av}^{\mathrm{O}}$ (NNN), respectively, and ratio of internal atoms to those exposed to the vacuum, n^{core}/n^{surf}.

ΔE_{tot}	E_g	R_{av}	d_{av}^{La}	d_{av}^{Ce}	d_{av}^{O}	$\mathrm{ECN}_{av}^{\mathrm{La}}$	$\mathrm{ECN}_{av}^{\mathrm{Ce}}$	$\mathrm{ECN}_{av}^{\mathrm{O}}$	$\frac{n^{core}}{n^{surf}}$
53	2.01	6.97	2.35	2.18	2.28	4.18	5.15	2.90	0.14
47	1.80	6.61	2.37	2.19	2.31	4.54	5.37	3.16	0.10
44	1.91	6.71	2.36	2.20	2.30	4.27	5.39	2.99	0.08
43	1.76	6.92	2.35	2.23	2.32	4.20	5.84	3.22	0.12
40	1.93	6.17	2.39	2.21	2.34	4.62	5.80	3.43	0.08
40	1.62	6.82	2.37	2.24	2.36	4.53	6.27	3.70	0.10
38	1.99	6.77	2.41	2.20	2.34	4.72	5.62	3.31	0.10
34	1.59	7.55	2.36	2.23	2.34	4.64	6.05	3.65	0.06
33	1.61	7.17	2.39	2.20	2.32	4.69	5.57	3.31	0.12
32	2.04	6.57	2.40	2.19	2.33	4.80	5.51	3.36	0.10
32	1.79	6.92	2.35	2.21	2.31	4.36	5.67	3.20	0.14
28	1.46	6.85	2.38	2.21	2.33	4.53	5.56	3.18	0.14
28	1.87	6.69	2.36	2.21	2.31	4.25	5.60	3.14	0.10
25	1.85	6.52	2.35	2.23	2.33	4.50	5.80	3.29	0.16
24	1.53	6.42	2.37	2.21	2.32	4.50	5.66	3.25	0.05
22	1.74	6.50	2.38	2.23	2.36	4.70	6.20	3.75	0.10
21	1.72	7.08	2.38	2.20	2.32	4.44	5.65	3.21	0.12
21	1.98	7.58	2.37	2.22	2.33	4.46	5.82	3.35	0.16
21	1.79	6.98	2.40	2.20	2.34	4.74	5.63	3.33	0.12
17	1.91	6.60	2.37	2.20	2.32	4.55	5.54	3.20	0.14
16	1.36	7.01	2.39	2.24	2.36	4.64	6.16	3.60	0.08
14	1.88	6.80	2.37	2.23	2.35	4.65	6.00	3.56	0.06
14	1.86	6.24	2.37	2.23	2.35	4.70	5.95	3.52	0.06
14	1.84	7.23	2.38	2.24	2.34	4.55	6.01	3.45	0.08
13	1.70	6.42	2.38	2.24	2.36	4.73	6.08	3.62	0.18
12	1.69	7.00	2.39	2.23	2.35	4.77	5.97	3.65	0.12
12	2.09	6.97	2.38	2.21	2.33	4.53	5.83	3.36	0.08
7	2.04	6.17	2.38	2.22	2.34	4.68	5.88	3.48	0.08
7	1.97	7.00	2.39	2.22	2.34	4.70	5.91	3.47	0.10
0	2.08	7.01	2.40	2.22	2.34	4.79	5.81	3.44	0.10

Table S40. Properties of $(La_2Ce_2O_7)_8$ nanoclusters. Relative energy per atom, ΔE_{tot} (meV), HOMO-LUMO gap, E_g (eV), average radius of nanocluster, R_{av} (eV), average bond length distance of La, Ce and O, d_{av}^{La}, d_{av}^{Ce} and d_{av}^{O} (Å), respectively, average effective coordination number of La, Ce and O, $\mathrm{ECN}_{av}^{\mathrm{La}}$, $\mathrm{ECN}_{av}^{\mathrm{Ce}}$ and $\mathrm{ECN}_{av}^{\mathrm{O}}$ (NNN), respectively, and ratio of internal atoms to those exposed to the vacuum, n^{core}/n^{surf}.

ΔE_{tot}	E_g	R_{av}	d_{av}^{La}	d_{av}^{Ce}	d_{av}^{O}	$\mathrm{ECN}_{av}^{\mathrm{La}}$	$\mathrm{ECN}_{av}^{\mathrm{Ce}}$	$\mathrm{ECN}_{av}^{\mathrm{O}}$	$\frac{n^{core}}{n^{surf}}$
72	1.79	7.77	2.37	2.21	2.32	4.50	5.57	3.21	0.09
65	1.83	7.64	2.38	2.22	2.34	4.57	5.87	3.41	0.10
64	1.48	7.89	2.37	2.22	2.34	4.42	6.02	3.46	0.13
63	1.92	7.37	2.39	2.23	2.35	4.62	6.13	3.57	0.19
63	1.64	7.62	2.37	2.23	2.35	4.66	5.97	3.52	0.19
62	1.74	8.57	2.35	2.24	2.34	4.50	6.04	3.49	0.11
61	1.63	7.51	2.37	2.23	2.35	4.73	5.95	3.57	0.11
59	1.81	7.79	2.34	2.23	2.32	4.28	5.98	3.36	0.04
57	1.84	7.66	2.39	2.20	2.32	4.55	5.54	3.15	0.06
57	1.75	8.05	2.37	2.22	2.33	4.47	5.78	3.24	0.14
55	1.58	7.97	2.38	2.23	2.35	4.66	5.96	3.46	0.16
54	2.04	7.25	2.34	2.23	2.33	4.46	5.95	3.40	0.11
53	1.67	7.80	2.39	2.22	2.35	4.72	5.99	3.58	0.13
51	1.59	7.52	2.40	2.21	2.34	4.79	5.72	3.44	0.13
51	1.68	7.29	2.39	2.24	2.36	4.90	6.20	3.79	0.16
49	1.53	8.06	2.38	2.22	2.33	4.49	5.83	3.29	0.14
49	1.60	7.54	2.39	2.23	2.36	4.63	6.08	3.53	0.19
45	1.59	7.10	2.39	2.26	2.38	4.80	6.60	4.01	0.16
44	1.66	7.42	2.39	2.21	2.33	4.57	5.72	3.29	0.13
43	1.70	7.22	2.36	2.23	2.34	4.50	6.03	3.44	0.10
42	1.60	8.09	2.38	2.24	2.35	4.65	6.05	3.55	0.13
42	1.73	7.85	2.37	2.24	2.35	4.61	6.29	3.66	0.13
40	1.76	7.90	2.39	2.22	2.35	4.69	5.98	3.52	0.14
40	1.97	7.73	2.38	2.23	2.35	4.65	6.05	3.51	0.16
39	1.87	7.46	2.39	2.24	2.36	4.83	6.06	3.61	0.16
38	1.67	7.40	2.39	2.23	2.35	4.93	5.98	3.67	0.16
37	1.72	7.81	2.39	2.23	2.35	4.66	6.04	3.48	0.16
37	1.54	7.55	2.39	2.25	2.38	4.97	6.27	3.84	0.14
36	1.78	8.10	2.39	2.24	2.37	4.90	6.19	3.77	0.16
35	1.72	8.11	2.39	2.24	2.37	4.89	6.25	3.79	0.14
33	1.70	6.95	2.41	2.24	2.38	5.07	6.27	3.91	0.14
33	1.66	7.19	2.38	2.24	2.37	4.84	6.19	3.72	0.19
33	2.03	7.25	2.40	2.23	2.36	4.88	6.08	3.67	0.19
31	1.61	7.39	2.39	2.24	2.36	4.91	6.11	3.68	0.22
31	2.05	7.33	2.41	2.22	2.37	4.95	6.12	3.79	0.17
31	1.78	7.34	2.40	2.22	2.37	5.05	6.01	3.76	0.17
29	1.59	7.45	2.39	2.24	2.37	5.02	6.10	3.75	0.19
28	1.80	7.54	2.39	2.24	2.37	5.13	6.21	3.94	0.07
23	2.07	7.30	2.38	2.24	2.36	4.82	6.13	3.64	0.17
0	1.99	7.55	2.40	2.24	2.37	5.10	6.14	3.75	0.19

Table S41. Properties of $(La_2Ce_2O_7)_{10}$ nanoclusters. Relative energy per atom, ΔE_{tot} (meV), HOMO-LUMO gap, E_g (eV), average radius of nanocluster, R_{av} (eV), average bond length distance of La, Ce and O, d_{av}^{La}, d_{av}^{Ce} and d_{av}^{O} (Å), respectively, average effective coordination number of La, Ce and O, $\mathrm{ECN}_{av}^{\mathrm{La}}$, $\mathrm{ECN}_{av}^{\mathrm{Ce}}$ and $\mathrm{ECN}_{av}^{\mathrm{O}}$ (NNN), respectively, and ratio of internal atoms to those exposed to the vacuum, n^{core}/n^{surf}.

ΔE_{tot}	E_g	R_{av}	d_{av}^{La}	d_{av}^{Ti}	d_{av}^{O}	$\mathrm{ECN}_{av}^{\mathrm{La}}$	$\mathrm{ECN}_{av}^{\mathrm{Ti}}$	$\mathrm{ECN}_{av}^{\mathrm{O}}$	$\frac{n^{core}}{n^{surf}}$
55	1.19	8.31	2.39	2.21	2.33	4.59	5.67	3.25	0.17
51	1.56	8.18	2.37	2.23	2.34	4.47	6.05	3.47	0.20
50	1.61	8.04	2.39	2.23	2.35	4.59	6.21	3.63	0.11
50	1.45	8.16	2.38	2.23	2.34	4.68	5.95	3.49	0.17
48	1.61	7.69	2.38	2.21	2.33	4.53	5.80	3.35	0.21
47	1.60	7.95	2.38	2.25	2.37	4.71	6.32	3.76	0.20
47	1.46	8.37	2.39	2.21	2.34	4.69	5.77	3.40	0.21
46	1.87	8.51	2.39	2.21	2.33	4.54	5.67	3.24	0.22
45	1.68	9.09	2.39	2.21	2.34	4.72	5.77	3.42	0.26
38	1.57	8.67	2.40	2.23	2.36	4.91	5.97	3.58	0.17
37	1.73	8.15	2.39	2.21	2.33	4.70	5.71	3.34	0.21
36	1.63	7.85	2.40	2.22	2.35	4.91	5.80	3.49	0.18
35	1.77	8.67	2.38	2.23	2.34	4.79	5.78	3.39	0.24
35	1.82	8.52	2.39	2.22	2.35	4.84	5.94	3.61	0.24
34	1.67	7.86	2.39	2.24	2.37	4.81	6.28	3.74	0.15
34	1.54	8.85	2.38	2.25	2.36	4.81	6.23	3.74	0.11
34	1.52	9.16	2.38	2.24	2.36	4.95	6.06	3.69	0.22
34	1.44	9.71	2.39	2.22	2.34	4.91	5.70	3.42	0.21
34	1.39	7.81	2.39	2.23	2.35	4.67	6.05	3.51	0.24
33	1.76	7.72	2.40	2.24	2.37	4.93	6.30	3.79	0.11
33	1.64	8.26	2.39	2.24	2.37	4.94	6.16	3.74	0.24
32	1.45	7.86	2.39	2.24	2.36	4.89	6.16	3.72	0.21
32	1.58	8.05	2.41	2.23	2.37	4.95	6.20	3.78	0.22
32	2.02	7.98	2.39	2.22	2.34	4.79	5.89	3.53	0.16
30	1.88	8.35	2.38	2.25	2.37	4.73	6.37	3.74	0.20
29	1.69	7.59	2.39	2.25	2.37	5.00	6.23	3.79	0.16
28	1.61	8.00	2.37	2.24	2.35	4.60	6.18	3.52	0.17
27	1.86	8.62	2.42	2.22	2.36	5.02	5.96	3.62	0.21
25	1.75	8.51	2.39	2.23	2.35	4.63	6.17	3.53	0.20
25	1.91	8.05	2.41	2.23	2.36	4.95	6.00	3.63	0.18
22	1.83	7.67	2.39	2.24	2.37	4.96	6.10	3.66	0.24
22	1.69	8.33	2.39	2.25	2.38	4.89	6.38	3.86	0.21
21	1.55	8.20	2.41	2.24	2.37	4.98	6.16	3.74	0.20
21	1.70	7.86	2.41	2.23	2.37	5.06	6.08	3.73	0.12
21	1.67	7.69	2.39	2.24	2.36	4.74	6.23	3.62	0.16

Table S42. Continuation of Table S41

ΔE_{tot}	E_g	R_{av}	d_{av}^{La}	d_{av}^{Ce}	d_{av}^{O}	$\mathrm{ECN}_{av}^{\mathrm{La}}$	$\mathrm{ECN}_{av}^{\mathrm{Ce}}$	$\mathrm{ECN}_{av}^{\mathrm{O}}$	$\frac{n^{core}}{n^{surf}}$
20	1.97	7.86	2.39	2.24	2.36	4.81	6.11	3.61	0.18
19	1.90	8.19	2.39	2.23	2.36	4.87	5.99	3.57	0.09
19	1.56	8.17	2.39	2.26	2.38	4.87	6.55	3.96	0.18
18	1.74	8.51	2.41	2.22	2.37	5.17	5.97	3.71	0.13
18	1.79	8.01	2.38	2.23	2.35	4.84	6.02	3.59	0.20
17	1.67	8.30	2.39	2.24	2.38	4.97	6.32	3.83	0.17
16	1.71	7.82	2.40	2.24	2.37	4.88	6.24	3.75	0.24
15	1.61	8.01	2.40	2.25	2.39	5.00	6.53	4.00	0.17
13	1.77	8.09	2.39	2.24	2.36	4.88	6.14	3.69	0.16
11	1.86	7.96	2.41	2.24	2.37	4.88	6.26	3.72	0.22
8	1.65	8.60	2.41	2.24	2.39	5.22	6.34	3.99	0.24
7	1.74	8.70	2.41	2.23	2.37	5.13	6.00	3.65	0.18
3	1.76	7.82	2.42	2.24	2.39	5.06	6.45	3.94	0.16
3	1.75	7.83	2.42	2.24	2.39	5.07	6.44	3.94	0.18
0	1.87	7.71	2.40	2.25	2.38	4.90	6.45	3.86	0.26

S4 ADDITIONAL RESULTS FOR LOWEST ENERGY NANOCLUSTERS CONFIGURATION

To understand the energetic stability of the studied nanoclusters, we investigated the most stable structures of each set. This section contains additional results, which support the graphs and figures presented in the article.

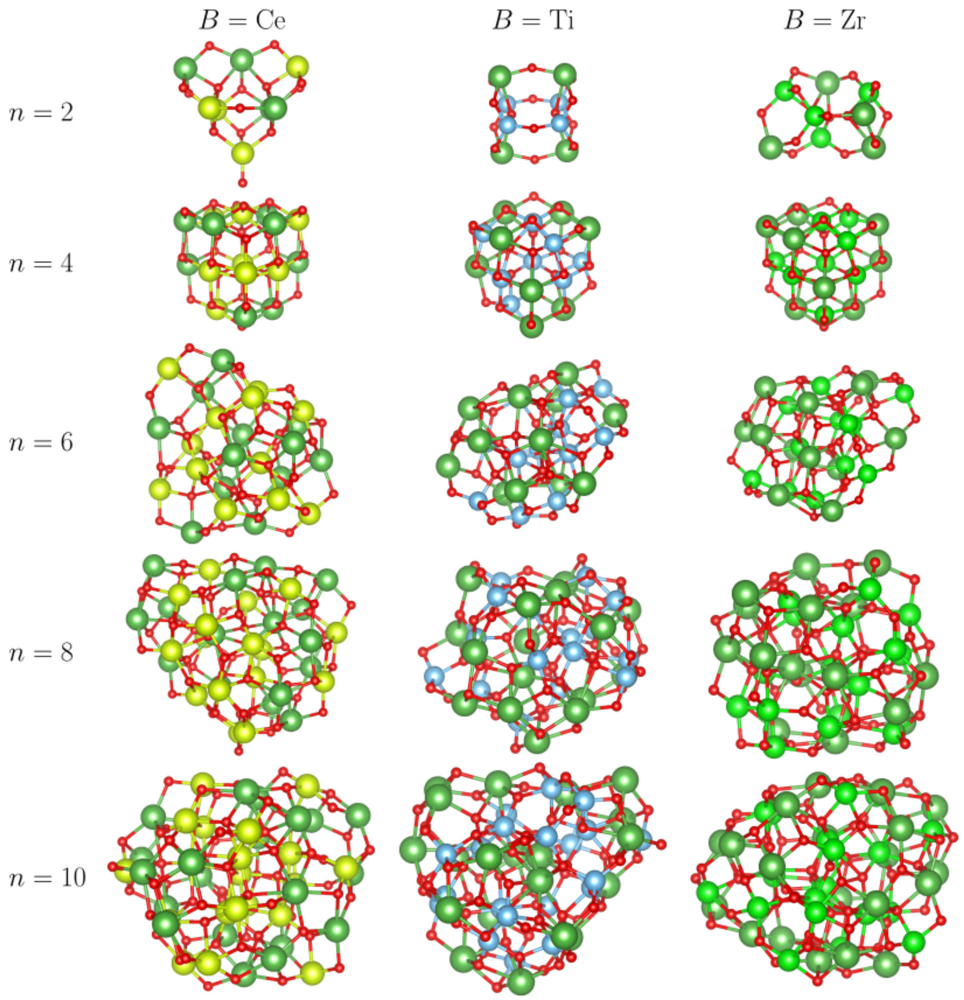

Figure S9. Lowest energy configurations of $(La_2B_2O_7)_n$ nanoclusters with B = Ti, Zr and Ce.

S4.1 Binding Energy

Table S43. Binding energy per atom, E_b, in eV, calculated for each $(La_2B_2O_7)_n$.

B/n	Ti	Zr	Ce
2	−6.28	−6.44	−6.21
4	−6.46	−6.72	−6.46
6	−6.48	−6.71	−6.44
8	−6.52	−6.75	−6.50
10	−6.55	−6.78	−6.51

S4.2 Electrostatic Potential Mapping

The electrostatic potential mapping (MEP) was calculated through the Visual Molecular Dynamics (VMD)[1] software, using particle-mesh Ewald method (PME),[2] where every

point is approximated by a spherical Gaussian:

$$\rho_i(\mathbf{r}) = q_i \left(\frac{\beta}{\sqrt{\pi}}\right)^3 e^{-\beta^2|\mathbf{r}-\mathbf{r}_i|^2}, \tag{S2}$$

where q is the charge at point r and β determines the width of the Gaussian. Then, electrostatic potential value, $\phi(\mathbf{r})$ is obtained by solving the Poisson equation, considering the sum over all atoms of the nanocluster:

$$\nabla^2\phi(\mathbf{r}) = 4\pi \sum_i \rho(\mathbf{r}). \tag{S3}$$

The FFT algorithm was used to describe the long range interactions in the reciprocal space, while the short range interactions are calculated in the direct space.

S5 ADDITIONAL EFFECTS OF O-VACANCY FORMATION

We investigated the conditions for O vacancy formation of $(La_2Zr_2O_7)_n$, $(La_2Ce_2O_7)_n$ and $(La_2Ti_2O_7)_n$ most stable configurations. Then, this section contains results complementary to those presented in the article, in addition to data used in the reproduction of tables and graphs.

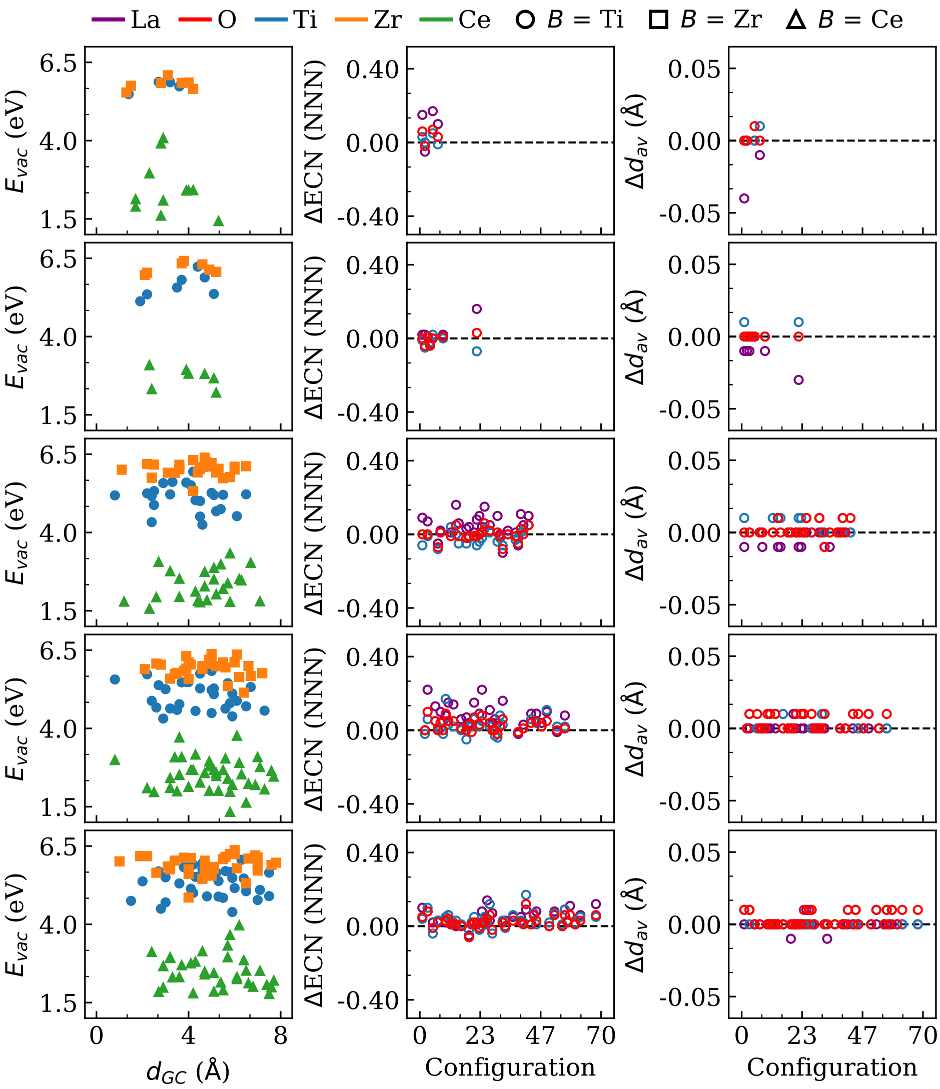

Figure S10. Vacancy energy, E_{vac}, versus the initial position of the oxygen vacancy with respect to the geometric center, d_{GC}, of the $(La_2B_2O_7)_n$ nanocluster, with B = Ti, Zr and Ce and n = 2, 4, 6, 8 and 10. Variation of effective coordination number (ΔECN) in the $(La_2Ti_2O_7)_n$ nanocluster, with n = 2, 4, 6, 8 and 10, resulted from the vacancy formation. Variation of Average bond distance (Δd_{av}) in the$(La_2Ti_2O_7)_n$nanocluster, with n = 2, 4, 6, 8 and 10, resulted from the vacancy formation.

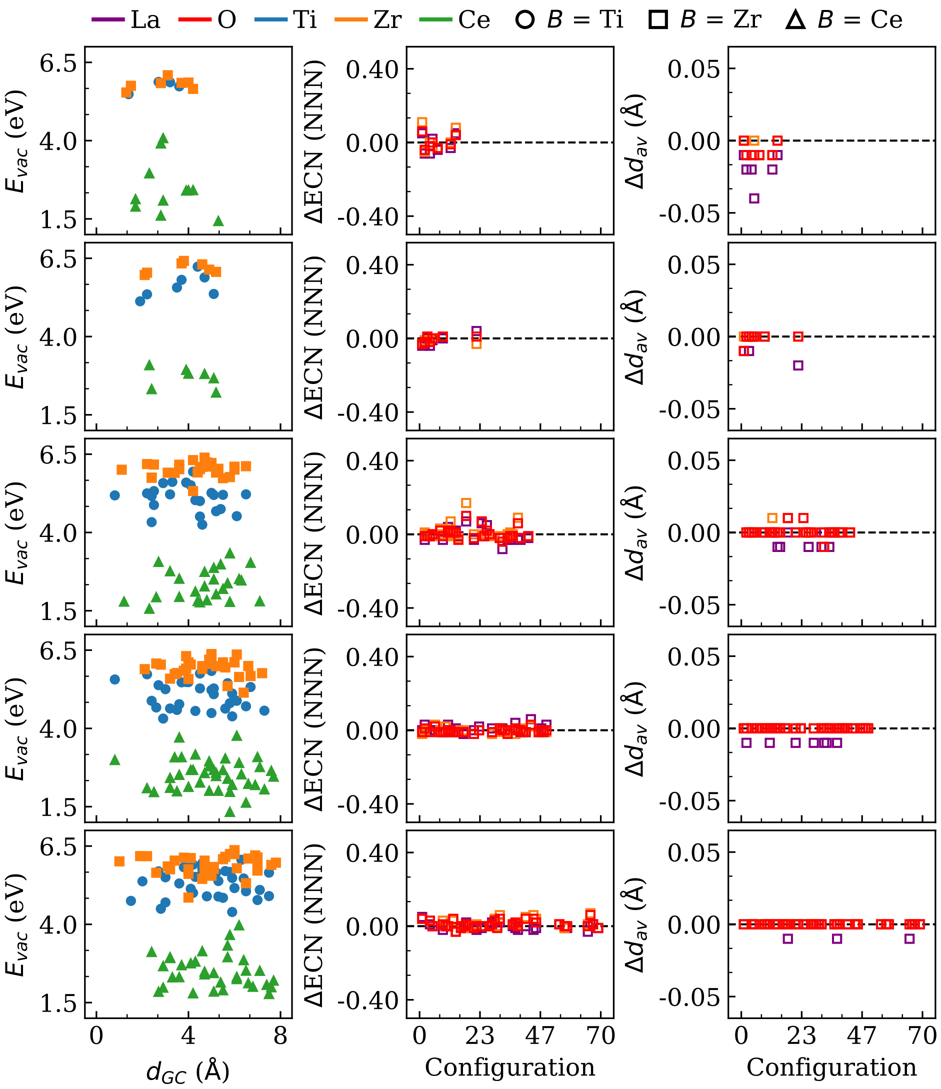

Figure S11. Vacancy energy, E_{vac}, versus the initial position of the oxygen vacancy with respect to the geometric center, d_{GC}, of the $(La_2B_2O_7)_n$ nanocluster, with B = Ti, Zr and Ce and n = 2, 4, 6, 8 and 10. Variation of effective coordination number (ΔECN) in the $(La_2Zr_2O_7)_n$ nanocluster, with n = 2, 4, 6, 8 and 10, resulted from the vacancy formation. Variation of Average bond distance (Δd_{av}) in the$(La_2Zr_2O_7)_n$nanocluster, with n = 2, 4, 6, 8 and 10, resulted from the vacancy formation.

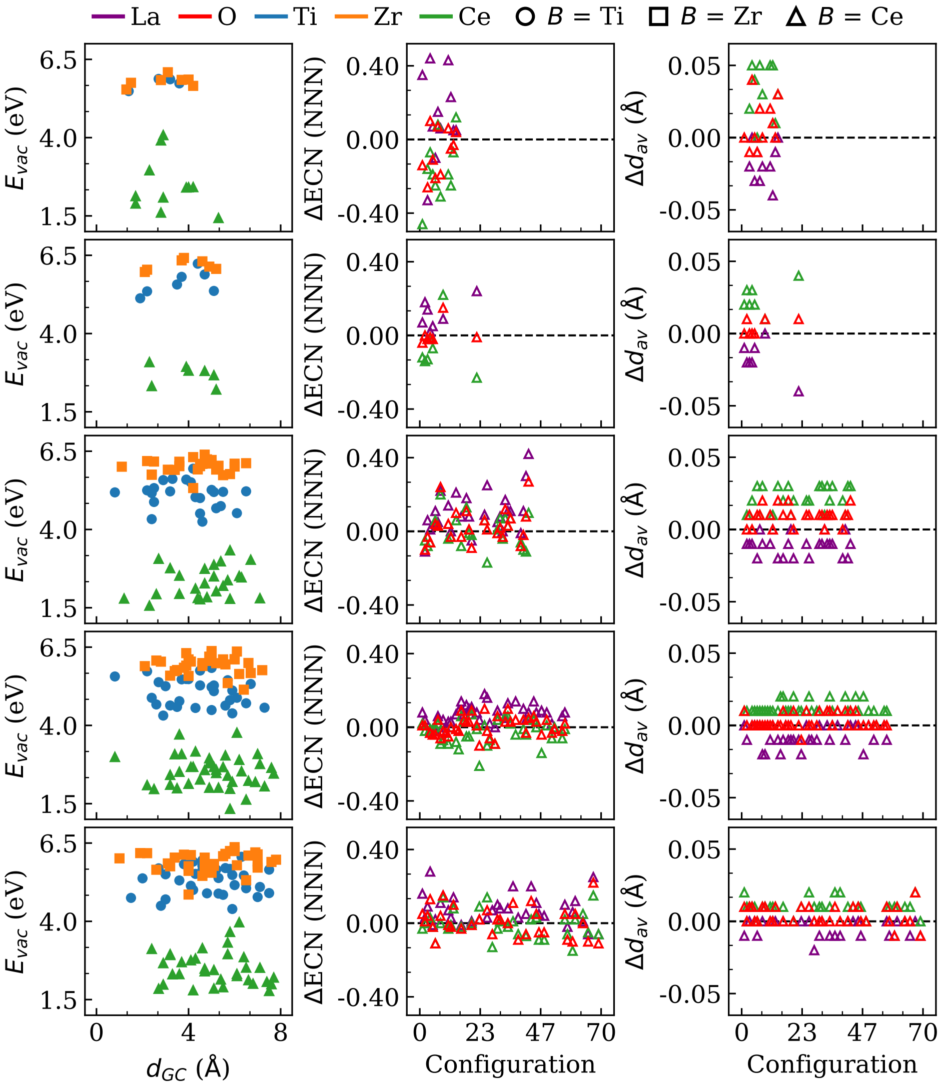

Figure S12. Vacancy energy, E_{vac}, versus the initial position of the oxygen vacancy with respect to the geometric center, d_{GC}, of the $(La_2B_2O_7)_n$ nanocluster, with B = Ti, Zr and Ce and n = 2, 4, 6, 8 and 10. Variation of effective coordination number (ΔECN) in the $(La_2Ce_2O_7)_n$ nanocluster, with n = 2, 4, 6, 8 and 10, resulted from the vacancy formation. Variation of Average bond distance (Δd_{av}) in the$(La_2Ce_2O_7)_n$nanocluster, with n = 2, 4, 6, 8 and 10, resulted from the vacancy formation.

S5.1 Additional Features for Defective Structures

Table S44. Energetic, structural, and electronic properties of $(La_2Ti_2O_7)_2$ nanoclusters subjected to a single O-vacancy formation include the following parameters: vacancy energy (E_{vac} in eV), distance of the vacancy from the nanoclusters center of gravity (d_{vac}^{gc} in Å), total magnetic moment (m_{tot} ,in μ_B), average bond length distances for La, Ti, and O (d_{av}^{La}, d_{av}^{B}, and d_{av}^{O} in Å, respectively), average effective coordination numbers for La, Ti, and O ($\mathrm{ECN}_{av}^{\mathrm{La}}$, $\mathrm{ECN}_{av}^{\mathrm{B}}$, and $\mathrm{ECN}_{av}^{\mathrm{O}}$ in NNN, respectively).

E_{vac}	d_{vac}^{gc}	m_{tot}	d_{av}^{La}	d_{av}^{B}	d_{av}^{O}	$\mathrm{ECN}_{av}^{\mathrm{La}}$	$\mathrm{ECN}_{av}^{\mathrm{B}}$	$\mathrm{ECN}_{av}^{\mathrm{O}}$
5.88	2.7	0.0	2.22	1.83	1.96	2.07	3.91	1.84
5.86	3.2	0.0	2.21	1.84	1.95	1.81	4.47	1.94
5.73	3.6	0.0	2.21	1.85	1.97	2.14	4.16	1.94
5.48	1.4	0.0	2.20	1.83	1.96	2.20	3.91	1.88

Table S45. Energetic, structural, and electronic properties of $(La_2Ti_2O_7)_4$ nanoclusters subjected to a single O-vacancy formation include the following parameters: vacancy energy (E_{vac} in eV), distance of the vacancy from the nanoclusters center of gravity (d_{vac}^{gc} in Å), total magnetic moment (m_{tot} ,in μ_B), average bond length distances for La, Ti, and O (d_{av}^{La}, d_{av}^{B}, and d_{av}^{O} in Å, respectively), average effective coordination numbers for La, Ti, and O ($\mathrm{ECN}_{av}^{\mathrm{La}}$, $\mathrm{ECN}_{av}^{\mathrm{B}}$, and $\mathrm{ECN}_{av}^{\mathrm{O}}$ in NNN, respectively).

E_{vac}	d_{vac}^{cg}	m_{tot}	d_{av}^{La}	d_{av}^{B}	d_{av}^{O}	$\mathrm{ECN}_{av}^{\mathrm{La}}$	$\mathrm{ECN}_{av}^{\mathrm{B}}$	$\mathrm{ECN}_{av}^{\mathrm{O}}$
6.23	4.4	0.0	2.36	1.96	2.13	3.25	6.55	3.18
5.89	4.7	2.0	2.36	1.97	2.14	3.38	6.55	3.22
5.81	3.7	0.0	2.35	1.96	2.12	3.25	6.47	3.11
5.57	3.5	0.0	2.36	1.96	2.12	3.29	6.37	3.10
5.36	5.1	2.0	2.35	1.97	2.13	3.40	6.67	3.29
5.35	2.2	0.0	2.35	1.95	2.12	3.21	6.35	3.07
5.13	1.9	0.0	2.36	1.95	2.12	3.30	6.25	3.05

Table S46. Energetic, structural, and electronic properties of $(La_2Ti_2O_7)_6$ nanoclusters subjected to a single O-vacancy formation include the following parameters: vacancy energy (E_{vac} in eV), distance of the vacancy from the nanoclusters center of gravity (d^{gc}_{vac} in Å), total magnetic moment (m_{tot} ,in μ_B), average bond length distances for La, Ti, and O (d^{La}_{av}, d^{B}_{av}, and d^{O}_{av} in Å, respectively), average effective coordination numbers for La, Ti, and O ($\mathrm{ECN}^{\mathrm{La}}_{av}$, $\mathrm{ECN}^{\mathrm{B}}_{av}$, and $\mathrm{ECN}^{\mathrm{O}}_{av}$ in NNN, respectively).

E_{vac}	d^{cg}_{vac}	m_{tot}	d^{La}_{av}	d^{B}_{av}	d^{O}_{av}	$\mathrm{ECN}^{\mathrm{La}}_{av}$	$\mathrm{ECN}^{\mathrm{B}}_{av}$	$\mathrm{ECN}^{\mathrm{O}}_{av}$
5.94	4.2	0.0	2.36	1.92	2.09	3.29	5.82	2.84
5.94	4.4	0.0	2.36	1.92	2.09	3.35	5.73	2.82
5.62	3.3	0.0	2.36	1.92	2.09	3.33	5.71	2.80
5.59	3.9	0.0	2.36	1.92	2.09	3.35	5.69	2.80
5.58	2.9	0.0	2.36	1.92	2.09	3.36	5.72	2.81
5.51	4.1	0.0	2.36	1.92	2.09	3.33	5.73	2.82
5.32	2.5	0.0	2.36	1.92	2.09	3.33	5.69	2.77
5.26	5.0	2.0	2.37	1.93	2.10	3.44	5.85	2.89
5.25	2.2	0.0	2.37	1.93	2.09	3.27	5.83	2.81
5.22	6.5	0.0	2.36	1.93	2.10	3.43	5.80	2.87
5.22	3.2	0.0	2.36	1.91	2.09	3.31	5.62	2.76
5.20	5.5	0.0	2.36	1.93	2.10	3.40	5.78	2.85
5.20	5.1	2.0	2.36	1.93	2.09	3.28	5.93	2.86
5.19	0.8	0.0	2.36	1.92	2.09	3.34	5.63	2.78
5.16	2.4	0.0	2.36	1.91	2.09	3.32	5.59	2.74
5.03	4.3	0.0	2.36	1.92	2.09	3.25	5.69	2.76
5.00	4.5	2.0	2.36	1.93	2.09	3.21	5.90	2.81
4.88	2.5	0.0	2.36	1.92	2.09	3.30	5.73	2.79
4.75	5.4	2.0	2.37	1.93	2.10	3.40	5.84	2.88
4.68	5.2	2.0	2.37	1.93	2.10	3.38	5.81	2.84
4.52	6.1	2.0	2.36	1.93	2.10	3.40	5.78	2.85
4.51	4.5	2.0	2.36	1.92	2.09	3.26	5.66	2.75
4.33	2.4	2.0	2.37	1.92	2.09	3.37	5.75	2.81
4.25	4.6	2.0	2.36	1.93	2.09	3.32	5.76	2.81

Table S47. Energetic, structural, and electronic properties of $(La_2Ti_2O_7)_8$ nanoclusters subjected to a single O-vacancy formation include the following parameters: vacancy energy (E_{vac} in eV), distance of the vacancy from the nanoclusters center of gravity (d_{vac}^{gc} in Å), total magnetic moment (m_{tot} ,in μ_B), average bond length distances for La, Ti, and O (d_{av}^{La}, d_{av}^{B}, and d_{av}^{O} in Å, respectively), average effective coordination numbers for La, Ti, and O ($\mathrm{ECN}_{av}^{\mathrm{La}}$, $\mathrm{ECN}_{av}^{\mathrm{B}}$, and $\mathrm{ECN}_{av}^{\mathrm{O}}$ in NNN, respectively).

E_{vac}	d_{vac}^{cg}	m_{tot}	d_{av}^{La}	d_{av}^{B}	d_{av}^{O}	$\mathrm{ECN}_{av}^{\mathrm{La}}$	$\mathrm{ECN}_{av}^{\mathrm{B}}$	$\mathrm{ECN}_{av}^{\mathrm{O}}$
5.84	5.0	0.0	2.38	1.90	2.09	3.40	5.60	2.77
5.75	4.5	0.0	2.38	1.90	2.09	3.41	5.60	2.75
5.73	2.2	0.0	2.39	1.90	2.09	3.44	5.58	2.76
5.56	0.8	0.0	2.39	1.90	2.09	3.48	5.51	2.75
5.48	3.9	0.0	2.39	1.90	2.09	3.47	5.63	2.79
5.48	4.0	0.0	2.38	1.90	2.09	3.47	5.55	2.76
5.47	3.7	0.0	2.38	1.90	2.09	3.40	5.54	2.73
5.44	5.7	2.0	2.39	1.90	2.10	3.55	5.65	2.82
5.38	2.7	0.0	2.39	1.90	2.09	3.46	5.56	2.76
5.32	6.7	0.0	2.39	1.90	2.10	3.50	5.60	2.79
5.28	4.5	2.0	2.38	1.91	2.09	3.34	5.72	2.76
5.28	5.1	0.0	2.39	1.91	2.09	3.43	5.73	2.81
5.25	3.0	0.0	2.38	1.90	2.09	3.47	5.56	2.75
5.23	5.0	0.0	2.38	1.91	2.09	3.47	5.62	2.78
5.12	5.9	0.0	2.38	1.91	2.09	3.42	5.61	2.76
5.09	5.1	0.0	2.39	1.91	2.10	3.48	5.65	2.79
4.88	6.1	0.0	2.39	1.91	2.10	3.51	5.65	2.79
4.88	2.4	2.0	2.39	1.91	2.10	3.53	5.62	2.80
4.81	5.8	0.0	2.39	1.90	2.09	3.47	5.52	2.74
4.77	3.6	0.0	2.39	1.90	2.09	3.42	5.62	2.75
4.71	6.5	0.0	2.39	1.91	2.11	3.64	5.68	2.88
4.67	2.6	0.0	2.39	1.91	2.10	3.55	5.65	2.81
4.64	3.2	0.0	2.38	1.91	2.10	3.55	5.67	2.81
4.63	5.6	2.0	2.38	1.91	2.09	3.43	5.75	2.82
4.59	3.5	0.0	2.39	1.90	2.09	3.47	5.52	2.74
4.56	7.3	0.0	2.39	1.90	2.10	3.50	5.57	2.77
4.56	4.3	0.0	2.39	1.90	2.10	3.56	5.60	2.81
4.49	5.0	0.0	2.38	1.90	2.09	3.39	5.54	2.73
4.39	5.9	0.0	2.39	1.90	2.10	3.49	5.60	2.76
4.31	2.9	2.0	2.38	1.91	2.09	3.50	5.65	2.80

Table S48. Energetic, structural, and electronic properties of $(La_2Ti_2O_7)_{10}$ nanoclusters subjected to a single O-vacancy formation include the following parameters: vacancy energy (E_{vac} in eV), distance of the vacancy from the nanoclusters center of gravity (d_{vac}^{gc} in Å), total magnetic moment (m_{tot} ,in μ_B), average bond length distances for La, Ti, and O (d_{av}^{La}, d_{av}^{B}, and d_{av}^{O} in Å, respectively), average effective coordination numbers for La, Ti, and O ($\mathrm{ECN}_{av}^{\mathrm{La}}$, $\mathrm{ECN}_{av}^{\mathrm{B}}$, and $\mathrm{ECN}_{av}^{\mathrm{O}}$ in NNN, respectively).

E_{vac}	d_{vac}^{cg}	m_{tot}	d_{av}^{La}	d_{av}^{B}	d_{av}^{O}	$\mathrm{ECN}_{av}^{\mathrm{La}}$	$\mathrm{ECN}_{av}^{\mathrm{B}}$	$\mathrm{ECN}_{av}^{\mathrm{O}}$
6.06	6.3	0.0	2.39	1.92	2.11	3.56	5.87	2.94
5.95	4.6	0.0	2.39	1.92	2.11	3.56	5.95	2.98
5.91	4.2	0.0	2.39	1.92	2.11	3.60	5.87	2.94
5.86	3.9	0.0	2.39	1.91	2.10	3.47	5.83	2.88
5.83	3.8	0.0	2.39	1.91	2.11	3.58	5.81	2.92
5.78	7.0	0.0	2.39	1.92	2.10	3.45	5.95	2.92
5.78	4.6	0.0	2.39	1.92	2.11	3.54	5.86	2.93
5.7	5.6	0.0	2.39	1.92	2.11	3.51	5.93	2.94
5.69	2.7	0.0	2.38	1.92	2.10	3.51	5.81	2.89
5.67	5.8	0.0	2.39	1.92	2.11	3.57	5.88	2.95
5.65	5.2	0.0	2.39	1.92	2.11	3.59	5.98	3.00
5.65	5.2	0.0	2.39	1.91	2.10	3.54	5.85	2.92
5.65	7.5	0.0	2.39	1.92	2.10	3.48	5.90	2.91
5.63	4.6	0.0	2.39	1.92	2.11	3.56	5.88	2.94
5.62	4.6	0.0	2.39	1.91	2.10	3.50	5.85	2.90
5.52	4.3	0.0	2.39	1.92	2.11	3.55	5.91	2.94
5.50	3.0	0.0	2.39	1.92	2.10	3.52	5.82	2.90
5.48	5.9	0.0	2.38	1.92	2.10	3.51	5.84	2.88
5.46	6.4	0.0	2.39	1.92	2.11	3.56	5.92	2.96
5.38	5.3	0.0	2.39	1.92	2.11	3.54	5.91	2.94
5.37	2.0	0.0	2.39	1.92	2.11	3.55	5.85	2.92
5.31	6.5	2.0	2.39	1.92	2.11	3.59	5.94	2.96
5.31	3.6	0.0	2.39	1.92	2.11	3.57	5.86	2.94
5.19	6.5	2.0	2.39	1.92	2.11	3.60	5.94	2.97
5.16	6.0	0.0	2.39	1.92	2.11	3.63	5.9	2.95
5.13	4.1	0.0	2.40	1.92	2.11	3.56	5.96	2.96
5.10	7.1	0.0	2.39	1.92	2.12	3.66	5.95	3.00
5.06	6.5	0.0	2.39	1.92	2.11	3.51	6.00	2.97
5.00	4.2	0.0	2.39	1.92	2.11	3.57	5.92	2.96
4.90	7.5	0.0	2.39	1.92	2.11	3.63	5.89	2.96
4.89	4.8	0.0	2.39	1.91	2.11	3.56	5.85	2.91
4.89	5.3	0.0	2.39	1.91	2.10	3.48	5.84	2.87
4.85	5.5	0.0	2.39	1.92	2.11	3.61	5.85	2.92
4.78	7.0	2.0	2.39	1.92	2.11	3.56	5.95	2.96
4.75	1.5	2.0	2.39	1.92	2.12	3.63	5.95	2.99
4.70	3.0	0.0	2.38	1.92	2.11	3.57	5.83	2.90
4.50	2.8	2.0	2.39	1.93	2.12	3.65	6.03	3.05
4.39	5.9	0.0	2.39	1.92	2.11	3.52	5.86	2.89

Table S49. Energetic, structural, and electronic properties of $(La_2Zr_2O_7)_2$ nanoclusters subjected to a single O-vacancy formation include the following parameters: vacancy energy (E_{vac} in eV), distance of the vacancy from the nanoclusters center of gravity (d_{vac}^{gc} in Å), total magnetic moment (m_{tot} ,in μ_B), average bond length distances for La, Zr, and O (d_{av}^{La}, d_{av}^{B}, and d_{av}^{O} in Å, respectively), average effective coordination numbers for La, Zr, and O ($\mathrm{ECN}_{av}^{\mathrm{La}}$, $\mathrm{ECN}_{av}^{\mathrm{B}}$, and $\mathrm{ECN}_{av}^{\mathrm{O}}$ in NNN, respectively).

E_{vac}	d_{vac}^{cg}	m_{tot}	d_{av}^{La}	d_{av}^{B}	d_{av}^{O}	$\mathrm{ECN}_{av}^{\mathrm{La}}$	$\mathrm{ECN}_{av}^{\mathrm{B}}$	$\mathrm{ECN}_{av}^{\mathrm{O}}$
6.09	3.1	0.0	2.23	2.00	2.09	2.44	4.28	2.08
5.86	4.0	0.0	2.21	2.01	2.09	2.41	4.25	2.06
5.84	3.7	0.0	2.22	2.01	2.09	2.41	4.20	2.05
5.84	2.8	0.0	2.23	2.02	2.10	2.50	4.38	2.15
5.75	1.5	0.0	2.22	2.00	2.09	2.55	4.04	2.04
5.65	4.2	0.0	2.22	2.01	2.09	2.24	4.55	2.11
5.54	1.3	0.0	2.22	1.99	2.08	2.58	3.90	2.00

Table S50. Energetic, structural, and electronic properties of $(La_2Zr_2O_7)_4$ nanoclusters subjected to a single O-vacancy formation include the following parameters: vacancy energy (E_{vac} in eV), distance of the vacancy from the nanoclusters center of gravity (d_{vac}^{gc} in Å), total magnetic moment (m_{tot} ,in μ_B), average bond length distances for La, Zr, and O (d_{av}^{La}, d_{av}^{B}, and d_{av}^{O} in Å, respectively), average effective coordination numbers for La, Zr, and O ($\mathrm{ECN}_{av}^{\mathrm{La}}$, $\mathrm{ECN}_{av}^{\mathrm{B}}$, and $\mathrm{ECN}_{av}^{\mathrm{O}}$ in NNN, respectively).

E_{vac}	d_{vac}^{cg}	m_{tot}	d_{av}^{La}	d_{av}^{B}	d_{av}^{O}	$\mathrm{ECN}_{av}^{\mathrm{La}}$	$\mathrm{ECN}_{av}^{\mathrm{B}}$	$\mathrm{ECN}_{av}^{\mathrm{O}}$
6.42	3.8	0.0	2.38	2.10	2.25	3.81	6.48	3.37
6.34	3.7	0.0	2.38	2.10	2.25	3.92	6.34	3.35
6.31	4.6	0.0	2.37	2.10	2.25	3.86	6.50	3.43
6.14	4.9	0.0	2.38	2.11	2.25	3.94	6.49	3.45
6.07	5.2	2.0	2.38	2.10	2.25	3.91	6.65	3.50
6.04	2.2	0.0	2.37	2.10	2.24	3.82	6.32	3.31
5.97	2.1	0.0	2.38	2.10	2.24	3.93	6.18	3.29

Table S51. Energetic, structural, and electronic properties of $(La_2Zr_2O_7)_6$ nanoclusters subjected to a single O-vacancy formation include the following parameters: vacancy energy (E_{vac} in eV), distance of the vacancy from the nanoclusters center of gravity (d_{vac}^{gc} in Å), total magnetic moment (m_{tot} ,in μ_B), average bond length distances for La, Zr, and O (d_{av}^{La}, d_{av}^{B}, and d_{av}^{O} in Å, respectively), average effective coordination numbers for La, Zr, and O ($\mathrm{ECN}_{av}^{\mathrm{La}}$, $\mathrm{ECN}_{av}^{\mathrm{B}}$, and $\mathrm{ECN}_{av}^{\mathrm{O}}$ in NNN, respectively).

E_{vac}	d_{vac}^{cg}	m_{tot}	d_{av}^{La}	d_{av}^{B}	d_{av}^{O}	$\mathrm{ECN}_{av}^{\mathrm{La}}$	$\mathrm{ECN}_{av}^{\mathrm{B}}$	$\mathrm{ECN}_{av}^{\mathrm{O}}$
6.39	4.7	0.0	2.37	2.07	2.22	3.83	5.73	3.04
6.31	4.2	0.0	2.37	2.07	2.21	3.77	5.80	3.05
6.24	4.8	0.0	2.37	2.07	2.22	3.84	5.73	3.05
6.22	5.0	0.0	2.37	2.07	2.22	3.89	5.75	3.07
6.19	2.2	0.0	2.37	2.07	2.21	3.65	5.77	2.97
6.17	2.5	0.0	2.37	2.07	2.21	3.79	5.69	2.99
6.16	3.6	0.0	2.37	2.07	2.21	3.79	5.70	3.01
6.12	6.5	0.0	2.37	2.07	2.22	3.84	5.82	3.08
6.10	5.0	0.0	2.36	2.07	2.21	3.79	5.78	3.04
6.10	6.0	0.0	2.37	2.07	2.22	3.90	5.81	3.10
6.09	4.6	0.0	2.37	2.07	2.22	3.79	5.82	3.06
6.04	5.3	0.0	2.37	2.07	2.21	3.78	5.81	3.05
6.02	3.6	0.0	2.37	2.07	2.21	3.84	5.70	3.01
6.02	4.5	0.0	2.38	2.07	2.22	3.73	5.93	3.08
6.01	6.0	0.0	2.37	2.07	2.22	3.89	5.76	3.07
6.01	1.1	0.0	2.37	2.07	2.21	3.87	5.61	2.98
5.92	4.4	0.0	2.36	2.07	2.21	3.80	5.71	3.02
5.91	5.2	0.0	2.37	2.08	2.21	3.67	5.92	3.05
5.91	3.1	0.0	2.37	2.07	2.21	3.79	5.71	3.00
5.90	3.4	0.0	2.36	2.07	2.21	3.82	5.60	2.97
5.77	5.8	0.0	2.37	2.08	2.22	3.85	5.80	3.05
5.75	2.4	0.0	2.37	2.07	2.22	3.89	5.64	3.00
5.75	2.4	0.0	2.37	2.08	2.22	3.89	5.83	3.14
5.74	5.5	0.0	2.37	2.08	2.22	3.94	5.83	3.11
5.32	4.2	0.0	2.37	2.08	2.23	3.92	5.89	3.14

Table S52. Energetic, structural, and electronic properties of $(La_2Zr_2O_7)_8$ nanoclusters subjected to a single O-vacancy formation include the following parameters: vacancy energy (E_{vac} in eV), distance of the vacancy from the nanoclusters center of gravity (d_{vac}^{gc} in Å), total magnetic moment (m_{tot} ,in μ_B), average bond length distances for La, Zr, and O (d_{av}^{La}, d_{av}^{B}, and d_{av}^{O} in Å, respectively), average effective coordination numbers for La, Zr, and O ($\text{ECN}_{av}^{\text{La}}$, $\text{ECN}_{av}^{\text{B}}$, and $\text{ECN}_{av}^{\text{O}}$ in NNN, respectively).

E_{vac}	d_{vac}^{cg}	m_{tot}	d_{av}^{La}	d_{av}^{B}	d_{av}^{O}	$\text{ECN}_{av}^{\text{La}}$	$\text{ECN}_{av}^{\text{B}}$	$\text{ECN}_{av}^{\text{O}}$
6.38	5.0	0.0	2.38	2.07	2.23	3.97	5.94	3.17
6.36	6.1	0.0	2.38	2.07	2.23	4.00	5.97	3.20
6.31	3.9	0.0	2.38	2.07	2.23	3.91	5.92	3.15
6.30	5.0	0.0	2.38	2.07	2.23	3.97	5.95	3.17
6.20	4.9	0.0	2.38	2.07	2.23	3.87	6.10	3.20
6.11	6.0	0.0	2.38	2.07	2.23	3.93	6.00	3.18
6.11	6.0	0.0	2.38	2.07	2.23	3.97	6.02	3.21
6.11	5.5	0.0	2.38	2.07	2.23	3.91	6.00	3.18
6.11	4.0	0.0	2.38	2.07	2.23	3.87	6.02	3.16
6.07	2.6	0.0	2.38	2.07	2.23	3.94	5.93	3.15
6.06	5.0	0.0	2.38	2.08	2.23	3.96	6.01	3.21
6.04	4.1	0.0	2.38	2.07	2.23	3.91	5.90	3.12
6.04	2.8	0.0	2.38	2.07	2.23	3.96	5.88	3.12
5.99	6.6	0.0	2.38	2.07	2.23	3.96	6.02	3.20
5.99	4.6	0.0	2.38	2.07	2.23	3.96	5.95	3.18
5.99	5.2	0.0	2.38	2.07	2.23	3.92	6.02	3.19
5.95	5.6	0.0	2.38	2.07	2.23	3.95	5.96	3.17
5.91	3.9	0.0	2.38	2.07	2.23	3.94	5.85	3.08
5.90	4.6	0.0	2.38	2.07	2.23	4.00	5.96	3.19
5.89	2.1	0.0	2.38	2.07	2.23	3.96	5.93	3.15
5.84	3.8	0.0	2.38	2.07	2.23	3.88	5.98	3.14
5.77	3.5	0.0	2.38	2.07	2.23	3.94	5.93	3.14
5.76	7.2	0.0	2.38	2.07	2.23	3.96	6.02	3.20
5.73	3.4	0.0	2.38	2.07	2.23	3.95	5.96	3.18
5.67	6.7	0.0	2.38	2.07	2.23	3.83	6.10	3.19
5.64	6.2	0.0	2.37	2.07	2.23	3.84	6.09	3.18
5.59	3.2	0.0	2.38	2.08	2.23	3.99	5.92	3.17
5.58	4.0	0.0	2.38	2.07	2.23	3.97	5.90	3.15
5.35	5.7	0.0	2.37	2.07	2.22	3.81	6.06	3.15
5.14	6.4	0.0	2.38	2.07	2.23	3.97	6.09	3.23

Table S53. Energetic, structural, and electronic properties of $(La_2Zr_2O_7)_{10}$ nanoclusters subjected to a single O-vacancy formation include the following parameters: vacancy energy (E_{vac} in eV), distance of the vacancy from the nanoclusters center of gravity (d_{vac}^{gc} in Å), total magnetic moment (m_{tot} ,in μ_B), average bond length distances for La, Zr, and O (d_{av}^{La}, d_{av}^{B}, and d_{av}^{O} in Å, respectively), average effective coordination numbers for La, Zr, and O ($\mathrm{ECN}_{av}^{\mathrm{La}}$, $\mathrm{ECN}_{av}^{\mathrm{B}}$, and $\mathrm{ECN}_{av}^{\mathrm{O}}$ in NNN, respectively).

E_{vac}	d_{vac}^{cg}	m_{tot}	d_{av}^{La}	d_{av}^{B}	d_{av}^{O}	$\mathrm{ECN}_{av}^{\mathrm{La}}$	$\mathrm{ECN}_{av}^{\mathrm{B}}$	$\mathrm{ECN}_{av}^{\mathrm{O}}$
6.37	6.0	0.0	2.40	2.08	2.25	4.18	6.19	3.34
6.26	6.0	0.0	2.40	2.09	2.25	4.15	6.25	3.36
6.25	5.8	0.0	2.40	2.09	2.25	4.20	6.20	3.36
6.25	6.0	0.0	2.40	2.09	2.26	4.18	6.26	3.38
6.20	6.9	0.0	2.40	2.09	2.25	4.13	6.21	3.33
6.18	1.9	0.0	2.40	2.09	2.25	4.21	6.20	3.37
6.18	2.2	0.0	2.40	2.09	2.25	4.18	6.15	3.32
6.16	5.6	0.0	2.40	2.09	2.25	4.15	6.26	3.36
6.14	7.0	0.0	2.40	2.09	2.25	4.18	6.26	3.38
6.13	3.8	0.0	2.40	2.08	2.25	4.19	6.15	3.33
6.12	4.1	0.0	2.40	2.08	2.25	4.20	6.14	3.33
6.10	6.6	0.0	2.40	2.09	2.26	4.16	6.31	3.41
6.08	5.5	0.0	2.40	2.09	2.25	4.10	6.32	3.38
6.04	3.4	0.0	2.40	2.09	2.25	4.13	6.17	3.30
6.04	4.7	0.0	2.40	2.09	2.25	4.07	6.24	3.33
6.01	1.0	0.0	2.40	2.09	2.26	4.24	6.15	3.36
5.97	7.8	0.0	2.40	2.09	2.25	4.17	6.26	3.38
5.90	7.6	0.0	2.40	2.09	2.25	4.11	6.25	3.34
5.85	3.1	0.0	2.40	2.09	2.25	4.18	6.24	3.36
5.84	7.0	0.0	2.39	2.09	2.25	4.10	6.25	3.35
5.83	5.1	0.0	2.40	2.08	2.25	4.12	6.19	3.29
5.79	6.1	0.0	2.40	2.09	2.25	4.09	6.32	3.37
5.77	5.1	0.0	2.40	2.08	2.25	4.18	6.20	3.35
5.76	4.0	0.0	2.40	2.09	2.25	4.12	6.20	3.32
5.76	3.2	0.0	2.40	2.08	2.26	4.20	6.20	3.37
5.75	4.7	0.0	2.40	2.08	2.25	4.15	6.19	3.33
5.72	7.0	0.0	2.39	2.09	2.25	4.05	6.32	3.37
5.65	2.6	0.0	2.40	2.09	2.25	4.16	6.22	3.35
5.61	4.0	0.0	2.40	2.09	2.25	4.22	6.18	3.35
5.55	5.0	0.0	2.40	2.09	2.25	4.18	6.20	3.35
5.45	4.6	0.0	2.40	2.09	2.25	4.13	6.24	3.33
5.31	6.5	0.0	2.40	2.09	2.25	4.11	6.35	3.40
4.86	4.0	0.0	2.40	2.09	2.25	4.16	6.18	3.31

Table S54. Energetic, structural, and electronic properties of $(La_2Ce_2O_7)_2$ nanoclusters subjected to a single O-vacancy formation include the following parameters: vacancy energy (E_{vac} in eV), distance of the vacancy from the nanoclusters center of gravity (d_{vac}^{gc} in Å), total magnetic moment (m_{tot} ,in μ_B), average bond length distances for La, Ce, and O (d_{av}^{La}, d_{av}^{B}, and d_{av}^{O} in Å, respectively), average effective coordination numbers for La, Ce, and O ($\mathrm{ECN}_{av}^{\mathrm{La}}$, $\mathrm{ECN}_{av}^{\mathrm{B}}$, and $\mathrm{ECN}_{av}^{\mathrm{O}}$ in NNN, respectively).

E_{vac}	d_{vac}^{cg}	m_{tot}	d_{av}^{La}	d_{av}^{B}	d_{av}^{O}	$\mathrm{ECN}_{av}^{\mathrm{La}}$	$\mathrm{ECN}_{av}^{\mathrm{B}}$	$\mathrm{ECN}_{av}^{\mathrm{O}}$
4.08	2.9	2	2.32	2.18	2.27	4.08	4.46	2.92
3.91	2.8	2	2.32	2.15	2.24	3.88	3.85	2.50
2.95	2.3	2	2.32	2.21	2.27	4.12	4.27	2.79
2.42	4.2	2	2.31	2.20	2.26	4.09	4.18	2.71
2.41	4.0	2	2.29	2.23	2.27	4.10	4.35	2.79
2.40	3.9	2	2.30	2.21	2.27	4.08	4.33	2.80
2.13	1.7	2	2.28	2.20	2.25	3.60	4.21	2.53
2.08	2.9	2	2.32	2.18	2.28	4.08	4.30	2.82
1.89	1.7	2	2.29	2.20	2.26	3.83	4.19	2.63
1.60	2.8	2	2.33	2.20	2.29	4.14	4.45	2.90
1.43	5.3	2	2.33	2.20	2.28	4.13	4.33	2.81

Table S55. Energetic, structural, and electronic properties of $(La_2Ce_2O_7)_4$ nanoclusters subjected to a single O-vacancy formation include the following parameters: vacancy energy (E_{vac} in eV), distance of the vacancy from the nanoclusters center of gravity (d_{vac}^{gc} in Å), total magnetic moment (m_{tot} ,in μ_B), average bond length distances for La, Ce, and O (d_{av}^{La}, d_{av}^{B}, and d_{av}^{O} in Å, respectively), average effective coordination numbers for La, Ce, and O ($\mathrm{ECN}_{av}^{\mathrm{La}}$, $\mathrm{ECN}_{av}^{\mathrm{B}}$, and $\mathrm{ECN}_{av}^{\mathrm{O}}$ in NNN, respectively).

E_{vac}	d_{vac}^{cg}	m_{tot}	d_{av}^{La}	d_{av}^{B}	d_{av}^{O}	$\mathrm{ECN}_{av}^{\mathrm{La}}$	$\mathrm{ECN}_{av}^{\mathrm{B}}$	$\mathrm{ECN}_{av}^{\mathrm{O}}$
3.08	2.3	2	2.37	2.26	2.36	4.66	5.85	3.55
2.93	3.9	2	2.35	2.27	2.36	4.65	6.05	3.64
2.81	4.0	2	2.36	2.27	2.36	4.69	6.07	3.67
2.80	4.7	2	2.36	2.27	2.36	4.69	6.07	3.67
2.67	5.1	2	2.36	2.27	2.37	4.74	6.11	3.72
2.32	2.4	2	2.35	2.27	2.37	4.79	6.16	3.76
2.21	5.2	2	2.36	2.28	2.37	4.79	6.16	3.75

Table S56. Energetic, structural, and electronic properties of $(La_2Ce_2O_7)_6$ nanoclusters subjected to a single O-vacancy formation include the following parameters: vacancy energy (E_{vac} in eV), distance of the vacancy from the nanoclusters center of gravity (d_{vac}^{gc} in Å), total magnetic moment (m_{tot} ,in μ_B), average bond length distances for La, Ce, and O (d_{av}^{La}, d_{av}^{B}, and d_{av}^{O} in Å, respectively), average effective coordination numbers for La, Ce, and O ($\mathrm{ECN}_{av}^{\mathrm{La}}$, $\mathrm{ECN}_{av}^{\mathrm{B}}$, and $\mathrm{ECN}_{av}^{\mathrm{O}}$ in NNN, respectively).

E_{vac}	d_{vac}^{cg}	m_{tot}	d_{av}^{La}	d_{av}^{B}	d_{av}^{O}	$\mathrm{ECN}_{av}^{\mathrm{La}}$	$\mathrm{ECN}_{av}^{\mathrm{B}}$	$\mathrm{ECN}_{av}^{\mathrm{O}}$
3.33	5.8	0.0	2.40	2.22	2.35	4.79	5.61	3.44
3.06	2.7	2.0	2.39	2.23	2.34	4.66	5.49	3.32
3.03	6.7	2.0	2.38	2.23	2.35	4.72	5.66	3.41
2.98	5.4	2.0	2.37	2.24	2.34	4.63	5.62	3.35
2.87	5.1	2.0	2.39	2.22	2.35	4.78	5.59	3.44
2.76	3.2	2.0	2.38	2.24	2.35	4.72	5.66	3.44
2.74	4.7	2.0	2.38	2.24	2.35	4.73	5.63	3.42
2.52	3.6	2.0	2.39	2.23	2.35	4.77	5.62	3.43
2.51	6.2	2.0	2.38	2.23	2.35	4.67	5.60	3.37
2.50	5.1	2.0	2.38	2.23	2.34	4.67	5.60	3.34
2.47	6.3	2.0	2.39	2.23	2.35	4.86	5.68	3.52
2.37	5.7	2.0	2.39	2.24	2.35	4.71	5.75	3.48
2.28	4.7	2.0	2.38	2.23	2.35	4.68	5.63	3.38
2.20	5.5	2.0	2.37	2.25	2.35	4.84	5.70	3.51
2.11	4.3	2.0	2.39	2.24	2.35	4.79	5.77	3.54
2.02	5.2	2.0	2.39	2.25	2.36	4.73	5.87	3.53
1.94	3.6	2.0	2.38	2.24	2.34	4.78	5.57	3.37
1.93	2.6	2.0	2.39	2.23	2.34	4.80	5.49	3.36
1.83	4.8	2.0	2.39	2.23	2.35	4.77	5.63	3.44
1.81	4.4	2.0	2.38	2.25	2.35	4.77	5.71	3.45
1.80	7.1	2.0	2.38	2.25	2.35	4.77	5.72	3.45
1.79	1.2	0.0	2.38	2.25	2.36	4.89	5.70	3.57
1.78	5.8	2.0	2.38	2.25	2.36	4.82	5.77	3.51
1.77	4.5	2.0	2.38	2.24	2.35	4.78	5.64	3.43
1.56	2.3	2.0	2.39	2.25	2.36	4.92	5.77	3.61

Table S57. Energetic, structural, and electronic properties of $(La_2Ce_2O_7)_8$ nanoclusters subjected to a single O-vacancy formation include the following parameters: vacancy energy (E_{vac} in eV), distance of the vacancy from the nanoclusters center of gravity (d_{vac}^{gc} in Å), total magnetic moment (m_{tot} ,in μ_B), average bond length distances for La, Ce, and O (d_{av}^{La}, d_{av}^{B}, and d_{av}^{O} in Å, respectively), average effective coordination numbers for La, Ce, and O ($\mathrm{ECN}_{av}^{\mathrm{La}}$, $\mathrm{ECN}_{av}^{\mathrm{B}}$, and $\mathrm{ECN}_{av}^{\mathrm{O}}$ in NNN, respectively).

E_{vac}	d_{vac}^{cg}	m_{tot}	d_{av}^{La}	d_{av}^{B}	d_{av}^{O}	$\mathrm{ECN}_{av}^{\mathrm{La}}$	$\mathrm{ECN}_{av}^{\mathrm{B}}$	$\mathrm{ECN}_{av}^{\mathrm{O}}$
3.76	6.1	2.0	2.39	2.25	2.37	5.01	5.98	3.70
3.71	3.6	0.0	2.38	2.25	2.37	4.86	5.98	3.64
3.16	4.3	2.0	2.39	2.25	2.37	5.00	5.99	3.72
3.08	7.0	2.0	2.39	2.26	2.38	5.07	6.02	3.77
3.08	3.7	2.0	2.39	2.25	2.37	5.02	5.90	3.65
3.07	3.4	2.0	2.39	2.25	2.37	5.01	5.97	3.72
3.04	5.6	0.0	2.40	2.25	2.38	5.11	6.03	3.79
2.99	0.8	2.0	2.40	2.25	2.37	5.02	5.98	3.71
2.95	4.9	2.0	2.40	2.25	2.37	5.08	5.93	3.70
2.90	6.2	2.0	2.39	2.25	2.37	5.07	5.98	3.75
2.79	4.9	2.0	2.38	2.25	2.37	4.99	6.00	3.74
2.76	7.1	2.0	2.40	2.25	2.37	5.10	5.98	3.74
2.67	4.2	2.0	2.40	2.25	2.37	5.08	5.98	3.74
2.67	4.1	2.0	2.39	2.26	2.37	5.06	5.97	3.74
2.67	5.1	2.0	2.40	2.25	2.38	5.09	6.01	3.77
2.66	5.5	2.0	2.38	2.26	2.37	4.99	5.99	3.71
2.64	7.6	2.0	2.39	2.25	2.38	5.10	6.01	3.76
2.58	5.2	2.0	2.40	2.25	2.37	5.07	5.91	3.68
2.57	4.7	2.0	2.39	2.26	2.38	5.06	6.04	3.75
2.53	6.3	2.0	2.40	2.25	2.38	5.08	6.03	3.75
2.51	3.6	2.0	2.39	2.25	2.38	5.09	6.03	3.75
2.48	5.2	2.0	2.40	2.25	2.38	5.11	6.03	3.79
2.46	7.7	2.0	2.38	2.25	2.36	4.94	5.93	3.64
2.42	3.2	0.0	2.39	2.25	2.37	5.09	5.90	3.70
2.39	5.7	2.0	2.39	2.26	2.38	5.11	6.12	3.84
2.27	4.5	0.0	2.39	2.25	2.37	5.09	5.92	3.72
2.23	6.6	2.0	2.39	2.26	2.37	4.99	5.99	3.70
2.19	5.9	2.0	2.40	2.25	2.38	5.11	6.04	3.76
2.19	6.9	2.0	2.39	2.26	2.38	5.12	6.06	3.81
2.13	4.0	2.0	2.39	2.25	2.38	5.11	6.07	3.84
2.10	3.2	0.0	2.40	2.25	2.37	5.05	5.97	3.72
2.09	2.2	2.0	2.39	2.25	2.38	5.07	6.02	3.77
2.05	7.3	2.0	2.38	2.26	2.37	5.00	5.98	3.71
2.01	5.3	2.0	2.40	2.25	2.38	5.11	6.00	3.75
2.00	4.9	2.0	2.39	2.25	2.37	5.07	5.96	3.72
1.99	3.5	0.0	2.39	2.26	2.38	5.07	6.04	3.78
1.97	5.8	2.0	2.39	2.25	2.37	5.06	5.94	3.70
1.96	2.5	0.0	2.39	2.26	2.37	5.09	6.03	3.75
1.63	6.5	2.0	2.39	2.26	2.37	5.06	6.04	3.73
1.33	5.8	2.0	2.39	2.26	2.37	5.04	5.98	3.68

Table S58. Energetic, structural, and electronic properties of $(La_2Ce_2O_7)_{10}$ nanoclusters subjected to a single O-vacancy formation include the following parameters: vacancy energy (E_{vac} in eV), distance of the vacancy from the nanoclusters center of gravity (d_{vac}^{gc} in Å), total magnetic moment (m_{tot} ,in μ_B), average bond length distances for La, Ce, and O (d_{av}^{La}, d_{av}^{B}, and d_{av}^{O} in Å, respectively), average effective coordination numbers for La, Ce, and O ($\text{ECN}_{av}^{\text{La}}$, $\text{ECN}_{av}^{\text{B}}$, and $\text{ECN}_{av}^{\text{O}}$ in NNN, respectively).

E_{vac}	d_{vac}^{cg}	m_{tot}	d_{av}^{La}	d_{av}^{B}	d_{av}^{O}	$\text{ECN}_{av}^{\text{La}}$	$\text{ECN}_{av}^{\text{B}}$	$\text{ECN}_{av}^{\text{O}}$
3.97	6.2	2.0	2.38	2.26	2.38	4.81	6.33	3.80
3.66	5.8	2.0	2.39	2.26	2.38	4.88	6.33	3.86
3.32	5.7	2.0	2.39	2.26	2.38	4.87	6.29	3.81
3.14	4.6	0.0	2.39	2.26	2.37	4.82	6.25	3.77
3.12	2.4	0.0	2.40	2.26	2.38	4.94	6.27	3.82
2.95	5.7	2.0	2.40	2.26	2.38	4.92	6.40	3.90
2.95	3.2	2.0	2.40	2.26	2.38	4.90	6.26	3.80
2.92	3.2	0.0	2.39	2.26	2.37	4.86	6.24	3.76
2.85	6.4	0.0	2.39	2.26	2.38	4.87	6.31	3.83
2.81	4.3	2.0	2.39	2.26	2.38	4.84	6.31	3.81
2.75	4.1	0.0	2.39	2.25	2.37	4.83	6.20	3.73
2.69	3.7	2.0	2.40	2.26	2.39	5.06	6.36	3.94
2.66	2.9	0.0	2.40	2.26	2.39	5.04	6.36	3.95
2.52	6.5	2.0	2.39	2.26	2.39	4.96	6.41	3.93
2.51	7.1	2.0	2.39	2.26	2.38	4.89	6.30	3.82
2.48	4.7	2.0	2.39	2.26	2.38	4.87	6.32	3.81
2.44	5.1	0.0	2.39	2.27	2.39	4.93	6.44	3.95
2.40	4.7	2.0	2.39	2.26	2.38	4.87	6.31	3.83
2.32	6.1	2.0	2.39	2.26	2.37	4.85	6.25	3.75
2.31	3.6	0.0	2.39	2.27	2.39	5.04	6.47	4.03
2.30	3.3	0.0	2.39	2.26	2.37	4.85	6.25	3.75
2.25	6.1	2.0	2.40	2.26	2.38	4.92	6.41	3.91
2.20	7.7	2.0	2.39	2.26	2.37	4.87	6.28	3.77
2.14	5.4	2.0	2.40	2.26	2.39	5.00	6.41	3.98
2.12	6.6	2.0	2.39	2.27	2.38	4.90	6.35	3.87
2.06	7.4	2.0	2.39	2.27	2.38	4.89	6.42	3.90
2.00	6.8	2.0	2.39	2.26	2.37	4.78	6.34	3.77
1.99	7.6	2.0	2.39	2.26	2.37	4.77	6.33	3.76
1.98	2.9	2.0	2.39	2.26	2.38	4.95	6.33	3.87
1.89	5.5	2.0	2.40	2.27	2.39	4.95	6.43	3.93
1.85	5.1	2.0	2.39	2.26	2.38	4.91	6.32	3.83
1.84	2.7	2.0	2.39	2.26	2.38	4.88	6.34	3.82
1.79	4.2	2.0	2.40	2.26	2.38	4.93	6.38	3.88
1.77	7.5	2.0	2.38	2.27	2.37	4.83	6.32	3.76

References

1 Humphrey, W.; Dalke, A.; Schulten, K. VMD: Visual Molecular Dynamics. *J. Mol. Graph. Model.* **1996**, *14*, 33–38, DOI: 10.1016/0263-7855(96)00018-5.

2 de Mendonça, J. P. A.; Lourenço, T. C.; Freitas, L. P. M.; Santo, A. A. E.; Feliciano, G. T.; Da Silva, J. L. F. Molecular Dynamics Investigation of the Structural and Energetic Properties of CeO_2–MO_x (M = Gd, La, Ce, Zr) Nanoparticles. *Mater. Adv.* **2021**, *2*, 7759–7772, DOI: 10.1039/d1ma00543j.
